# Current emission vs. legacy organic pollutants: Assessing the extent to which the eco-exposome of caged fish reflects external exposure^☆^

**DOI:** 10.1016/j.envpol.2025.126808

**Published:** 2025-07-10

**Authors:** Janek P. Dann, Gerald T. Ankley, Brett R. Blackwell, Beate I. Escher, Annika Jahnke, Kathleen M. Jensen, Correne Jenson, Martin Krauss, Stefan Scholz, Theo Wernicke, Werner Brack

**Affiliations:** a Helmholtz Centre for Environmental Research GmbH – UFZ, Leipzig, 04318, Germany; b Institute of Ecology, Diversity and Evolution, Goethe University Frankfurt, Frankfurt am Main, 60438, Germany; c United States Environmental Protection Agency, Office of Research and Development, Great Lakes Toxicology and Ecology Division (GLTED), 6201 Congdon Blvd, Duluth, 55804, MN, USA; d Environmental Toxicology, Department of Geosciences, Eberhard Karls University Tübingen, Tübingen, 72076, Germany; e Institute for Environmental Research, RWTH Aachen University, Aachen, 52074, Germany; f Umweltbundesamt, UBA, Dessau-Roßlau, 06844, Germany

**Keywords:** Eco-exposome, Target screening, Micropollutants, Aquatic ecosystems, Fish, Contamination patterns

## Abstract

The eco-exposome represents the totality of chemicals present in an organism. To understand how internal exposure of fish relates to chemicals originating from external media (water, sediment), we conducted a 21-d caging study using fathead minnow (*Pimephales promelas*, FHM) as model species. Four sites in/at Lake Superior were chosen that reflect sources of two broad groups of environmental contaminants: Two pond sites with a legacy contamination by persistent organic pollutants (POPs), one site close to a wastewater treatment plant (WWTP) outlet showing more recent and regularly discharged compounds, and one creek expected to show a mixed contamination from both compound groups. We determined total water concentrations, freely dissolved concentrations in sediment pore water, and the FHM’s body burden of organic micropollutants after 2 and 21 d of exposure. Of the 456 target compounds analyzed in FHM, 123 were quantified in water, 165 in sediment and 100 in FHM tissue samples. Chemical profiles and concentrations at the different study locations varied according to their classification as legacy or recently contaminated sites, with the site impacted by the WWTP showing the highest overall concentrations. Only 77 substances quantified in FHM were also detected in water and/or sediment and after applying additional quality and consistency measures, 37 of the 100 substances detected in FHM could not be directly linked to water and/or sediment. Therefore, chemical concentrations in water and sediment cannot simply predict the eco-exposome in fish, which underscores the need of body burden analysis to comprehensively understand an organism’s exposure.

## 1. Introduction

Complex mixtures of environmental contaminants can have severe impacts on human and environmental health from the local to the global scale (Brack et al., 2022). As the internal concentrations of chemicals are toxicologically relevant, the *exposome* concept has gained popularity, which describes the totality of chemicals present in the tissues of an organism (Escher et al., 2017; Rappaport, 2011). Exposome characterization focuses on internal exposure resulting from exogenous contamination with chemicals as well as endogenous chemicals and processes. This concept stemming from human health has recently been adopted for aquatic organisms, proposing an *eco-*exposome approach to understand exposure to ever-changing complex mixtures of contaminants in organisms and ecosystems, with a specific focus on linking internal mixture exposure to adverse effects (Scholz et al., 2022). It is well accepted that biological effects require the uptake of the toxicant and its occurrence at the site of action. This process is part of each compound’s toxicokinetics and has been investigated in fish before (Stadnicka et al., 2012).

The exposome is contrasted with external exposure measurable in surrounding matrices such as water and sediments. External exposure is typically easier to analyze as larger sample volumes can be taken, the impact of matrix effects is lower, and ethical concerns are circumvented. A large number of monitoring studies on complex chemical mixtures in the environment has generated substantial knowledge about hundreds of chemicals present in water (Finckh et al., 2022; Finckh et al., 2024; Gago-Ferrero et al., 2020) and sediments (Massei et al., 2018; Muz et al., 2020). At the same time, screening of aquatic organisms for internal mixture exposure to a broader range of chemicals is still limited to few studies involving invertebrates and fish (Grabicova et al., 2015; Inostroza et al., 2016; Yang et al., 2015) with a focus on pesticides and pharmaceuticals (Miller et al., 2018). Particularly limited are comparative studies investigating both internal dose and external exposure (Inostroza et al., 2017; Kandie et al., 2020). Although bioaccumulation of hydrophobic persistent organic pollutants (POPs) and polycyclic aromatic hydrocarbons (PAHs) in aquatic organisms has been well-investigated (Baumard et al., 1998; Houde et al., 2011; Jamieson et al., 2017), it remains in many cases unclear how external exposure to mixtures of micropollutants, comprising a broader range of compound groups with diverse physico-chemical properties, might translate into internal exposure. Recent studies have worked towards understanding the partitioning of very hydrophobic compounds in suspended particulate matter (SPM), water and biota (Wernicke et al., 2022a; Wernicke et al., 2022b), but exposure of fish was still mostly characterized by external concentrations.

In the current study, we investigated to which degree mixtures detectable in surrounding water and sediment would reflect the internally measurable exposome in fish, and to which extent the two measures would produce redundant or complementary information. Since this might strongly depend on the type of contaminants under consideration, we selected four sites in Lake Superior with distinctively different contamination characteristics. The sites were dominated either by (i) hydrophobic legacy compounds, including many POPs, which are predominantly associated with sediments, or (ii) more polar and less persistent chemicals continuously discharged into water from wastewater treatment plants (WWTPs). To compare the impact of long-term vs. short-term exposure on the measurable fish exposome, we assessed exposure times of 2 and 21 days. In order to achieve controlled experimental conditions we used fish caging, which has been demonstrated to be a powerful alternative to sampling indigenous organisms (Ankley et al., 2008). The species we employed, fathead minnow (*Pimephales promelas,* FHM), is an excellent model organism in caging experiments for assessing bioconcentration (Lefebvre et al., 2017) and risks related to complex mixtures of contaminants in water bodies (Ankley et al., 2021; Jasinska et al., 2015; Lazaro-Cote et al., 2018).

## 2. Material and methods

### 2.1. Sites

The experiments were performed at four sites in St. Louis Bay close to Duluth (MN, USA), impacted by different sources such as WWTP effluent, urban and industrial runoff (Corsi et al., 2019). Two sites were located in the Erie Pier Ponds (North and South, hereafter called “Pond N″ and “Pond S″), which were historically contaminated (Christensen et al., 2012) with elevated sediment concentrations of e.g. polycyclic aromatic hydrocarbons (PAHs) and polychlorinated biphenyls (PCBs). One site was located close to the wastewater discharge of the Western Lake Superior Sanitary District (called “WWTP”) and was expected to be impacted by discharges of more recent contaminants such as pharmaceuticals, pesticides and personal care products (Focazio et al., 2008; Venkatesan and Halden, 2014). The fourth site was in the Shopper’s Creek (“Creek”), which flows into Pond S and might show aspects of both “types” of contamination, as it is connected to one of the legacy sites, but also impacted by street runoff and urban discharge. Site locations, sampling information and water quality characteristics are described in the Supplementary Materials (SM) (SM-A, Section S1 and SM-B, Table S1).

### 2.2. Chemicals

Information about chemicals and analytical standards is provided in SM-A, Section S2 (all compounds – including additional ones which were not measurable in fish), SM-B, Table S2a (target compounds), and SM-B, Table S2b (internal standards).

### 2.3. Caged fish deployment

FHMs were obtained from an on-site culture facility at the US Environmental Protection Agency, Great Lakes Toxicology and Ecology Division (GLTED) in Duluth, MN, USA. All experimental procedures using fish were reviewed and approved by the Animal Care and Use Committee, in accordance with the Animal Welfare Act and Interagency Research Animal Committee guidelines. Four cages were placed at every sampling site, each containing four adult female and four adult male FHMs. Two cages per site were retrieved after 2 d of exposure, another two after 21 d, and the wet weight of every fish was recorded. Sexually mature adult fish rather than juveniles were used for this study for several reasons, including the need for larger fish to ensure no loss of animals during *in situ* exposures (i.e., through the cage mesh) and to provide adequate biomass for chemical analysis. For more details concerning fish survival and sample amounts see SM-A, Section S3a and SM-B, Table S5a–b. Water quality characteristics from the sites and the laboratory controls are provided in SM-B, Table S1.

### 2.4. Water and sediment sampling

During caged fish deployment, automated water samplers (Kahl et al., 2014) were installed alongside the cages and programmed to collect 25 mL of water at 10-min intervals for the duration of the exposure period. Sampling time points were 2, 7, 14 and 21 d after deployment. Surficial sediment was collected 14 d after deployment of the cages, within 3.5 m from each caging site. Bioavailable chemical concentrations in sediment pore waters were determined by passive equilibrium sampling with silicone-coated glass jars equilibrated for 21 d as established by (Reichenberg et al., 2008; Jahnke et al., 2012; Muz et al., 2020). Further details are provided in SM-A; Section S3b.

### 2.5. Sample preparation

#### Tissue.

FHM from each cage were sorted by sex (SM-B, Table S5a and b) and homogenized (details in SM-A, Section S3c). Due to mortality in some cases and varying mass of single FHM, four to six fish were pooled to reach a mass of ca. 4 g per sample for extraction (exact numbers are given in SM-B, Table S5c). Another aliquot of 500 mg of each sample was used to determine the lipid content gravimetrically (Smedes, 1999). For extraction and cleanup, a modified QuEChERS method (Anastassiades et al., 2003) using two cleanup sorbents in parallel (primary-secondary amine, PSA; Agilent; C_18_-modified silica gel, C18; Agilent) was applied to about 4 g of the fish homogenate. More details are given in SM-A, Section S4a.

#### Fish food.

To consider the FHM maintenance food as possible source of chemicals in the fish, samples of food from the GLTED culture facility (adult brine shrimp and crumble feed) were extracted and analyzed with the same method as the FHM. More information is given in SM-A, Section S4a.

#### Water.

Water samples were subjected to solid-phase extraction (SPE) on Oasis HLB cartridges (500 mg sorbent; Waters), after filtration using glass fiber filters (Whatman). For further details see SM-A, Section S4b.

#### Sediments.

These are known to contain different fractions of sorbed molecules with fast to very slow desorption kinetics (Cornelissen et al., 1997) and only the readily desorbable fraction of chemicals in sediments is considered as “bioavailable” (Semple et al., 2004). To account for this, the bioavailable sediment contamination was measured using silicone passive equilibrium sampling (Jahnke et al., 2018; Muz et al., 2020). This was conducted using glass jars coated with a μm-thin layer of DC1–2577 silicone in three levels of thickness using the method of (Jahnke et al., 2012) as explained in detail in SM-A, Section S4c. Water content and exact wet weights of the samples can be found in SM-B, Table S4a. All measured sediment concentrations were normalized to the mass of silicone in each jar. Additionally, the fraction of organic carbon (OC) in the sediment was measured (SM-B, Table S4c) to transform *c*_silicone_ to *c*_OC_ via the following equations (Jahnke et al., 2012), using a constant *K*_PDMS/OC_ (Li et al., 2013). PDMS stands for purified polydimethylsiloxane, which has similar sorption properties as silicone:

(1) $$c_{\text{OC}}=c_{\text{silicone}}\times K_{\text{PDMS/OC}}$$

and

(2) $$\log_{10}K_{\text{PDMS/OC}}=0.32$$

### 2.6. Instrumental analysis

All extracts were analyzed by LC-HRMS for 357 target compounds; FHM and sediment extracts were additionally analyzed by GC-HRMS for 99 target compounds. Detailed information about parameters and settings is given in the SM-A, Section S5c for LC-HRMS and in SM-A, Section S5d for GC-HRMS.

### 2.7. Data evaluation

The LC-HRMS and GC-HRMS raw files were converted to mzML format with ProteoWizard (Chambers et al., 2012) and processed for peak picking, alignment, gap filling and peak annotation with MZmine 2.38 (LC data) or MZmine 2.39 (GC data) (Pluskal et al., 2010), for details see SM-A, section S5e-f. The data was further processed using the in-house R package *MZquant* for automated quantification. For details, see (Schulze et al., 2021). For the creation of graphs, the *ggplot2* package (Wickham, 2009) in RStudio was used.

## 3. Results

### 3.1. Overview of detected chemicals

A total of 317 individual substances were detected in at least one of the analyzed matrices. A subset of 155 substances (out of 515 measurable in this matrix) were quantified in water extracts (SM-B, Table S3a–b), 194 (out of 635 measurable) in sediment extracts (SM-B, Table S4d–e) and 142 (out of 456 measurable) in FHM extracts (SM-B, Table S5d–h). Considering only the 456 chemicals, for which the method allowed analysis in FHM (target compounds), 123 were found in water and 165 in sediment. Substances which were not measurable in the FHM were excluded from detailed discussion; information can be found in the SM (SM-B, Tables S3a-b, S4d-e, S5d-h).

**Fig. 1.**
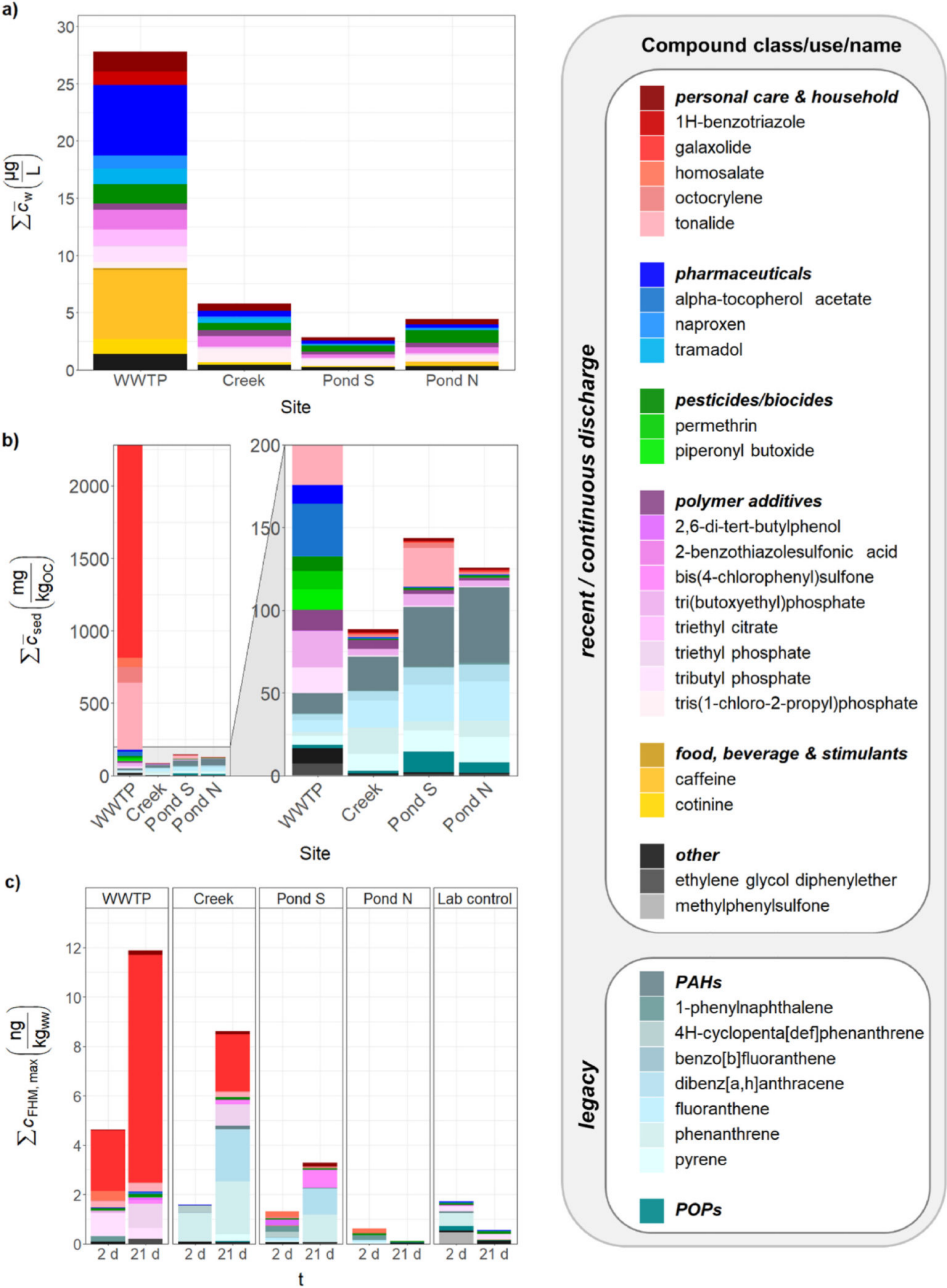
Measured concentrations in a) water (μg/L), b) sediment (mg/kg_OC_) and c) FHM (2 and 21 d, ng/kg_ww_) at the different sites WWTP, Creek, Pond S and Pond N. Individual compounds are plotted separately in the figures if at least one sample exceeded 1 μg/L in water, 10 mg/kg_OC_ in sediment or 0.2 ng/kg_ww_ in FHM to highlight substances detected with relatively high concentrations. In case of FHM, the lab controls are also displayed. Corresponding data can be found in SM-B, Tables S2c, S3a-b, S4d-e, S5d-f.

The 456 target compounds were grouped into eight categories (SM-B, Table S2c), describing their main usage, source, and/or characteristic chemical substructures. The applied categories are *personal care/household* (in total 21 compounds measured//8 detected in water/16 in sediment/11 in FHM//a total of 18 compounds detected in at least one matrix), *pharmaceuticals* (106//34/28/24//63), *POPs* (36//0/17/13//22), *polymer additives* (29//12/18/14//22), *PAHs* (21//0/19/16//20), *pesticides/biocides* (179//39/40/53//86), *food, beverage & stimulants* (13//7/2/5//10), and *other* (51//23/25/10//37). Examples for substances in the category *other* are perfluoroalkyl acids, intermediates, dyes and solvents. An overview of substances quantified in water, sediment and FHM (including lab controls) is shown in Fig. 1. Individual compounds are highlighted, if at least one sample exceeded 1 μg/L in water, 10 mg/kg_OC_ in sediment or 0.2 ng/kg_ww_ in FHM.

### 3.2. Water concentrations

In the water samples, 123 of the 456 target compounds (SM-B, Table S3b) were detected. Their occurrence at different sites and the sum of concentrations are depicted in Fig. 1a. Concentrations of some chemicals varied slightly during the three weeks of caging; which is discussed in SM-A, Section S6 (see also SM-B, Table S3a). Overall, 114 target compounds were detected at WWTP, 85 at Creek, 77 at Pond S, and 82 at Pond N, with a maximum concentration at WWTP for 79 of these compounds (versus 20 with a maximum at Creek, four at Pond S, and 14 at Pond N). WWTP was the site with the highest cumulative concentration for every compound class except *POPs*. A total of 62 compounds were found at all four sites, consisting of 14 *pharmaceuticals*, 14 *pesticides/biocides*, eight *polymer additives*, five *personal care/household*, two *food, beverage & stimulants*, and 19 *other* chemicals (also see SM-A, Section S9). There were 18 compounds solely detected at WWTP, with the largest number comprising eleven *pharmaceuticals*. One compound was solely found at Creek, one in the overlap Pond S/Creek and two in the overlap Pond N/Pond S/Creek.

### 3.3. Sediment concentrations

In the silicone extracts, 165 out of the 456 target compounds could be detected. The silicone concentrations were converted to OC-normalized sediment concentrations with eq. (1). Data were excluded if the concentrations determined for the three different thicknesses of silicone showed a difference of ≥90 % between the smallest and largest value, which indicates that the chemical mass in the silicone was too variable to yield a valid value for *c*_sed_; this is referred to as the “stability threshold” in SM-B, Table S4d. In total, 133 target compounds were detected at WWTP, 100 at Creek, 128 at Pond S, and 115 at Pond N. Of these, 80 compounds were found at all four sites, consisting of 14 *PAHs*, 11 *personal care/household*, 14 *polymer additives*, nine *pesticides/biocides*, ten *POPs*, nine *pharmaceuticals*, and 13 *other* (see SM-B, Table S4d–e, also SM-A, Section S9). A subset of 29 target compounds was only detected at WWTP: 13 *pharmaceuticals*, eight *pesticides/biocides*, three *personal care/household* and five *other* compounds.

In Fig. 1b, the cumulative concentrations of all detected target compounds at the different sites are displayed, with 12 compounds exceeding 10 mg/kg_OC_ at least at one site. WWTP showed the highest sediment concentrations, with eight substances exceeding 10 mg/kg_OC_ (plotted as individuals in Fig. 1b), whereas four *personal care/household* compounds represented up to 93 % of the total accumulated sediment concentration at this site: the fragrances galaxolide (1466 mg/kg_OC_) and tonalide (464 mg/kg_OC_); and the UV filters octocrylene (112 mg/kg_OC_) and homosalate (61 mg/kg_OC_). Despite the fact that WWTP showed the highest contamination relative to overall cumulative concentration, the maximum sum concentrations for three of the eight compound classes were found at Pond S and/or Pond N: *POPs* (16 compounds, at both Pond S and N), *PAHs* (19 compounds at both Pond S and N), and *other* (19 compounds at Pond S; 16 compounds at Pond N).

### 3.4. FHM tissue concentrations

For the analysis, at each site and time point only the maximum value of all FHM concentrations was used to represent a worst-case scenario of contamination (highest possible body burden). In the QuEChERS extracts of the FHM, 142 of the 456 target compounds were detected, 75 of which were also found in the control fish held in the laboratory. For the 75 substances detected in the controls (SM-B, Table S5e), a detection threshold of two times the maximum concentration in the control fish was established and only concentrations above this level were used for further data evaluation (unless indicated otherwise), which resulted in 42 compounds being excluded from further analysis. The remaining 100 target substances showed maximum concentrations above the control fish threshold at least at one site and time point; 70 in each the 2-d samples and 21-d samples, of which 40 were detected at both time points. Independent of time points, 50 compounds were detected at both Creek and Pond S, 26 at Pond N and 62 at WWTP. Chemical concentrations are reported on the basis of wet weight (ng/kg_ww_; SM-B, Table S5c and d), because we also included ionizable and polar organic chemicals that are not typically associated with the lipid phase. Lipid content of the fish was somewhat variable and sex-dependent (SM-B, Table S5f). Lipid-normalized internal chemical concentrations are described further in SM-A, Section S10 and lipid-normalized concentrations of FHM can be found in Table S5g.

The 75 compounds detected in at least one FHM control (SM-B, Table S5h) could be explained in some cases by high concentrations in the brine shrimp and crumble feed used as fish food (SM-B, Tables S6b and S8), indicating exposure through the food. Presence in FHM at the different sites and sum concentrations are depicted in Fig. S2c and S2d. Wet weights and lipid contents of the analyzed fish food can be found in the SM-B, Table S6a and b.

Five compounds were detected at every site at least at one time point above the control fish threshold, 15 substances were detected only at Creek, and 13 only at WWTP. The highest additive chemical concentration in FHM was found after the 21-d exposure at WWTP, followed by 21-d exposure at Creek (Fig. 1c; SM-B, Table S4). Nine compounds exceeded 0.2 ng/kg_ww_ at least at one site, most of them at Creek and/or WWTP: tonalide (Creek: 0.2 ng/kg_ww_/WWTP: 0.33 ng/kg_ww_), galaxolide (Creek: 2.3 ng/kg_ww_/WWTP: 9.2 ng/kg_ww_); triethyl phosphate (Creek: 0.86 ng/kg_ww_/WWTP: 0.97 ng/kg_ww_); pyrene (Creek: 0.27 ng/kg_ww_), phenanthrene (Creek: 1.1 ng/kg_ww_; Pond S: 2.2 ng/kg_ww_) and dibenz(a,h)anthracene (Creek: 2.1 ng/kg_ww_ and Pond S: 1.1 ng/kg_ww_); bis(4-chlorophenyl)sulfone (Pond S: 0.72 ng/kg_ww_) and tributyl phosphate (WWTP: 0.44 ng/kg_ww_, control 21-d: 0.21 ng/kg_ww_). At Pond N, no substance exceeded 0.2 ng/kg_ww_.

In addition to the compounds mentioned, 33 substances were detected in both 2-d and 21-d FHM at least at one site above the control threshold: two *personal care/household*, eight *pharmaceuticals*, one *POP*, one *polymer additive*, seven *PAHs*, 11 *pesticides/biocides*, three *food, beverage & stimulants*, and two *other*.

In total, 30 substances occurred only in 2-d FHM (above the control threshold): two were detected at Creek, 14 at Pond S, four at Pond N and 15 at WWTP. Additionally, 30 were detected only in 21-d FHM (above the control threshold): 14 were detected at Creek, 11 at Pond S, six at Pond N and 12 at WWTP. More details are given in SM-B, Table S5f.

## 4. Discussion

### 4.1. Occurrence and distribution of specific compound groups

A large variety of compounds was detected in water, sediment and FHM, showing diverse compositions both across matrices and the different sites. The patterns at the sites widely matched the expected contamination characteristics regarding the class of chemicals and the mentioned groups legacy/recent compounds. In all three matrices most of the detected target compounds (133/165 in sediment, 114/123 in water, 62/100 in FHM) as well as the highest sum concentrations (cf. Fig. 1) were measured at the site WWTP. Members of the compound classes *personal care & household*, *pharmaceuticals*, and *food, beverage & stimulants* were predominantly detected at WWTP, matching the expectation that these compounds are continuously emitted with wastewater. Substances of the compound classes *POPs*, *PAHs*, and *pesticides/biocides* were more prominent at the Ponds, the former two correlating with the former known historical contamination. Members of the compound class *polymer additives* occurred similarly at all sites with respect to numbers, composition and concentrations. *Pesticides/biocides* were found in higher concentrations at the Ponds, but occurred also at the other sites. Compared to WWTP and Pond S, Creek showed a combined recent/legacy contamination pattern in the 21-d FHM, with increasing concentrations of fragrances (at WWTP) and PAHs (at Ponds) compared to the 2-d FHM, whereas the composition of micropollutants at this site seemed to be more similar to the Ponds when considering water and sediment contamination. In total, eleven *personal care/household* compounds of 18 detected in at least one matrix (FHM, water, and/or sediment) were detected in FHM above the control threshold. The same was observed for 24/60 *pharmaceuticals*, 13/20 *POPs*, 14/22 *polymer additives*, 16/20 *PAHs*, 53/79 *pesticides/biocides*, 5/10 *food, beverage & stimulants*, and 10/36 *other*.

Regarding water and sediment, Creek, Pond S and N were more similar to one another than to WWTP with respect to both chemical composition and concentrations. The aggregate concentrations in the FHM were dominated by the high and increasing (2–21 d) concentrations of two fragrances (tonalide and galaxolide) and two phosphates (triethyl phosphate and tributyl phosphate) at WWTP, and *PAHs* (pyrene, phenanthrene, and dibenz(a,h)anthracene) at Creek and Pond S.

The decrease of most micropollutant concentrations in the 21-d FHM at pond N might be explained by poor nutrition at the site as implied by the decreasing lipid content (SM-B, Tables S5b,c,f) which might lead to a decrease in concentrations of hydrophobic organic chemicals.

### 4.2. (Co-)occurrence of chemicals in water, sediment, and FHM

A principal component analysis (PCA; SM-A, section S8.1 and SM-B, Table S7a–j) and a correlation analysis (SM-A, section S8.2) were applied to different combinations of subsets of normalized data for FHM, water, and sediment, which implied that the matrices FHM (2d/21d), water, and sediment all show different contamination patterns which hardly overlap (ref. SM-A, Section S8.3). There were many examples of chemicals only found in water (N = 67), only in sediment (N = 74) or in both water and sediment, but not in FHM (N = 24), whereas 23 chemicals were found only in FHM. Overall, 45 substances occurred both in sediment and FHM samples, ten were found in water and in FHM, and 22 substances were found in all three sample types (see SM-A, Section S9, S10 and Table S2). Overall, 15 % of the detected data points in water (note that no GC/HRMS measurement was performed) and 24 % of the detected data points in sediment had a corresponding detection in FHM. Conversely, in total 28 % of detections in FHM overlapped with detected chemicals in water and 60 % with those in sediment.

### 4.3. Comparison of 2-d FHM, 21-d FHM, water and sediment concentrations

#### 4.3.1. Categorization of occurrence

**Fig. 2.**
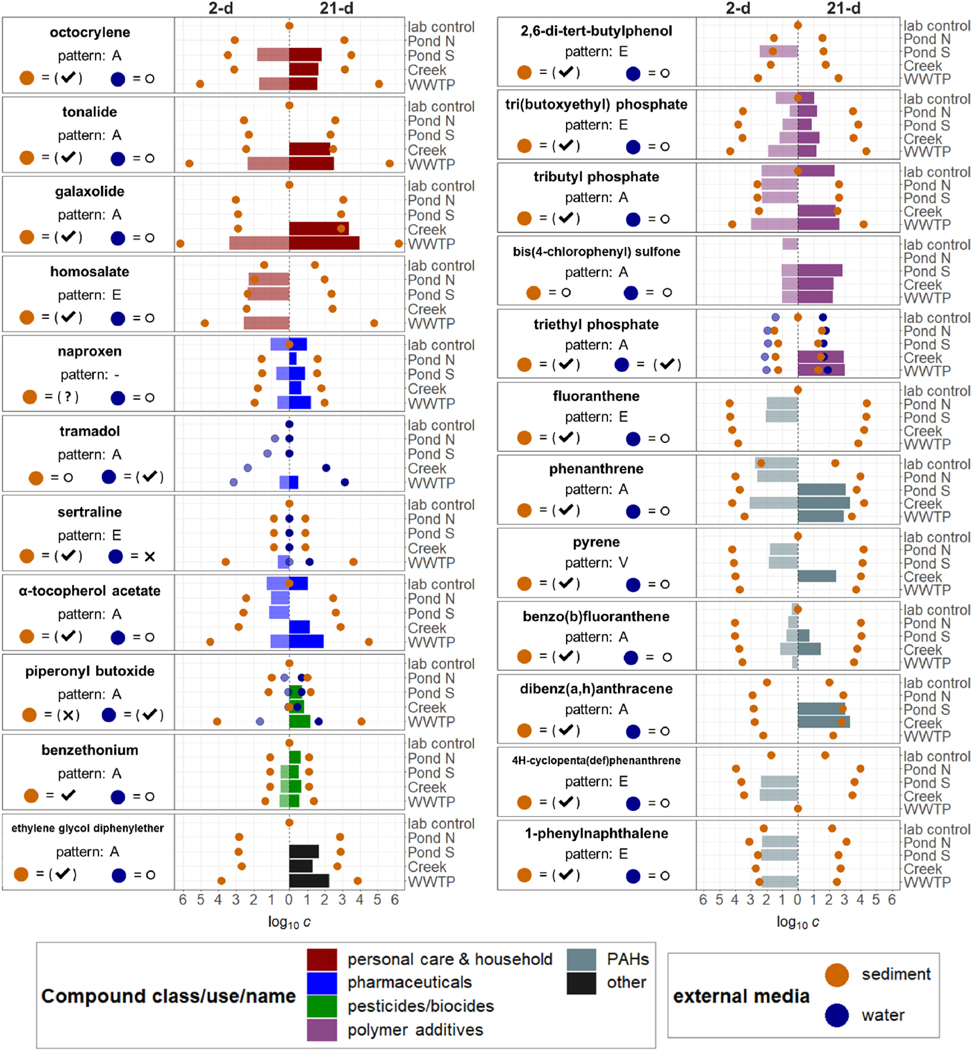
Site- and time-dependent concentrations of highlighted substances in water, sediment and FHM as an example for the applied data evaluation. The left side of each plot represents the 2-d concentrations, the right side the 21-d concentrations in FHM, complemented by the corresponding water concentrations and concentrations detected in the sediment. All concentrations (FHM, water, sediment) were transformed into log_10_ values, except for original values < 1, where log (1+x) was used. Only substances with at least one value above the applied control threshold for FHM are shown, but all values are included independent of this threshold. The bars represent the FHM concentrations, the dots water (blue, both 2-d and 21-d) and sediment (orange, only 21-d) concentrations, the latter are also included in the 2-d side of the plot. The color of the bars displays the compound class/use/name. The assigned pattern (A/E/V) and the agreement between FHM and sediment/water were also added; A: *accumulation* (occurrence in 21-d FHM, possible co-occurrence in 2-d FHM at all sites), E: *elimination* (occurrence in 2-d FHM, no occurrence in 21-d FHM at all sites), V: *variable* (other combinations). The agreement between FHM and water/sediment were grouped in *match*: ✔; *match with missing value(s) in FHM*: (✔); *no match*: × *missing value(s) in water and/or sediment*: (×); and *not detected in water and/or sediment*: ◯. Note that the original concentration units were ng/g_ww_ for FHM, μg/L for water and mg/kg_OC_ for sediment.

Three possible combinations for the occurrence of substances in FHM exist: only in 2-d, only in 21-d or in both 2-d and 21-d samples. In Fig. 2, the 2-d FHM, 21-d FHM, water and sediment concentrations of the most abundant compounds (cf. section 3.1) are displayed for comparison (more Figures and discussion addressing all compounds detected in FHM is in the SM-A, section S11).

The following system was used to group the occurrence patterns in FHM: if a substance was detected only in 2-d FHM, it is classified as E-pattern (E for *elimination* (Gobas and Mackay, 1987)), while detection only in 21-d FHM it is presumed to show *accumulation* potential (referred to as A-pattern). Regarding the substances detected in both 2-d and 21-d FHM at any site, the picture is more complex. The simplest case would be that a compound was detected at both time points at the same site and not at all at any other site. Another similar case would be that a compound was not yet present in 2-d, but in the 21-d FHM of the same site, while at another site it was present at both 2-d and 21-d FHM – both implying the same tendency: an A-pattern over time or at least some degree of persistency. However, there were more ambiguous cases in which a substance was detected in FHM at 2-d but not at 21-d at one site, but at both timepoints or only at 21-d at another site (referred to as V-pattern, V for *variable*). For these cases, fast uptake paired with slow elimination might be an explanation, but method detection limits (MDLs) or decreasing lipid content of the FHM (cf. section 3.1) could also affect the measured occurrences/concentrations. Generally, the applied pattern categorization is established to group the results in a logical manner with respect to the occurrence of substances and not meant to confirm any responsible mechanism(s). As the variation of occurrence in FHM was quite high and the aim of this study was not to investigate kinetics of possible metabolisms, further discussion is limited to semi-quantitative comparisons between the different matrices. There is, of course, some limitation to our A-/E–/V-pattern differentiation in case of small numbers of detects.

The obtained patterns in FHM were compared with the detections in sediment and/or water. It is important to note that the caged fish were in direct contact with the water, but not with the sediment. This means that the water should be a more direct proxy for uptake, whereas the sediment might be a proxy for accumulation potential in general (Jahnke et al., 2014), while also representing a long-term source for contamination of the water column. Regarding the concentrations in water, there might also be cases of transient contamination (e.g., if a substance was only detected in water at 21-d parallel to the FHM) where it is not possible to derive any statements about accumulation potential. However, as mentioned in section 4.1 and shown in SM-A, Sections S7, S10-S11, this kind of discrepancy only concerned few substances. Nevertheless, there were also cases where the occurrence in FHM agreed with the water contamination at both timepoints making it impossible to distinguish between permanent (rather fast) uptake/elimination cycles and actual accumulation.

To reduce the complexity, an abbreviation system for different degrees of agreement between each external matrix (water/sediment) and the FHM regarding occurrence and timepoint was utilized. The comparisons were grouped in *match*: ✔; and *no match*: × between FHM and the concerning matrix at all sites/timepoints. If there was a divergence of *match* and *no match* at single sites/time points, we used (✔) for matches with missing value(s) in FHM at least at one site/time point and (×) for substances with detections in FHM where corresponding detections in the water and/or sediment were missing. For completeness, the substances not detected in the concerning matrix were summarized in the group *not detected*: ○. Substances only detected in either water or sediment (or both) but not in FHM were not included.

#### 4.3.2. Comparison of occurrences

In Fig. 2, the concentrations of the most abundant (see section 3.1) substances in water, sediment, and FHM were compared depending on time and site as an example for A-/E–/V-patterns and the agreement of co-occurrence in water and/or sediment. A reason for non-detects in FHM might be a too high MDL and/or the weight loss in case of pond N (cf. section 3.1). Note that “○ in water” includes substances which were not measured in water due to the lack of GC-MS analysis. A more detailed discussion on the site- and time-dependent abundance of all substances detected in FHM, water, and sediment is conducted in SM-A, section S11.

Unequivocal examples for an A-pattern were octocrylene, tonalide, galaxolide, ethylene glycol diphenylether and dibenz(a,h)anthracene, as they occurred either in 21-d or in both 2-d and 21-d FHM. These substances showed a limited agreement to the abundance in sediment (missing detections in 21-d FHM at least one site; (✔) for sediment, ○ for water). Examples for an E-pattern were the 2,6-di-tert-butylphenol, homosalate, fluoranthene, 4H-cyclopenta(def)phenanthrene and 1-phenylnaphthalene as they occurred only in 2-d FHM, while at least one site a detection of these compounds was missing in the FHM which was detected in sediment at that site ((✔) for sediment, ○ for water). Examples for a V-pattern were pyrene with detections in 21-d FHM at one site and detections in 2-d FHM at another, both matching the occurrence in sediment partially ((✔) for sediment, ○ for water).

A complete match ✔ or complete mismatch × were both very rare. Substances representing these were added to Fig. 2 to display this kind of match/mismatch: An example for ✔ is benzethonium in sediment, which occurred in an A-pattern in FHM at every site and was detected at every site in sediment, while not being detected in water at all (=○). One of the few examples for × is the *pharmaceutical* sertraline, which showed an E-pattern in FHM with limited agreement to sediment (=(✔)), whereas even when the site of detection agreed between water and FHM, the timepoint did not. Naproxen is an example for a substance being excluded from the FHM data evaluation, because the concentrations at the sites did not exceed the FHM control threshold.

To further explore the data, c_FHM_/c_w_ ratios, which can be considered apparent bioconcentration factors, were compared with predicted bioconcentration factors (BCF, SM-A: Section S13). Moreover, c_FHM_/c_sed_ ratios were compared with predicted biota-to-sediment accumulation factors (BSAF, SM-A: Section S13). c_FHM_ was further predicted from c_w_ and c_sed_ by predicted BCF/BSAF as described in the SI (SM-A: Section S14; SM-B: Tables S13 and S14). The comparison of experimental concentration ratios with BCF and BSAF, respectively (SM-A: Section S15), demonstrated that 71 % of the 23 substances detected in 21-d FHM and water and 58 % of the 48 substances detected in 21-d FHM and sediment could be predicted based on equilibrium considerations. Hence, water and sediment proved to be insufficient proxies of the eco-exposome.

#### 4.3.3. Summary agreement of concentration in FHM with that in water and/or sediment

**Fig. 3.**
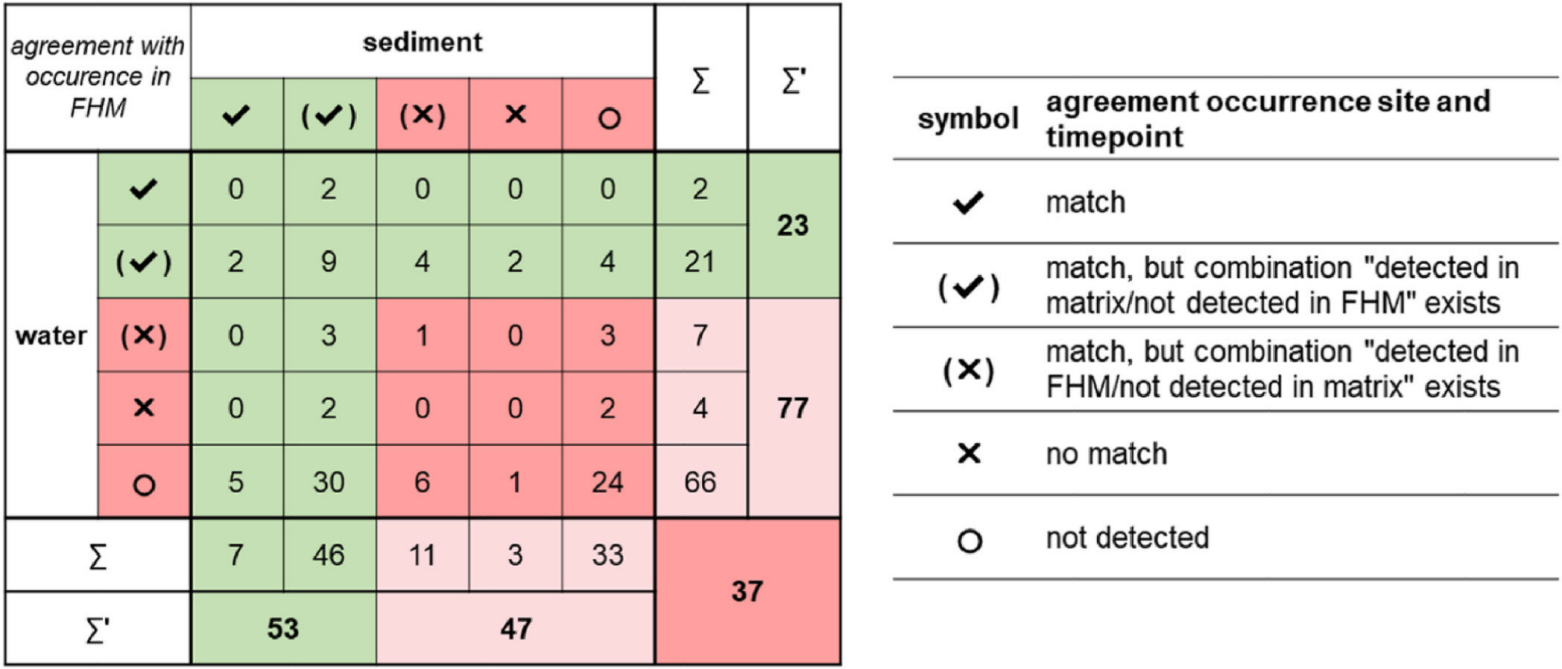
Systematic comparison of the corresponding occurrence of target substances in the external matrices sediment and water by its agreement with the occurrence in FHM regarding site and timepoint.

Fig. 3 summarizes the co-occurrence of compounds in FHM, water and sediment. It is apparent that for many substances occurring in FHM at one site/timepoint, there is a missing concentration in FHM at another site/timepoint, which might be caused by an insufficient analytical MDL for FHM. Nevertheless, 53 substances detected in the FHM matched the occurrence in sediment and 23 in water, when limits of detection are considered for the fish (sum ✔ and (✔) in each matrix). Conversely, 37 substances detected in FHM were at least to some degree not linked to detects in water or sediment (sum (×), ×, and ○ in every matrix). This means that in addition to the 23 substances only detected in FHM (SM-A, S11.1-S11.3), 14 compounds seem to be present in FHM without/before reaching stable or detectable concentrations in water and/or sediment. Thus, a contamination of the FHM with these compounds might be overlooked or underestimated, if only analysis of water and/or sediment is applied. While it might not be surprising for daidzein and harman as natural occurring compounds, it might be relevant for the other substances. This data and a comparison between water and sediment regarding proxy qualities is discussed in SM-A, S12.

### 4.4. Legacy vs. recent micropollutant contamination in FHM

Table 1 summarizes the detects in FHM, grouped by compound class, pattern and site. As expected and discussed in section 3.1, the Ponds and the Creek showed the highest concentrations of legacy compounds like *POPs* and *PAHs*, whereas WWTP represented a regular and recent discharge of micropollutants such as *pharmaceuticals*. This is partly reflected by the findings of these substances in FHM. *POPs* and *PAHs* were mainly detected in FHM at the Ponds and Creek. The substances of the remaining six compound classes had either a maximum number of detects in FHM at WWTP (*personal care & household*, *pesticides/biocides*, *pharmaceuticals*, *polymer additives*) with rather high number of detects also found at Creek, or were more evenly distributed across sites (*food, beverage & stimulants*, *other*). This observation agrees well with the compounds found in water and sediment and the anticipated contamination at these sites. The Creek’s contamination pattern suggests an impact from both legacy and recent contamination. Limitations in our ability to infer a clear result in some cases were due to the absence of some compounds in the 21-d FHM at pond N (cf. section 4.1) as well as in 2-d FHM at Creek (visible in Table 1), resulting in possible false-negatives and potential misclassification regarding the observed patterns. However, the findings of this study agreed well with literature in most cases (more detailed discussion regarding the substances shown in Fig. 2 in SM-A, Section S15).

Comparing the abundance of the “legacy” compounds in FHM, 9/19 (47 %) could be related to detects in sediment. From the “recent” compounds, 46/81 (57 %) could be related to occurrence in sediment and/or water. Combining these results, a total of 54 % of compounds could be related to their occurrences in water, sediment, or both.

## 5. Conclusions

Our study shows that for many chemicals the measured concentrations in the abiotic surrounding media, i.e., water or sediment, do not represent an accurate or even exhaustive proxy of the internal exposure of fish. Although matrix-specific analytical limitations (sample volumes, detection limits, matrix effects) may limit comparability, internal concentrations in fish rather provide complementary information on exposure. Therefore, they are a valuable and important complement for monitoring and an integration of external and internal exposure information for mixture risk assessment is a key future research need.

The measurable *eco-*exposome seems to be substantially affected both by external factors, such as fluctuating exposure concentrations and bioavailability limitations, as well as internal variables, including nutrition or induction of xenobiotic metabolism/excretion. The high diversity of chemicals found in fish in the present study indicates that we know only a fraction of the internal eco-exposome of fish.

## Supplementary Material

Supplement1

Supplement2

## Figures and Tables

**Table 1:** Number of pattern-detects (A and E) per compound class/use, separated by site, found in the FHM, complemented by the reasonable agreements in water and sediment, ✔ or (✔) and the percentage of detected substances explainable by the occurrence in water, sediment or both. The numbers in parentheses represent the count of detects, which showed a V-pattern in comparison of all sites, but the pattern of concern (A, E) at that particular site. Additionally, the sum of detects of each pattern per site is displayed. A: *accumulation* (occurrence in 21-d FHM, possible co-occurrence in 2-d FHM at all sites), E: *elimination* (occurrence in 2-d FHM, no occurrence in 21-d FHM at all sites), V: *variable* (other combinations).

*compound class/use*	*N detected in FHM*	*pattern*	Site				✔ or (✔)		% explained by occurrence(s) in			
			pond N	pond S	Creek	WWTP	water	sediment	water*	sediment	w. and/or sed.

**POPs**	**6**	**A**	2	3	2 (1)	0	–	2 (1)	–	33 (+17)	**33**
		**E**	0	0 (1)	0	0 (1)	–	0 (1)			
**PAHs**	**13**	**A**	0 (1)	5 (2)	6 (4)	1	–	4 (4)	–	54 (+31)	**54**
		**E**	2 (3)	3 (2)	1	1 (1)	–	3 (4)			
**personal care & household**	**11**	**A**	0	3	7	5	2	5	27	81	**91**
		**E**	1	3	0	4	1	4			
**polymer additives**	**10**	**A**	0	2	4 (1)	3	4	4 (1)	50	70 (+10)	**80**
		**E**	0	1 (1)	0	2	1	3 (1)			
**pharmaceuticals**	**16**	**A**	2	2	3 (2)	10	3 (2)	5	19 (+13)	37	**50**
		**E**	1	1	1	2 (2)	0 (2)	1			
**pesticides/biocides**	**30**	**A**	6	7	9 (1)	13 (2)	4	9 (1)	16	40 (+3)	**43**
		**E**	2 (1)	5 (2)	0	7	1	3 (1)			
**food, beverage & stimulants**	**4**	**A**	1	1	2 (1)	1	0	0	0	0	**0**
		**E**	0 (1)	0 (1)	0	1	0	0			
**other**	**10**	**A**	3	2	4	3	3	1	50	30	**60**
		**E**	0	3	1	3	2	2			
**∑**	**100**	**A**	**14 (1)**	**25 (2)**	**37 (10)**	**36 (2)**	**16 (2)**	**30 (7)**	**21 (+2)**	**46 (+7)**	**54**
		**E**	**6 (5)**	**16 (7)**	**3 (0)**	**20 (4)**	**5 (2)**	**16 (7)**			

## Data Availability

Micropollutant concentrations, chemical identity and property data as well as other metadata is provided in the Supplementary Materials.

## Appendix A. Supplementary data

Supplementary data to this article can be found online at https://doi.org/10.1016/j.envpol.2025.126808.
