# Supplementary material for: Current emission vs. legacy organic pollutants: Assessing the extent to which the eco-exposome of caged fish reflects external exposure
Source: Environ Pollut. Author manuscript; Available in PMC 2026 Jul 10. (PMC12915642; doi:10.1016/j.envpol.2025.126808)
Supplement: Supplement2 [file NIHMS2109224-supplement-Supplement2.docx]

# Current emission vs. legacy organic pollutants: Assessing the extent to which the eco exposome of caged fish reflects external exposure – Supportive Material A

Janek P. Dann^1,2^, Gerald T. Ankley^3^, Brett R. Blackwell^3^, Beate I. Escher^1,4^, Annika Jahnke^1,5^, Kathleen M. Jensen^3^, Correne Jenson^3^, Martin Krauss^1^, Stefan Scholz^1^, Theo Wernicke^6^, Werner Brack^1,2^

^1^Helmholtz Centre for Environmental Research GmbH – UFZ, Permoserstraße 15, 04318 Leipzig, Germany

^2^Institute of Ecology, Diversity and Evolution, Goethe University Frankfurt, 60438 Frankfurt am Main, Germany

^3^United States Environmental Protection Agency, Office of Research and Development, Great Lakes Toxicology and Ecology Division (GLTED), 6201 Congdon Blvd, Duluth, MN 55804, USA

^4^Environmental Toxicology, Department of Geosciences, Eberhard Karls University Tübingen, 72076 Tübingen, Germany

^5^Institute for Environmental Research, RWTH Aachen University, 52074 Aachen, Germany

^6^Umweltbundesamt, UBA, Wörlitzer Platz 1, 06844 Dessau-Roßlau

E-mail contact: martin.krauss@ufz.de

# Table of content SM-A (this document)

[Current emission vs. legacy organic pollutants: Assessing the extent to which the eco exposome of caged fish reflects external exposure – Supportive Material A 1](#_Toc198671826)

[S1. Site location in the St. Louis Bay close to Duluth (MN, USA) 7](#_Toc198671827)

[S2. Chemicals 7](#_Toc198671828)

[S3. Sampling 8](#_Toc198671829)

[S3.1 Caged fish deployment and laboratory studies 8](#_Toc198671830)

[S3.2 Water and sediment sampling 8](#_Toc198671831)

[S3.3 Homogenization of FHM 9](#_Toc198671832)

[S4. Extraction 9](#_Toc198671833)

[S4.1 QuEChERS extraction of FHM and fish food 9](#_Toc198671834)

[S4.2 Extraction of water samples 10](#_Toc198671835)

[S4.3 Extraction of sediment samples 11](#_Toc198671836)

[S5. Instrument settings and data evaluation 12](#_Toc198671837)

[S5.1 Instrumental Analysis – LC-HRMS 12](#_Toc198671838)

[S5.2 Instrumental Analysis – GC-HRMS 12](#_Toc198671839)

[S5.3 Data evaluation 13](#_Toc198671840)

[S5.4 Calibration and quantification 14](#_Toc198671841)

[S6. Time course of concentrations in the water samples 15](#_Toc198671842)

[S7. Lipid content of FHM in relation to detected concentrations 16](#_Toc198671843)

[S8 Statistical Analysis 17](#_Toc198671844)

[S8.1 Principal Component Analysis 17](#_Toc198671845)

[S8.1.1 Fathead Minnows 17](#_Toc198671846)

[S8.1.2 Water 18](#_Toc198671847)

[S8.1.3 Sediment 19](#_Toc198671848)

[S8.1.4 FHM, water, and sediment 20](#_Toc198671849)

[S8.2 Correlation Analysis 21](#_Toc198671850)

[S8.3 Outcome statistical analysis 24](#_Toc198671851)

[S9. Distribution and co-occurrence of compounds among the samples 25](#_Toc198671852)

[S10. Co-occurrence of chemicals in water, sediment, and FHM 30](#_Toc198671853)

[S11. Time- and site-dependent comparison of 2-d FHM, 21-d FHM, water and sediment concentrations 34](#_Toc198671854)

[S11.1 Substances only detected in 2-d FHM 38](#_Toc198671855)

[S11.2 Substances only detected in 21-d FHM 38](#_Toc198671856)

[S11.3 Substances detected in 2-d and 21-d FHM 39](#_Toc198671857)

[S11.4 Substances detected in water and sediment, not in FHM 42](#_Toc198671858)

[S11.5 Substances only detected in water 42](#_Toc198671859)

[S11.6 Substances only detected in sediment 43](#_Toc198671860)

[S11.7 FHM controls 43](#_Toc198671861)

[S12. Comparison of 2-d FHM, 21-d FHM, water and sediment concentrations 44](#_Toc198671862)

[S13. BCF and BSAF calculation 45](#_Toc198671863)

[S14. Comparing predicted and experimental *c*_FHM_ 46](#_Toc198671864)

[S15. Comparing predicted and experimental BCF and BSAF with *c*_FHM_ / c_w_^-1^ and *c*_FHM_ / c_sed_^-1^ 58](#_Toc198671865)

[S15.1 Distribution between water and sediment 58](#_Toc198671866)

[S15.2 Concentration ratios between fish and water as compared to predicted BCF_eq_ values 61](#_Toc198671867)

[S15.3 Concentration ratios between fish and sediment as compared to predicted BSAF values 62](#_Toc198671868)

[S16 Comparison contamination patterns in FHM with findings from the literature 63](#_Toc198671869)

[References 64](#_Toc198671870)

# The following Tables are in a separate Excel file (SM part B):

S1 Site description

S2 Substances

S2a target substances and additional information

S2b list of used internal standards

S2c substances and additional information

S3 Water data

S3a measured concentrations of the different time points and the site averages plus standard-deviations

S3b contains average plus standard-deviations of concentrations at the different time point

S4 Sediment data

S4a sediment wet weights

S4b sediment dry weights

S4c total organic carbon (TOC) of the sediment

S4d concentration in sediment from all sites; application of stability factor

S4e reduced dataset of the concentrations with average values and standard deviation

S5 Fathead Minnow data

S5a sampling data of caged FHM; 2-d FHM

S5b sampling data of caged FHM; 21-d FHM

S5c wet weights

S5d wet weight normalized concentrations (ng/g_ww_) for each clean-up method

S5e aggregated dataset using the concentrations of the method with better recovery

S5f MAX values of *c*_FHM_ at each site

S5f lipid content of FHM samples

S5g FHM concentrations normalized to lipid content (see S5f); PSA and C18 combined depending on recoveries and MDLs

S5h MAX values of *c*_FHM_ (ww) at each site

S6 Fish food data

S6a wet weight and lipid content of brine shrimp and trout chow

S6b wet weight-normalized concentrations

S7 output data from principal component analysis (PCA)

S7a: water (all)

S7b water (mean values)

S7c sediment (all)

S7d sediment (mean values)

S7e FHM (female and male separately)

S7f FHM (max. values)

S7g water vs. FHM

S7h sediment vs. FHM

S7i water vs. sediment

S7j FHM vs. water vs. sediment

S8 Literature data for *K*_OC_ data from Niu et al. and EPISuite

S9 Data used for Venn diagram and violin plot

S10 Data used for systematic comparison of occurrences in FHM, water, and sediment

S9a Number of pattern-detects (A, E); systematic comparison of the occurrence of target substances

S9b Detected compound classes

S9c Number of overlaps

S9d Agreement occurrence in FHM

S9e Number of pattern-detects

S11 Calculated BCF and BSAF

S12 Values of the predicted (expected) concentrations in FHM derived from the concentrations in water, sediment, and the predicted BCF and BSAF values

# Figures in SM-A

**Figure S1:** Site locations in the St. Louis Bay close to Duluth (MN, USA).

**Figure S2.1a-b:** Principal component analysis (PCA) of FHM maximum values. a) Male and female FHMs separately; b) PCA using the combined value of the maximum of male and female FHMs. Blue: 2-d FHMs; red: 21-d FHMs.

**Figure S*2*.2a-b:** PCA for water. a) time points separately; one outlier is marked in red color; b) combined using the mean value of the different time points for each site.

**Figure S2.3a-b:** PCA of sediment values. a) all sites separately: FHM sampling sites and additional “Zone” sites; b) with aggregated values for Ponds and Zones.

**Figure S2.4a-d:** PCAs from combined datasets a) for water, sediment, and FHM; b) for water and sediment; c) for FHM and water; d) for FHM and sediment. Colors: blue: water, orange: sediment, light green: 2-d FHM, dark green: 21-d FHM.

**Figure S3.1:** Correlation matrix of the log-transformed and standardized concentration data of 2-d FHM, 21-d FHM, water, and sediment.

**Figure S4.1-4.5:** UpSet plots: **S4.1**) water; **S4.2**) sediment; **S4.3**) 2-d FHM; **S4.4**) 21-d FHM; **S4.5**) overlap between water, sediment, FHM 2-d and FHM 21-d.

**Figure S5a-c:** a) Venn diagram of the co-occurrence of detected substances in water, sediment, and FHM and b) occurrence relative to the log *K*_ow_ of the neutral species. Bin width setting concerning the grouping of the dots for the log *K*_ow_ is 1/30 of the range of the data. The color code in a) is also used for the violin plot b). In c), the color represents the compound class of each substance.

**Figure S6a-c:** site- and time-dependent concentrations of substances only found in 2-d FHM, only in 21-d FHM, or in both 2-d FHM and 21-d FHM compared to their concentrations in water and sediment.

**Figure S7a-i:** Expected log *c*_FHM_, at Creek, Pond S, Pond N, and WWTP derived from BCF and *c*_w_ / BSAF and *c_sed_*: a) compounds in high concentrations; b) *personal care and household*; c) *pharmaceuticals*; d) *POPs*; e) *polymer additives*; f) *PAHs*; g) *pesticides/biocides*; h) *food, beverage & stimulants*; i) *other*.

**Figure S8a-c:** Comparison experimental (fractions of c_FHM_, c_sed_, c_w_) with predicted values (log *K*_OC_, log BCF, log BSAF), based on substances only occurring in 21‑d FHM above the FHM control threshold. a) log c_sed_ × c_w_^-1^ plotted against log *K*_OC_; b) log c_FHM_ × c_w_^-1^ plotted against log BCF; c) log c_FHM_ × c_sed_^-1^ plotted against log BSAF

**Figure S9a-b:** Comparison experimental (fractions of c_FHM_, c_sed_, c_w_) with predicted values (log *K*_OC_, log BCF), based on substances above the FHM control threshold, both 2‑d and 21‑d FHM. a) log c_sed_ × c_w_^-1^ plotted against log *K*_OC_; b) log c_FHM_ × c_w_^-1^ plotted against log BCF; c) log c_FHM_ × c_sed_^-1^ plotted against log BSAF

Tables in SM-A:

**Table S1**: a) MZmine 2.38 settings for LC-HRMS data. b) MZmine 2.39 settings for GC-HRMS data.

**Table S2.** Overlaps of detects and non-detects of FHM with the ones in either water or sediment across all sites, the former distinguished by the two timepoints 2-d and 21-d in case of FHM and water.

# S1. Site location in the St. Louis Bay close to Duluth (MN, USA)

In Figure S1, the sampling locations are displayed (extracted from Google Maps in December 2018). For more information on exact locations, sampling information and water quality characteristics please also consider also SM-B, Table S1.

**
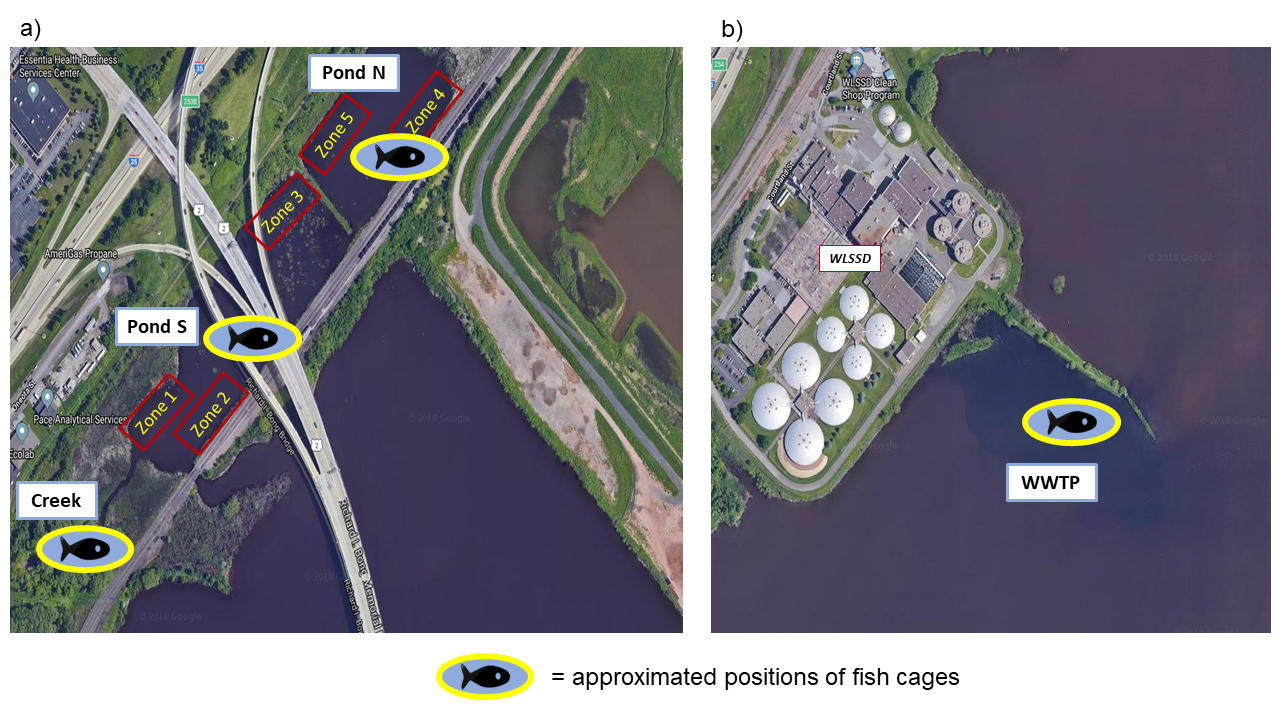
**

**Figure S1.** Site locations in the St. Louis Bay close to Duluth (MN, USA). a) Shoppers Creek and Erie Pier Ponds North and South (“Creek”, “Pond N”, “Pond S”) FHM cage locations and additional “zones”, where sediment was sampled. b) Western Lake Superior Sanitary District (WLSSD; wastewater treatment plant; “WWTP”) site.

# S2. Chemicals

Methanol, ethyl acetate, acetonitrile, isopropanol and acetone were of LC-MS grade and obtained from Honeywell, LC-MS grade water was from Fisher Scientific. Formic acid, MgSO_4_, NaCl, NaOH, NaN_3_; Silicone (Dowsil DC 1-2577, obtained from Dow Chemical Company) were at least of analytical grade quality.

For calibration, mixed standard solutions of the target analytes at 1 µg/mL were prepared for LC-HRMS (liquid chromatography-high resolution mass spectrometry) analysis in methanol (359 compounds) and for GC (gas chromatography)-HRMS analysis in ethyl acetate (98 compounds), both including one compound (piperonyl butoxide) present in both mixes. A list of all analyzed substances along with additional information can be found in SM-B, Table S2a. Internal standard mixtures containing isotope-labeled compounds were prepared the same way for LC-HRMS (40 compounds) and GC-HRMS (22 compounds; SM-B, Table S2b), respectively. 189 analytes were not extractable from the FHM with the applied method but showed acceptable recoveries in water and/or sediment samples, some of which were also detected in rather high concentrations. These compounds were not included in further data evaluation but can be found in SM-B, Table S2c.

# S3. Sampling

## S3.1 Caged fish deployment and laboratory studies

All fish were maintained in filtered, UV-treated Lake Superior water at 25°C and fed thawed adult brine shrimp and crumbled starter feed *ad libitum* until used for experimentation. Four adult male and four female FHMs (approximately 6-7 months old) were placed in each of the five deployed cages at every site in September 2018. The cages were suspended at a depth of 0.9-1.1 m and the deployed fish received no supplemental food. Water temperature was monitored at each site using automatic data loggers and, concurrent with deployment and retrieval of the caged fish and water samples, measurements of dissolved oxygen, pH, alkalinity, hardness, conductivity and nutrients were obtained at each site (SM-B, Table S1). Laboratory controls consisted of four male and four female FHMs that were held in each of five replicate 20 L tanks at the GLTED facility. These control fish were exposed to a continuous flow of filtered, UV-treated Lake Superior water (45 mL/min, 6.5 volume additions per day), and fed thawed adult frozen brine shrimp *ad libitum* daily. Water quality characteristics for the laboratory controls are also provided in the SM (SM-B, Table S1). Detailed methodology is described elsewhere.^1, 2^

When FHMs were retrieved following 2 d and 21-d of exposure, they were placed into buckets of site water and transported to GLTED for processing. The fish were processed one cage at a time, generally within 2 h of retrieval from the field. They were anesthetized with buffered tricaine methanesulfonate (MS-222; Finquel; Argent, Redmond, WA), and wet weights were recorded. Laboratory control fish were processed concurrently; all samples were wrapped in solvent-rinsed foil and stored at -20°C. Sample sizes for this work ranged between 13-16 and 12-16 fish for the 2-d and 21-d exposures, respectively. Perished fish were included if the tissue was not visually compromised.

## S3.2 Water and sediment sampling

During the 21-d deployment, weekly composite samples were collected and processed independently. To achieve adequate sample volume during the 48-hr exposure, two autosamplers were joined with a tee line that supplied water from each sampler to one collection bottle. Following retrieval, two liters of these composite sample were transferred into precleaned amber bottles, placed on ice and transported to GLTED for processing. and processed as described below.

Sediment was collected within 3.5 m from each caged fish deployment site approximately 2 weeks after fish and water samples were removed. To obtain a representative sediment sample, 3-5 grab sample aliquots were combined at WWTP and Ponds, while from Shoppers Creek 20 grab sample aliquots had to be combined due to the limited sediment depth to yield a 4 L composite sample. Details for the sediment extraction are provided below.

## S3.3 Homogenization of FHM

The frozen fish were sliced into small pieces using razor blades and cooled with liquid nitrogen for 2-3 min. The frozen pieces were then transferred into stainless steel grinding jars, a metal ball was added, the container was closed and shaken at 30 Hz for 3 min (containers, ball and shaker were from Retsch: “cryo mill”). About 4-5 g of the frozen fish homogenate was then directly transferred to 15 ml polypropylene tubes for extraction. Depending on their weight, four to six fish were pooled to reach a mass of ca. 4 g per extraction, which in some cases entailed pooling individuals from different cages, but from the same site and sex for any given sample. If the amount of sample was sufficient, replicates containing fish from the same cage(s), site, sex, and time point were generated.

# S4. Extraction

## S4.1 QuEChERS extraction of FHM and fish food

*FHM.* For extraction and clean-up of the fish homogenate a modified QuEChERS method^47^ was used. About 4 g of homogenate were mixed with 4 mL of LC-MS grade water, 4 mL of acetonitrile and vortexed for 1 min. Before and after adding 0.48 g of NaCl and 1.92 g of MgSO_4_ to induce phase separation, the tube was centrifuged at 4000 × g for 5 min. The supernatant acetonitrile phase was transferred into 7 mL brown glass vials, evaporated to dryness in a nitrogen stream and stored at ‑20°C until shipment to Leipzig, Germany. In Germany, samples were re-dissolved in 6 mL of acetonitrile. Two separate clean-up strategies using dispersive SPE were employed. One fraction of the sample was mixed with 30 mg of primary-secondary amine (PSA; Agilent) and 240 mg of MgSO_4_ to improve matrix removal, resulting, however, in low recoveries for several (mainly negatively charged) compounds. For the second fraction, an alternative clean-up sorbent 30 mg of C_18_-modified silica gel (C18; Agilent) and 240 mg of MgSO_4_ was used, which removed less matrix constituents, but showed better recoveries for most of the compounds lost in the PSA clean-up. The clean-up was achieved by adding the respective mass of sorbent and salt to a 1.5 mL aliquot of the extract, vortexing for 1 min and centrifuging at 4500 × g for 5 min. The supernatant was transferred into a 4 mL brown glass vial and the sorbent was re-extracted with 1 mL of acetonitrile. After centrifugation, both extracts were combined, evaporated to dryness and re-dissolved in 400 µL of methanol, from which 100 µL were taken for LC-HRMS analysis, 100 µL were evaporated and dissolved in 100 µL of ethyl acetate for GC-HRMS analysis, and the remaining 200 µL were stored at -20°C. For every sorbent, one blank was processed in the same way as the samples, but without any matrix (only ACN and water). Prior to analysis, 100 µL of the samples as well as the extraction blanks were spiked with 10 µL of internal standard mix containing 38 isotope-labelled compounds at 1 ug/mL (see SM-B, Table S2b). A method-matched calibration approach was used and ten standard solutions were prepared corresponding to levels between 1 and 1000 ng/mL in the vial for analysis. For method-matched calibration only water was used, without addition of fish homogenate.

*Fish food.* Frozen adult brine shrimp (*Artemia congelata*, San Francisco Bay Brand, Newark, CA) and crumbled starter feed (“trout chow”, Skretting USA, Tooele, UT) comprised the standard diet that the FHM were fed in the culture unit until they were utilized for the study. Of each food type three replicates were used for analysis. Only one batch of the brine shrimp samples were the same batch as used for the fish in the study, the second batch of brine shrimp and the crumbled starter feed were from the following year, but commercial vendors and brands used were the same. The brine shrimp and crumbled starter feed was extracted with the same method as the FHM.

## S4.2 Extraction of water samples

Water samples were subjected to solid-phase extraction (SPE) after filtration using glass fiber filters (Whatman) and acidification to pH 3. Oasis HLB cartridges (500 mg; Waters) were conditioned using 10 mL of ethyl acetate, methanol, and water acidified to pH 3, respectively. Two liters of each sample were subsequently extracted, and a blank sample was prepared using 2 L of LC-MS grade water. After drying in a nitrogen evaporator, cartridges were shipped to Leipzig, where they were eluted using 10 mL of ethyl acetate, followed by 10 mL methanol. The combined extract was evaporated to near dryness in a nitrogen stream and dissolved in 2 mL methanol. Prior to analysis, 100 µL of the extract were transferred into a 2 mL autosampler vial with glass insert, and 10 µL of the LC-HRMS internal standard solution (nominal concentration 1 µg/mL in methanol), 60 µL of water and 30 µL of methanol were added. Method-matched calibration standards were prepared from spiked 1 L aliquots of water from a pristine stream (Wormsgraben, upper Harz mountains, Germany) at ten calibration levels between 1 and 1,000 ng/L using the same procedure as for the samples. By mistake two samples of the WWTP water SPE samples were combined into one sample, but the proportion was kept in a way that the substance load of the resulting sample remained comparable to the other samples.

## S4.3 Extraction of sediment samples

For the passive equilibrium sampling of the sediment, 120 mL glass jars were coated with a layer of DC1-2577 silicone at 4 µm, 8 µm, 16 µm thickness using the method of Jahnke et al.^3, 4^ For each sediment sample, one wet aliquot of 80-100 g was weighed into one jar per coating thickness. If needed, up to 10 mL of water was added to achieve homogenous consistency. The pH was adjusted with NaOH to 7 if necessary and 0.1 g NaN_3_ was added. The jars were then continuously rolled horizontally at approximately 40 rpm in the dark at room temperature over 3 weeks. Two blank samples were prepared with a PDMS layer of 16 µm thickness using LC-MS grade water only. Details on water content and exact weights of the samples can be found in SM-B, Table S4a.

After 3 weeks, the sediment was removed, the jars thoroughly rinsed with water, wiped dry with lint-free tissue, and kept frozen at -20°C before being shipped to Leipzig, Germany. Dry weights can be found in SM-B, Table S4b. The chemicals in the PDMS coating were extracted with two aliquots of 2 mL ethyl acetate, with internal standard mix added to the first aliquot, while rolling for 30 min each, and the resultant extracts were evaporated to 5 µL and taken up in 50 µL of ethyl acetate. Prior to analysis, an aliquot was transferred into a 2 mL autosampler vial. Ten calibration standards between 1 and 1,000 µg/mL were prepared in ethyl acetate (for GC-HRMS) or methanol (for LC-HRMS). Several samples were taken at different locations at the Ponds, which were treated as replicates for the respective site (Section S1). The average concentrations of zones 1, 2, 3 and Pond S were calculated and, in this way, combined to create sample “Pond S” and concentrations of zones 4, 5 and Pond N were combined to create sample “Pond N”.

# S5. Instrument settings and data evaluation

## S5.1 Instrumental Analysis – LC-HRMS

LC-HRMS analysis was conducted using a Thermo Ultimate 3000 LC system (consisting of a ternary pump, autosampler and column oven) coupled to a quadrupole-orbitrap mass spectrometer (Thermo QExactive Plus). A 5 µL aliquot of each sample was injected for reversed-phase separation using a C18 column with a methanol/water gradient, and the compounds were ionized via a heated electrospray ionization (ESI) source. A Kinetex C18 EVO column (50 × 2.1 mm, 2.6 *µ*m particle size) was used for LC separation with a gradient elution with 0.1% of formic acid (eluent A) and methanol containing 0.1% of formic acid (eluent B) at a flow rate of 300 *µ*L/min. After 1 min of 5% B, the fraction of B was linearly increased to 100% within 12 min and 100% B were kept for 11 min. The eluent flow was diverted to waste and the column was rinsed for 2 min using a mixture of isopropanol + acetone 50:50 / eluentB / eluent A (85% / 10% / 5%) to flush the column and remove hydrophobic matrix constituents. Subsequently, the column was re-equilibrated to initial conditions for 5.7 min. The injection volume was 5 *µ*L and the column was operated at 40°C. Separate runs were conducted on a Thermo QExactive Plus instrument in positive and negative ion mode The heated ESI source and the transfer capillary were both operated at 300°C, the spray voltage was 3.8 *k*V (pos mode) or 3.5 *k*V (neg. mode), the sheath gas flow rate was 45 a.u. and the auxiliary gas flow rate 1 a.u. combining a MS1 full scan experiment (100-1500 m/z) at a nominal resolving power of 70,000 (referenced to m/z 200) and MS^2^ data-independent acquisition in 12 isolation windows at a resolving power of 35,000. For the latter, we acquired the data using broad isolation windows of about 50 mu (i.e., m/z ranges 97-147, 144-194, 191-241, 238-288, 285-335, 332-382, 379-429, 426-476) and 260 mu (i.e., m/z ranges 473-733, 729-989, 985-1245, 1241-1501), respectively.

## S5.2 Instrumental Analysis – GC-HRMS

For GC-HRMS analysis, a QExactive GC system (Thermo) was used, consisting of a Gerstel MPS autosampler equipped with a TDU3 thermodesorption unit, a Trace 1300 GC equipped with a Gerstel Cold injection system and a QExactive HF mass spectrometer. Extract aliquots of 2 µL were injected in splitless mode into thermodesorption tubes equipped with glass inserts, which can be considered as single use liners. The TDU3 unit was kept at 80°C for 4 minutes and subsequently heated at 720°C/min to 300°C to transfer analytes into the cold injection system of the GC, which was kept at 10°C. After analyte transfer completion at 5 minutes, the GC injector was heated at 12°C/s to 300°C, held for 10 min. For chromatographic separation a DB-5MS capillary column (30 m × 250 µm 0,25 µm; Agilent) was used with helium as carrier gas at a constant flow rate of 1.2 mL/min. The oven was kept at 60°C for 1 minute, heated at 30°C/min to 150°C, followed by 6°C/min to 186°C and 4°C/min to 300°C, which was held for 11.5 minutes. The temperature of transfer line was 250°C. Electron ionization with an ionization energy of 70 eV and a source temperature of 200°C was used. Data was acquired in full scan mode at m/z 60-810 at a nominal resolving power of 60,000 (referenced to m/z 200).

## S5.3 Data evaluation

*MZmine workflows and settings*. The settings for MZmine 2.38 used for LC-HRMS data are displayed in Table S1a, the MZmine 2.39 settings used for GC-HRMS data are shown in Table S1b.

**Table S1a:** MZmine 2.38 settings for LC-HRMS data.

| **Step** | **Parameter** | **Setting** |
| --- | --- | --- |
| Mass detection | Mass detector | Centroid |
|  | Noise level | 5000 |
| ADAP chromatogram abuilding | Min group size of # of scans | 8 |
|  | Group intensity threshold | 10000 |
|  | Min highest intensity | 5000 |
|  | m/z tolerance | 0.001 m/z or 7 ppm |
| Smoothing | Filter width | 7 |
| Chromatogram deconvolution | Algorithm | Local minimum search |
|  | Chromatographic threshold | 60% |
|  | Search minimum in RT range | 0.10 min |
|  | Minimum relative height | 30% |
|  | Minimum absolute height | 50000 |
|  | Min ration of peak top/edge | 2.3 |
|  | Peak duration range | 0.1 – 5 min |
| Join aligner | m/z tolerance | 0.001 m/z or 7 ppm |
|  | Weight for m/z | 70 |
|  | Retention time tolerance | 0.3 (absolute) min |
|  | Weight for RT | 30 |
| Custom database search | m/z tolerance | 0.001 m/z or 7 ppm |
|  | Retention time tolerance | 0.4 (absolute) min |
| Gap filling | Intensity tolerance | 30% |
|  | m/z tolerance | 0.001 m/z or 7 ppm |
|  | Retention time tolerance | 0.15 (absolute) min |
|  | RT correction | yes |

**Table S1b:** MZmine 2.39 settings for GC-HRMS data.

| **Step** | **Parameter** | **Setting** |
| --- | --- | --- |
| Mass detection | Mass detector | Centroid |
|  | Noise level | 1000 |
| ADAP chromatogram building | Min group size of # of scans | 12 |
|  | Group intensity threshold | 1000 |
|  | Min highest intensity | 5000 |
|  | m/z tolerance | 0.001 m/z or 5 ppm |
| Smoothing | Filter width | 7 |
| Chromatogram deconvolution | Algorithm | Local minimum search |
|  | Chromatographic threshold | 10% |
|  | Search minimum in RT range | 0.05 min |
|  | Minimum relative height | 5% |
|  | Minimum absolute height | 30000 |
|  | Min ration of peak top/edge | 2.3 |
|  | Peak duration range | 0.02 – 2.5 min |
| Join aligner | m/z tolerance | 0.001 m/z or 5 ppm |
|  | Weight for m/z | 70 |
|  | Retention time tolerance | 0.15 (absolute) min |
|  | Weight for RT | 30 |
| Custom database search | m/z tolerance | 0.001 m/z or 5 ppm |
|  | Retention time tolerance | 0.15 (absolute) min |
| Gap filling | Intensity tolerance | 40% |
|  | m/z tolerance | 0.001 m/z or 7 ppm |
|  | Retention time tolerance | 0.15 (absolute) min |
|  | RT correction | yes |

## S5.4 Calibration and quantification

Internal standard calibration was used to compensate for differences in matrix effects among calibration standards and samples; to this end, the internal standard with the closest retention time was automatically assigned for each analyte.

Compounds with very broad peaks, shifting retention times and/or high background noise were quantified using the TraceFinder 4.1 software (Thermo Scientific), which was also used to confirm the correct annotation of compounds in MZmine using MS^2^ fragment ions. Method detection limits (MDLs) were determined using an approach based on a US EPA protocol^5^ with replicate injection of the respective method-matched calibration series. For every compound measured with LC-HRMS the recovery achieved with the two extraction sorbents PSA and C18 was calculated and compared. Only the values with the method showing the best recovery were used for further interpretation (see SM-B Table S5d-e). In case of similar recovery, the one with lower MDL were selected.

# S6. Time course of concentrations in the water samples

To assess whether the concentrations in the water extracts showed any temporal trends or could be considered as stable during the 21-day exposure of the caged fish, the concentrations of each compound were plotted against the sampling time for each site at day 2, 7, 14 and 21) and the slopes $m_{\bar{c}}$ were calculated. As the low number of samples did not allow for a reliable statistical testing, an operational boundary was defined as follows:

Derived from the average aqueous concentration $\bar{c}_{w, site}$ over time of each compound at every site, double and half of the average water concentration, $2\bar{c}_{w, site}$ and $\bar{c}_{w, site}/2$, was calculated. These values were used to generate fictive positive and negative “slope thresholds” $m_{\bar{c}_{w, site}/2}$ and $m_{2\bar{c}_{w, site}}$ which were then compared to the actual slope $m_{c_{w, site}}$ generated by the discrete datapoints over time. Using this calculation, the concentrations were roughly stable ($m_{\bar{c}_{w}/2}\leq m_{c_{w, site}}\leq m_{2\bar{c}_{w}}$) for 59 (Pond N) / 67 (Pond S) / 66 (Creek) substances, and showed a decrease tendency ($m_{c_{w, site}}<m_{\bar{c}_{w, site}/2}$) over time for 31 / 12 / 12 substances. An increasing tendency ($m_{c_{w,site}}>m_{2\bar{c}_{w}}$) was only found at Pond S (2 chemicals) and Creek (17 chemicals). Prominent examples for temporal variation were 2,4‑dichlorophenoxyacetic acid with maximum peaks at the second time point *t*_2-d_ (9_13_2018) at Pond N and Pond S; TMDD and Tramadol with a maximum at the first time point and following increasing concentrations at Creek and Pond N and triethyl phosphate at Creek**,** Pond S and Pond N; 1,3-diphenylguanidine, 5-methyl-1H-benzotriazole, and hexa(methoxymethyl)melamine showed increasing concentrations with a maximum at the last time point at Creek.

Examples for compounds with high concentrations (≥ 1µg/L at least at one time point) at the different sites were 1H-benzotriazole, 2‑benzothiazolesulfonic acid, 2-(methylthio)­benzothiazole, 5-methyl-1H-benzotriazole, benzothiazole, caffeine, cotinine, DEET, ISO E Super, naproxen, tramadol, triethyl citrate, tributyl phosphate, and *o*-toluidine at the WWTP; 2-benzothiazolesulfonic acid, 5-methyl-1H-benzotriazole, hexa(methoxy­methyl)­melamine, TMDD, tramadol, and tris(1-chloro-2-propyl)phosphate at Creek; 2,4-dichloro­phenoxyacetic acid and TMDD at Pond N. At Pond S, no concentration exceeded 1 µg/L.

Further notice regarding water samples: by mistake two samples of the WWTP water SPE samples were combined to one sample, but the proportion was kept.

# S7. Lipid content of FHM in relation to detected concentrations

**Short protocol:** The wet sample were weighed into collection vials (heated before for 1 h at 110°C). Distilled water (MilliQ) was added according to water content. Afterwards, solvents (1.3 mL of cyclohexane and 1 mL of isopropanol) were added. The sample was vortexed for 1 min and centrifuged at 4000 rpm for 5 min. The upper organic layer was transferred to a collection tube. Then, the following steps were repeated three times: add 1.16 mL cyclohexane and 0.175 mL 2-propanol, vortex, centrifuge, transfer; and after every time the glass pipette was rinsed. The transferred solvents were evaporated in a stream of nitrogen and the collection vials were heated for 1 h at 110°C. Finally, the collection vials were cooled down, put in a desiccator overnight, and the collection vials were weighed again.

The traditional way of reporting internal concentration in fish is on a lipid basis (ng/g_lipid_) but we reported concentrations based on the wet weight (ng/g_ww_) because also ionizable and hydrophilic chemicals were analyzed in fish. However, for lipophilic chemicals the fat is the phase where the chemicals will end up and because there were sex-specific and individual differences in lipid content, we also evaluated the role of the lipid content for the internal concentrations.

The lipid content of the FHM differed by sex and site with the overall mean values (shown in % of wet weight ± st.-dev.) being 2.74 ± 0.75 (female) and 0.92 ± 0.28 (male). Regarding the different sampling sites and time points, there were two sites (Creek and WWTP) where the average lipid content of the female FHMs increased from 2-d to 21-d (Creek: + 0.58%, WWTP: + 1.26%), while it decreased in the male samples (Creek: ‑ 0.41%, WWTP: ‑ 0.01%). At Pond S, the average lipid content decreased in both male (‑ 0.63%) and female (‑ 1.58%) FHMs, while at Pond N, due to a missing data point only the difference for the male FHMs could be determined (‑ 0.18%), but nonetheless the female FHMs from this site showed the lowest lipid content (1.87%) in comparison to the females from all other sites and time points. The control samples showed an increasing average lipid content over time in both male (+ 0.36%) and female (+ 1.68%) FHMs. For further details see SM Table S5f-g (FHM) in SM-B and for data of the fish food see SM Table S6a in SM-B.

Although it was established above that the water concentrations did not show a trend over the three weeks of fish exposure, the accumulated concentrations in FHMs increased from 2 to 21 d at the Creek and Pond S but decreased at Pond N. Increasing concentrations would be expected with ongoing uptake through bioconcentration, which should not have reached steady state within 2 days for the more hydrophobic substances. The decreasing concentrations at Pond N could also be related to the depuration of existing contaminants and overall lower concentration of chemicals in the water phase for uptake and an additional factor could be the lower lipid content in the FHM from that site and the resulting lower affinity of more hydrophobic contaminants, which lead to concentrations close to or below the set threshold of two times the maximum concentration of controls.

# S8 Statistical Analysis

## S8.1 Principal Component Analysis

A principal component analysis (PCA) was applied to different combinations of subsets of log-transformed and standardized data for FHM, water, and sediment. The results can be found in SM-B, Table S7a-j.

### S8.1.1 Fathead Minnows

Two performed PCAs of the FHM data are presented in Fig. 4.1. Both, the more detailed PCA distinguishing between male and female FHMs (Fig. 4.1 a) and the less detailed PCA without this differentiation (Fig. 4.1 b) showed a clear distinction between the different timepoints 2-d and 21-d. In Fig. 4.1 a) which seems to be well represented by Dim 2, explaining 15.4 of the data distribution. The 2-d FHMs all have positive Dim 2 values, whereas 21-d FHMs have negative ones; in Fig. 4.1 b): it’s represented by Dim 1, explaining 22.7% of the variation – here again 2-d FHMs have positive values and 21-d FHM negative ones. The corresponding other dimensions can be explained by the different sites in both plots, with the WWTP showing the most negative, the Creek the most positive values, and the Ponds’ values in between.


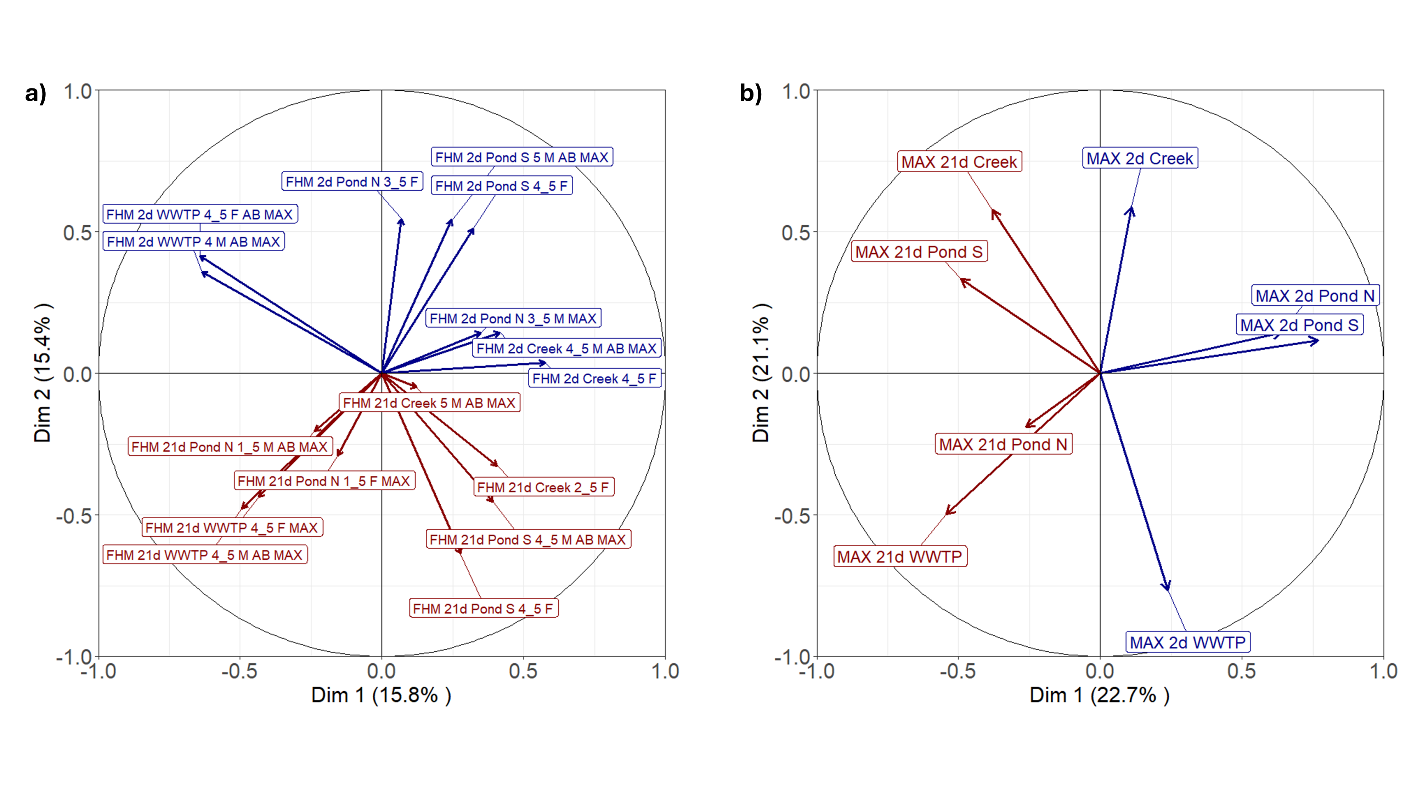


**Figure S2.1a-b:** PCA of FHM maximum values. a) Male and female FHMs separately; b) PCA using the combined value of the maximum of male and female FHMs. Blue: 2-d FHMs; red: 21-d FHMs.

### S8.1.2 Water

The PCAs with the water data are shown in Fig. S5.2. The first two dimensions can explain 23.3% in case of Dim 1 and 19.7% in case of Dim 2. The analysis of the individual samples from the different time points (Fig. S5.2a) shows that the different time points correlate stronger with each other than among different sites. The ponds N and S were quite similar to each other indicated by the proximity of the vectors, mostly in the negative range of both Dim 1 (Pond N: -0.57 to -0.29; Pond S: -0.38 to 0.14) and Dim 2 (Pond N: -0.51 to ‑0.31; Pond S: -0.57 to 0.00). The WWTP samples were the most different from the other samples with a high value of 0.91-0.92 for Dim 1 and a small (positive) value of 0.08-0.13 for Dim 2. In contrast, the Creek samples show negative values for Dim 1 (-0.47 to ‑0.17) and positive values for Dim 2 (0.46 to 0.65). As Dim 1 explains a higher percentage of the data than Dim 2, Creek is more similar to the Ponds than to WWTP, but still significantly different from the Ponds. Interestingly, the PCA of the mean values of each site (Fig. S5.2b) also show a large difference, especially regarding Dim 1, between WWTP (Dim 1: ‑0.99; Dim 2: ‑0.11), and the other sites, but here Creek and Pond N are very similar in both Dim 1 (Creek: 0.63; Pond N: 0.74) and Dim 2 (Creek: ‑0.20; Pond N: ‑0.17), yet Pond S (Dim 1: 0.15; Dim 2: 0.99) seems to be more different as indicated by Dim 2. One factor might be the outlier of the Pond S samples (2018-09-20) marked red in Fig. S5.2a or the actual small sample size with N = 4.


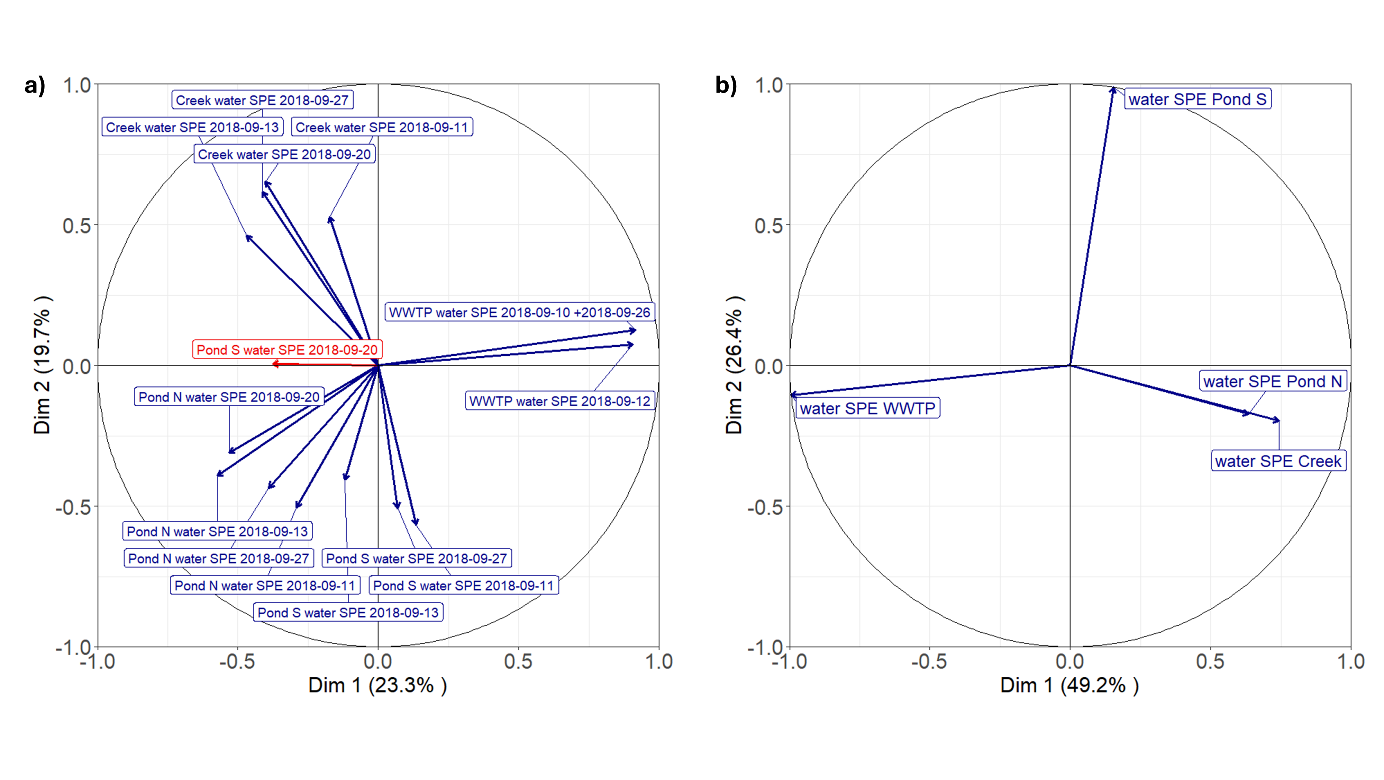
**Figure S2.2a-b:** PCA for water. a) time points separately; one outlier is marked in red color; b) combined using the mean value of the different time points for each site.

### S8.1.3 Sediment

The PCAs performed based on sediment data are shown in Fig. S4.3. In case of the PCA results of the comparison of all sites including Zones 1-5 (Fig. S4.3a), the first two dimensions Dim 1 and Dim 2 can explain 24.2% and 20.2%, respectively. The WWTP with ‑0.26 for Dim 1 and ‑0.89 for Dim 2 is quite different from the other sites, which show mostly positive values for Dim 2, except two samples just below zero (Creek: Dim 1: -0.48, Dim 2: -0.05; Zone 5: Dim 1: 0.68, Dim 2: -0.06). Zones 1-2 and Pond S cluster quite closely together in the quadrant negative Dim 1 / positive Dim 2, which was expected as these Zones are located in Pond S. Zones 3-5, and a bit off also Pond N, cluster together in the quadrant positive Dim 1 / positive Dim 2, disregarding the mentioned minor deviation of Zone 5. Interestingly, the vector representing Zone 3 is closer to the ones from Zone 4-5 and Pond N. This might me due to the fact that it is located in Pond S, but further away from its FHM site and Zones 1-2, adjacent to Pond N (ref. S1; Fig. S2). The proximity in the PCA implies that these sediments are more similar to each other.

Fig. S4.3b shows the PCA using the aggregated values for Pond S + Zones 1-3 and Pond N + Zones 4-5; while the values for Creek and WWTP stayed the same as in Fig. 4.3a. Here, the aggregated vectors for the Ponds are very similar as they almost overlap perfectly, whereas Creek and WWTP are very different to them and to each other. Yet, this might be less meaningful because of the small sample size of N = 4.


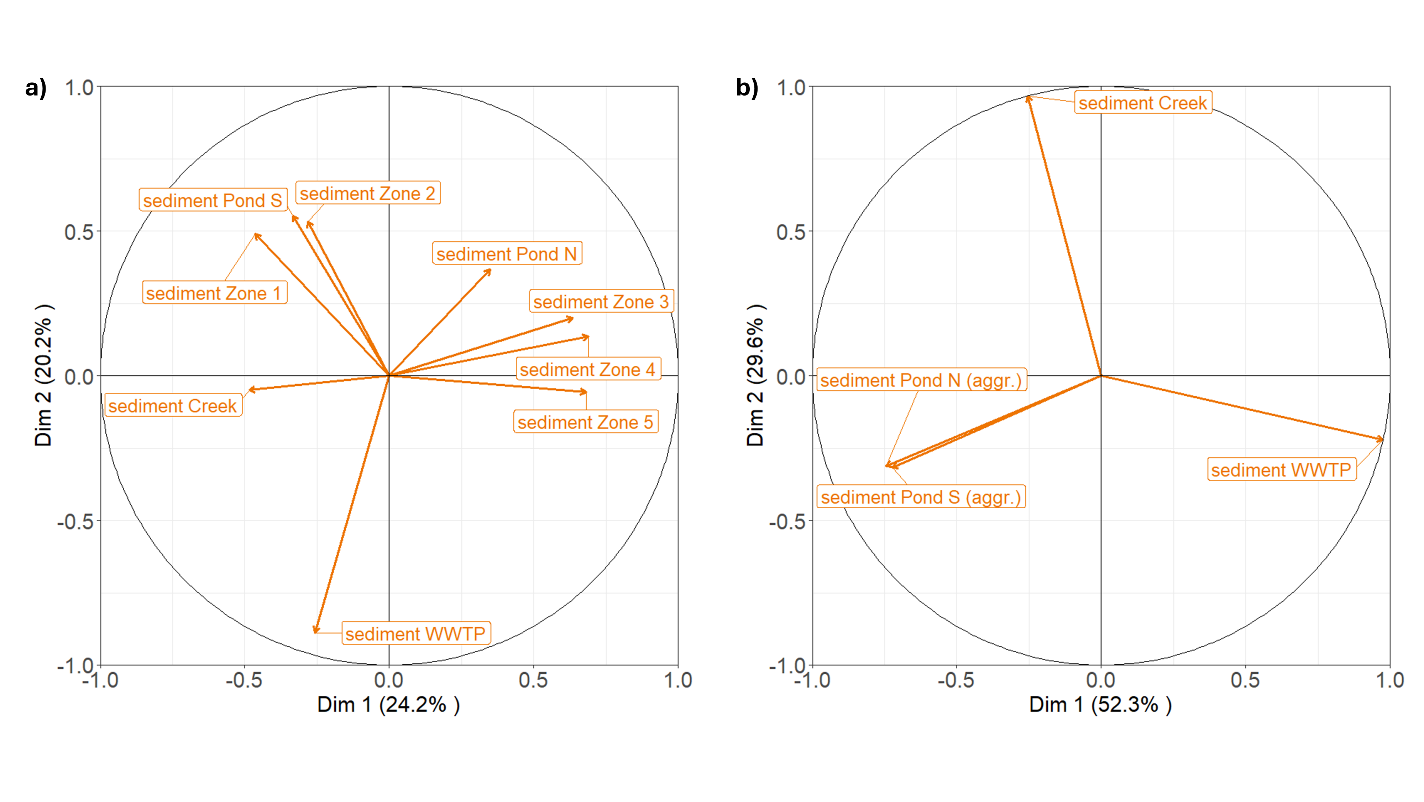


**Figure S2.3a-b:** PCA of sediment values. a) all sites separately: FHM sampling sites and additional “Zone” sites; b) with aggregated values for Ponds and Zones.

### S8.1.4 FHM, water, and sediment

The PCAs on combined datasets FHM/water/sediment are shown in Fig. 4.4a-d. The fact that the vectors water, sediment, and FHM are each clustering together in a different quadrant of the PCA plot in Fig. 4.4a (water: negative Dim 1/positive Dim 2; sediment negative Dim 1/negative Dim 2; FHM, both 2-d and 21-d: positive Dim 1/positive Dim 2) shows that the contamination seems to be matrix-related rather than site-related. In Fig. 4.4b, the vectors representing water and sediment are split between positive (sediment) and negative (water) values for Dim 1. Dim 2 is positive for all vectors, except for sediment WWTP, which implies that this sample is especially different. The PCA for FHM/water (Fig. 4.4c) and FHM/sediment (Fig. 4.4d) both split between FHM and the other matrix along Dim 1 and the time points 2‑d/21‑d of the FHM along Dim 2, underlining the differences between matrices.


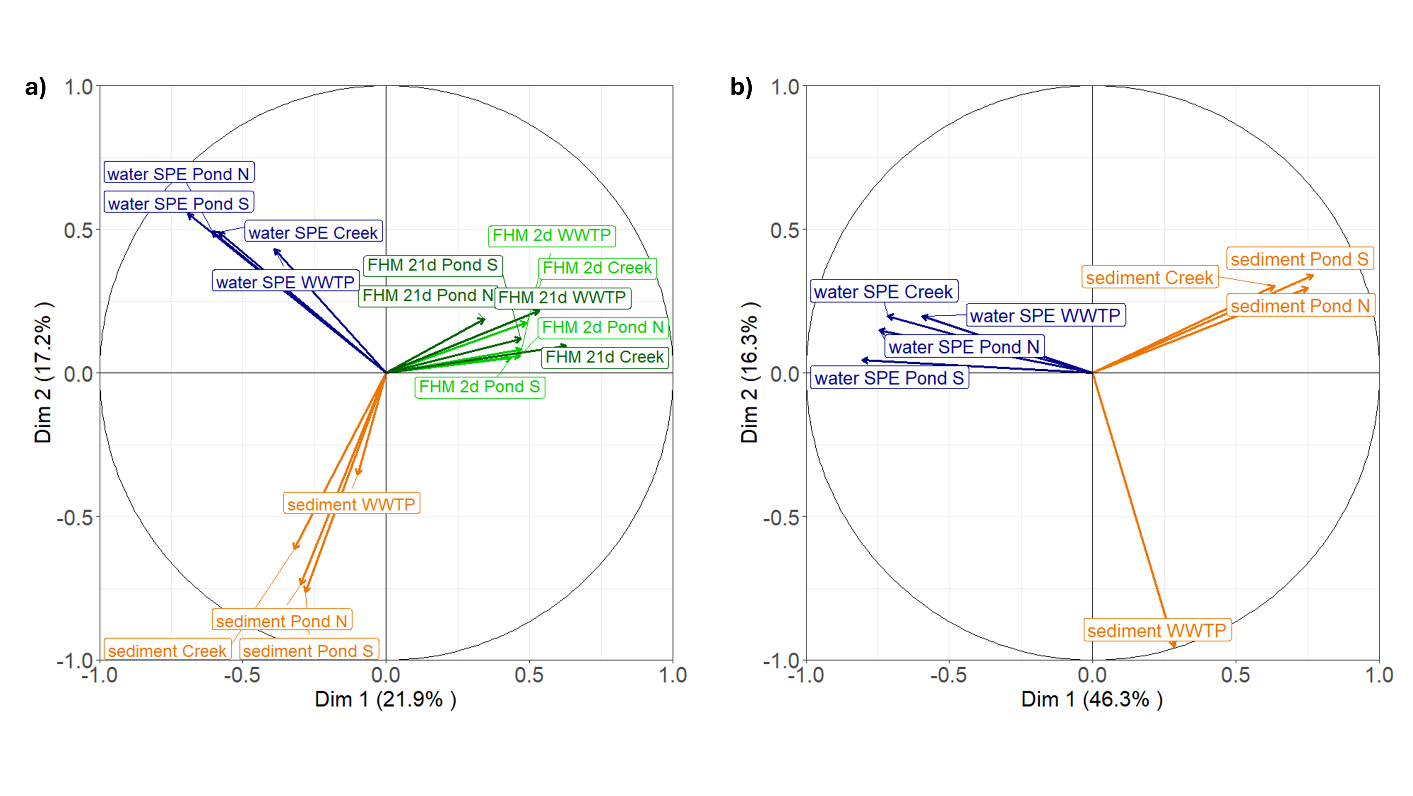

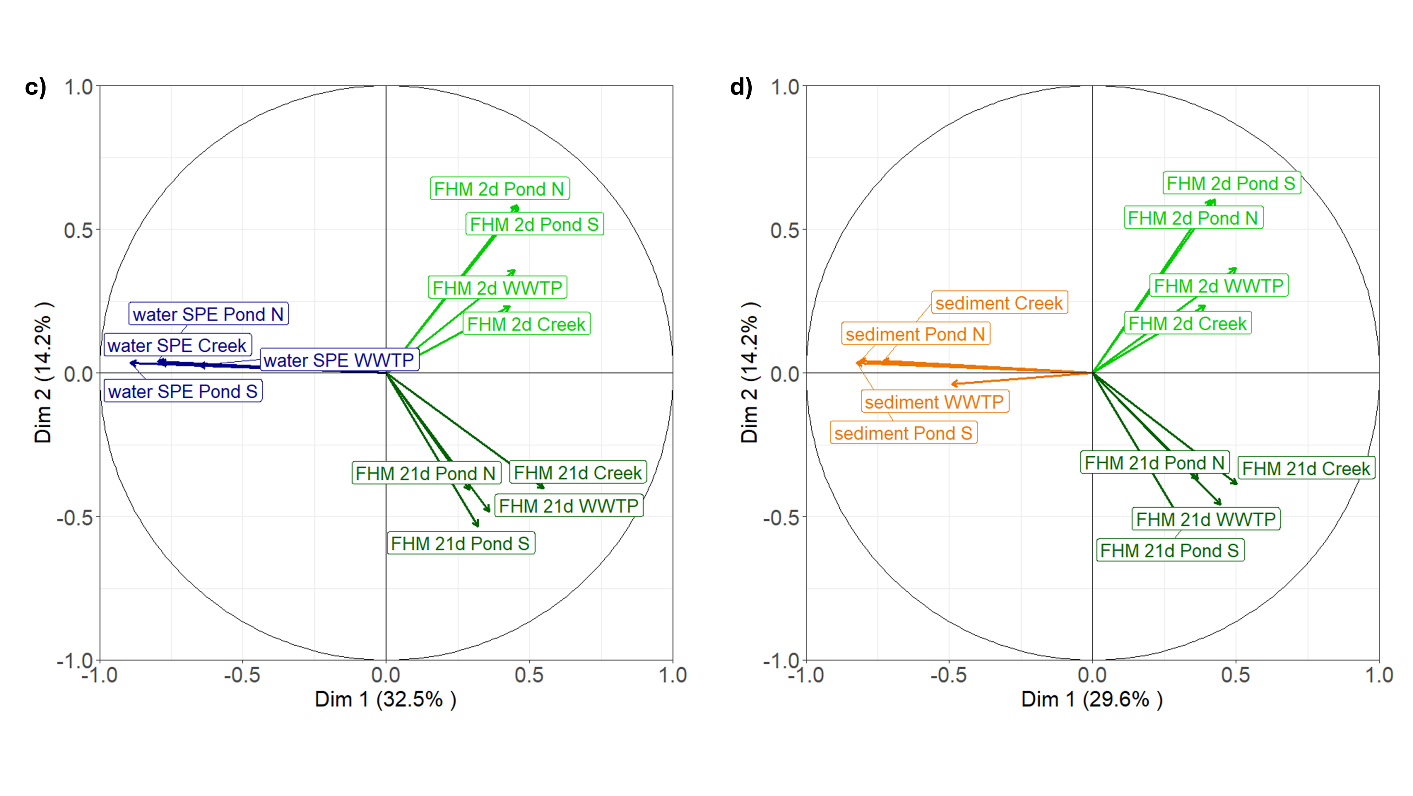


**Figure S2.4a-d:** PCAs from combined datasets a) for water, sediment, and FHM; b) for water and sediment; c) for FHM and water; d) for FHM and sediment. Colors: blue: water, orange: sediment, light green: 2-d FHM, dark green: 21-d FHM.

## S8.2 Pearson Correlation Analysis

A Pearson correlation analysis was performed on a combined dataset containing 2-d FHM, 21-d FHM, water, and sediment data, the resulting matrix is presented in Fig. 3.1 (log-transformed and standardized concentration data used). It shows stronger positive correlations between samples from one of the matrices and negative correlations between the different matrices. For water and sediment it is visible that the correlations between the Ponds are strong (water: R = 0.73; sediment: R = 0.61), the correlations between Creek and the Ponds are moderate (Creek to Pond S/N in sediment: R = 0.44/0.39; Creek to Pond N in water: R = 0.48, Creek to Pond S in water: R = 0.64), whereas the correlation between WWTP and the other sites are not existing for sediment (R = 0.06-0.07) and weak for water (R = 0.19-0.32). In case of FHM, the correlations between the different time points and sites are generally weaker than for sediment and water.


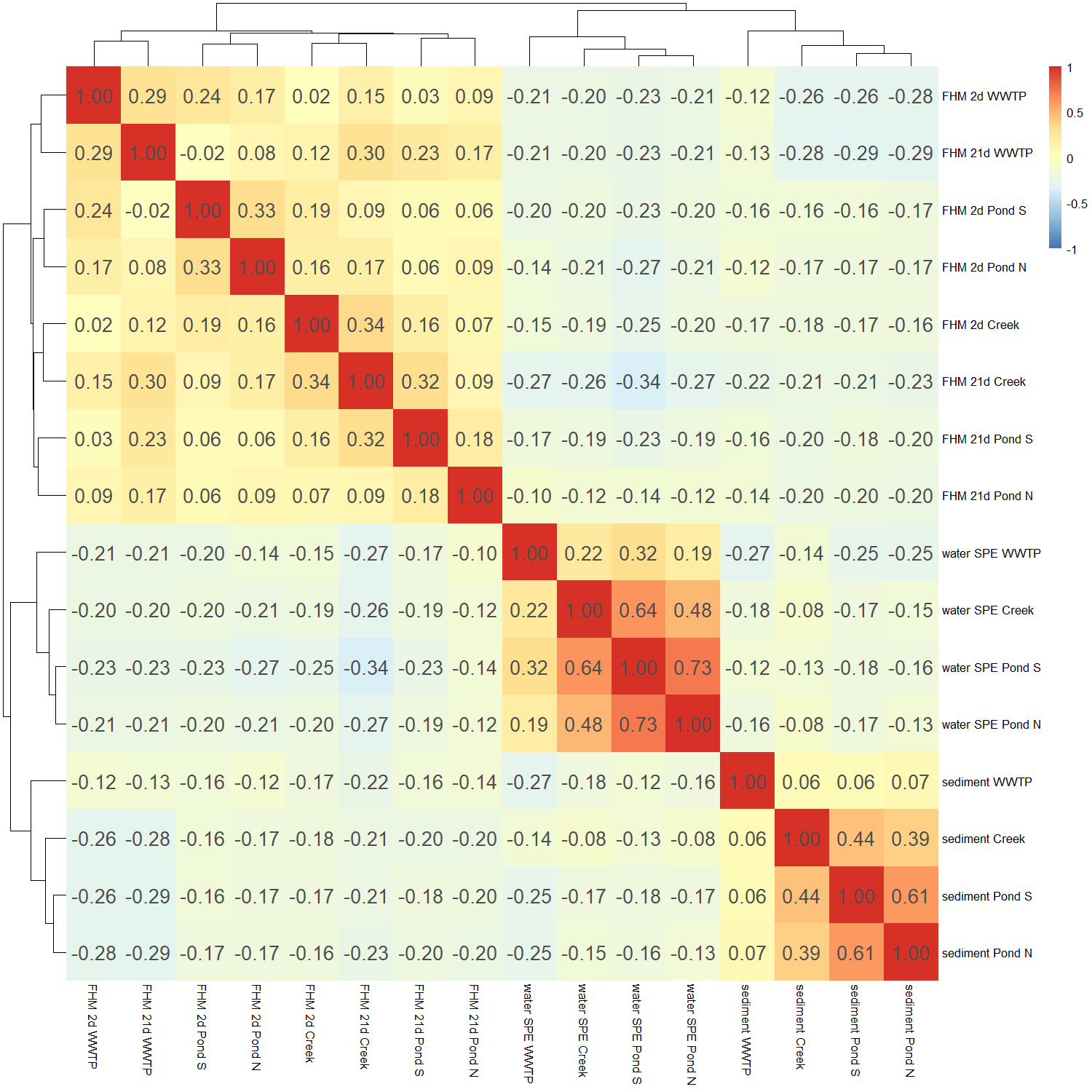


**Figure 3.1:** Pearson **C**orrelation matrix of the log-transformed standardized concentration data of 2-d FHM, 21-d FHM, water, and sediment.

## S8.3 Summary of statistical analysis

The different statistical approaches PCA and correlation analysis imply that the matrices FHM (2-d/21-d), water, and sediment all show different contamination patterns which hardly overlap. The matrices differ more from each other than the four sampling sites within each matrix. However, the similarities between the sites are in all matrices the same: most similar to each other are Pond S and Pond N, which are still similar to Creek which is still somewhat similar to WWTP.

# S9. Distribution and co-occurrence of compounds among the samples

Graphs generated with the UpSet package *ComplexUpset* in R to show common presence and distribution of compounds in water (Fig. S4.1), sediment (Fig. S4.2) and FHM (2-d: Fig. S4.3, 21-d: Fig. S4.4) at the four sampling sites, as well as all four kind of samples compared independently from sites (Fig. S4.5). Note: Figure S4.1 and S4.2 still include the substances which were excluded from the main evaluation of this study, which had been done because they were not measured in FHM.


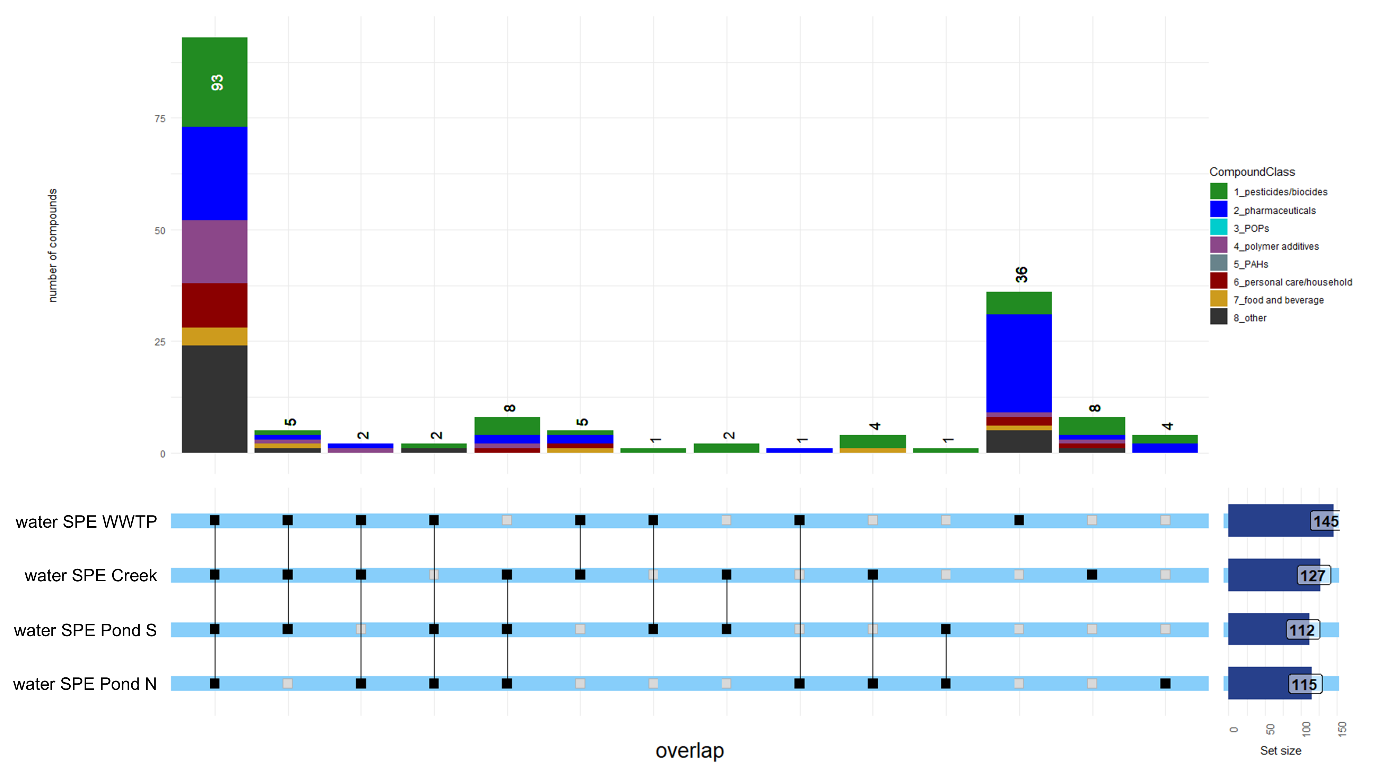


**Figure S4.1:** UpSet plot water.


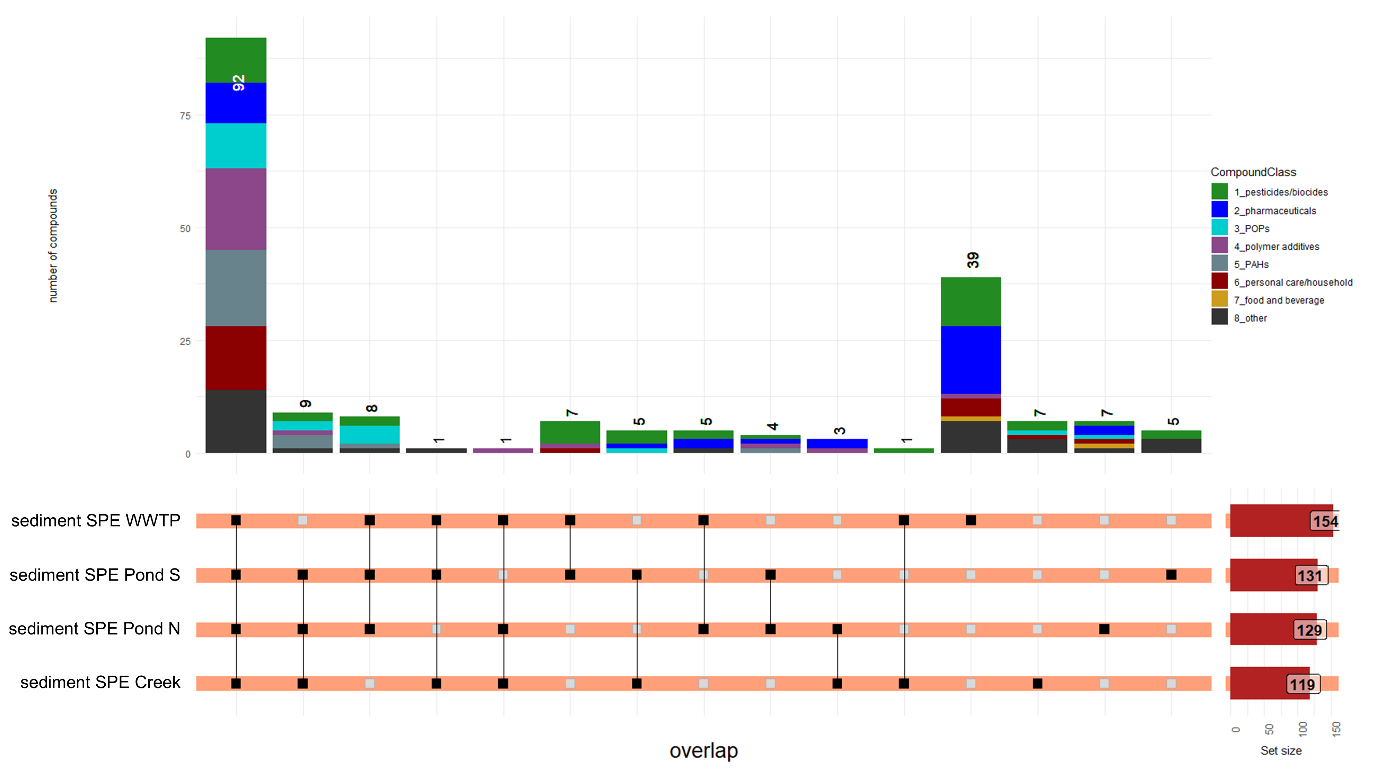


**Figure S2.2:** UpSet plot sediment.


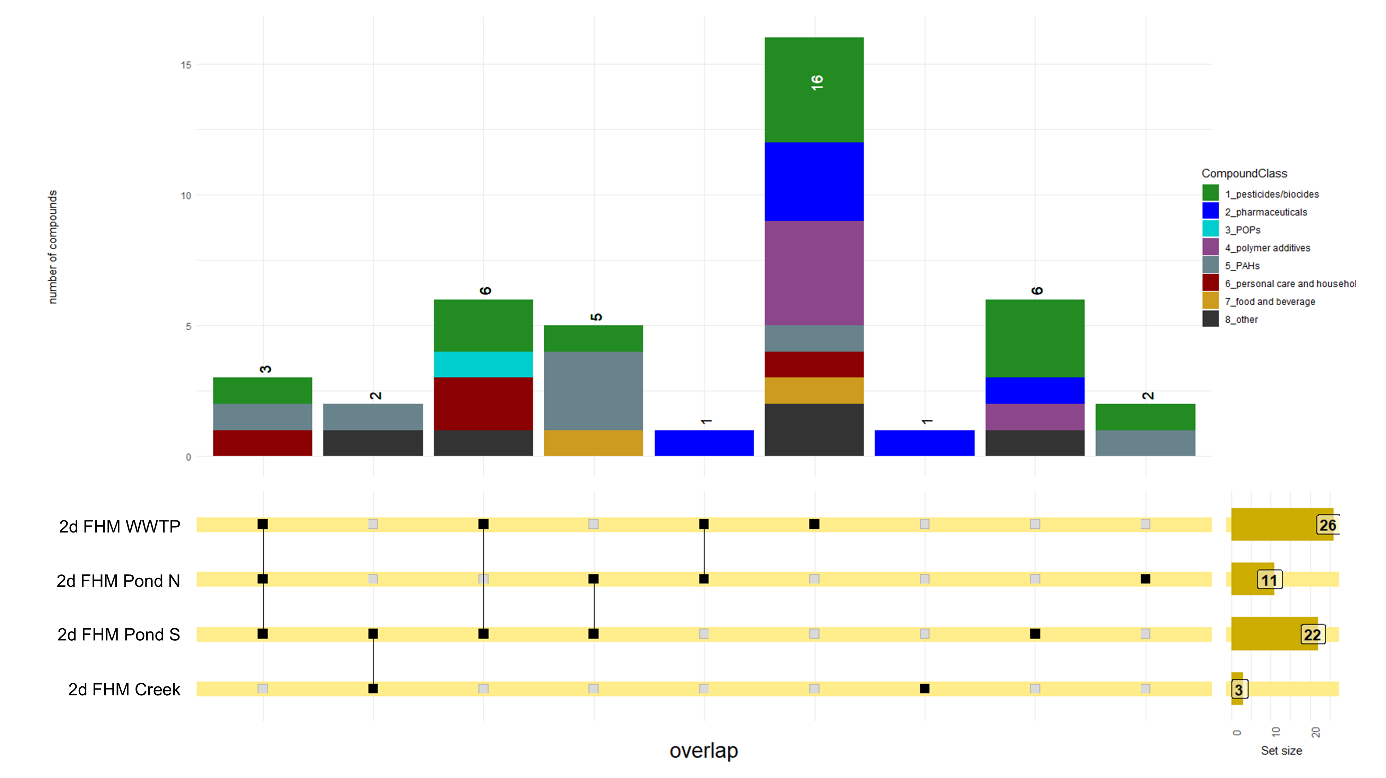


**Figure S4.3:** UpSet plot 2-d FHM (maximum values).


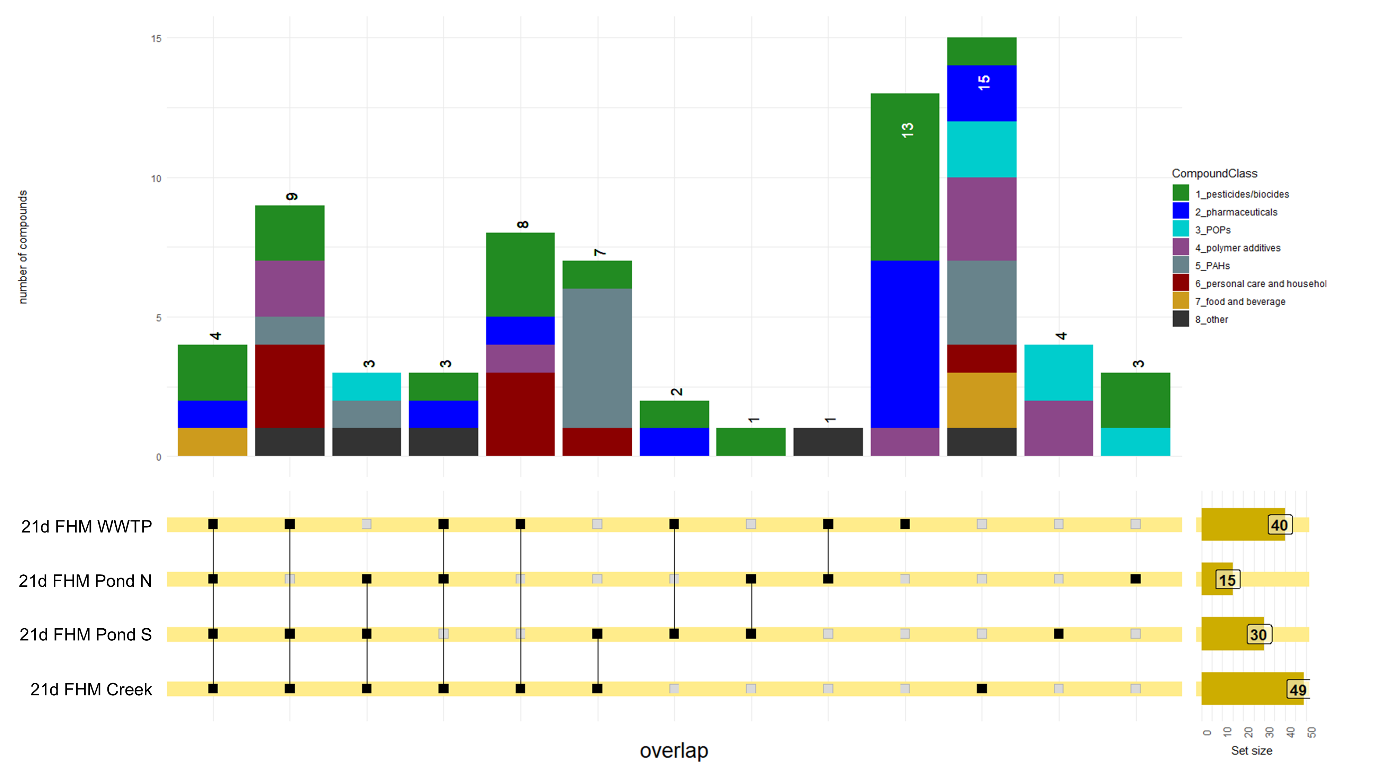


**Figure S4.4:** UpSet plot 21-d FHM (maximum values).


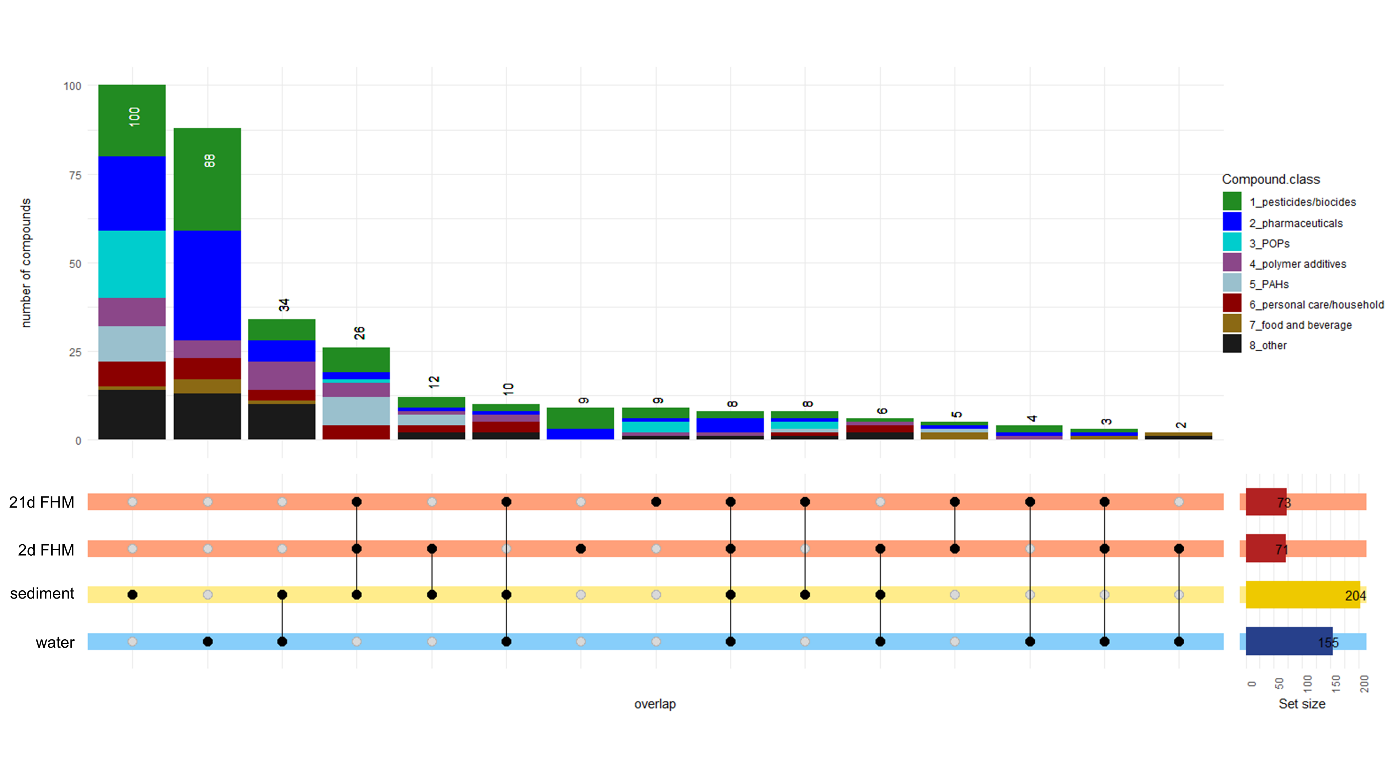


***Figure S4.5:*** *overlap between water, sediment, FHM 2-d and FHM 21-d.*

# S10. Co-occurrence of chemicals in water, sediment, and FHM

There was a tendency for more hydrophobic chemicals to be present in sediment and fish as the association with log *K*_ow_ in Fig. 2b demonstrates: comparatively hydrophilic chemicals with log *K_ow_* ≤ 2 were mainly found in the water, whereas more hydrophobic chemicals with log *K_ow_* ≥ 2 were detected in sediment and the 21-d fish samples. This finding is consistent with expectations of potential bioaccumulation of hydrophobic chemicals.^6-9^ The different compound classes were widely distributed over the log *K_ow_* scale and members of every class could be found in the 21-d FHM (Fig. S3).

Table S2 enables a closer look on the overlap of detects and non-detects in each water and sediment with FHM, independent of site, but separated for the time points of detection in case of FHM and water at 2 and 21 days. As mentioned, most substances were detected in water and/or sediment, but not in any FHM. This is depicted by in total 311 detects in water at 2-d and/or 21-d without corresponding detects in FHM. Analogous, 360 detects in sediment lacked corresponding detects in FHM. Most substances found in the water samples were detected at both time points. Thus, the contamination can be considered as stable over the time period of this study in most cases (cf. also SM-A, S6 for more detailed discussion of the constancy of concentrations in the water samples). As shown in Table 1, of the substances not detected at both timepoints in water, but either only at 2-d or only at 21-d, no more than 10 detects overlap with detects in FHM samples. Therefore, this rare inconstancy in water concentrations affects only a small number of compounds, yet it might impede further interpretation due to the resulting fluctuation in exposure in these few cases.

**Table S2**. Overlaps of detects and non-detects of FHM with the ones in either water or sediment across all sites, the former distinguished by the two timepoints 2-d and 21-d in case of FHM and water. The substances which were not measurable in FHM with the applied method, but might have been detected in water and/or sediment, are not included in this table. *Note that 97 substances were not measured in water, they are included in the non-detects.

| *number of overlaps* | | **FHM** | | | | **∑** | **% of detects in X found in FHM** |
| --- | --- | --- | --- | --- | --- | --- | --- |
|  |  | detected | | | not detected |  |  |
|  |  | only 2-d | only 21-d | 2-d & 21-d |  |  |  |
| **water** | only 2-d | 1 | 0 | 2 | 38 | 41 | **15** |
|  | only 21-d | 2 | 3 | 2 | 30 | 37 |  |
|  | 2-d & 21-d | 11 | 20 | 12 | 243 | 286 |  |
|  | not detected* | 47 | 57 | 31 |  | 135 |  |
| **% of detects in FHM found in water** | | **23** | **29** | **34** |  | **28** |  |
| **sediment** | detected | 36 | 41 | 36 | 360 | 473 | **24** |
|  | not detected | 25 | 39 | 11 |  | 75 |  |
| **% of detects in FHM found in sediment** | | **59** | **51** | **77** |  | **60** |  |

| 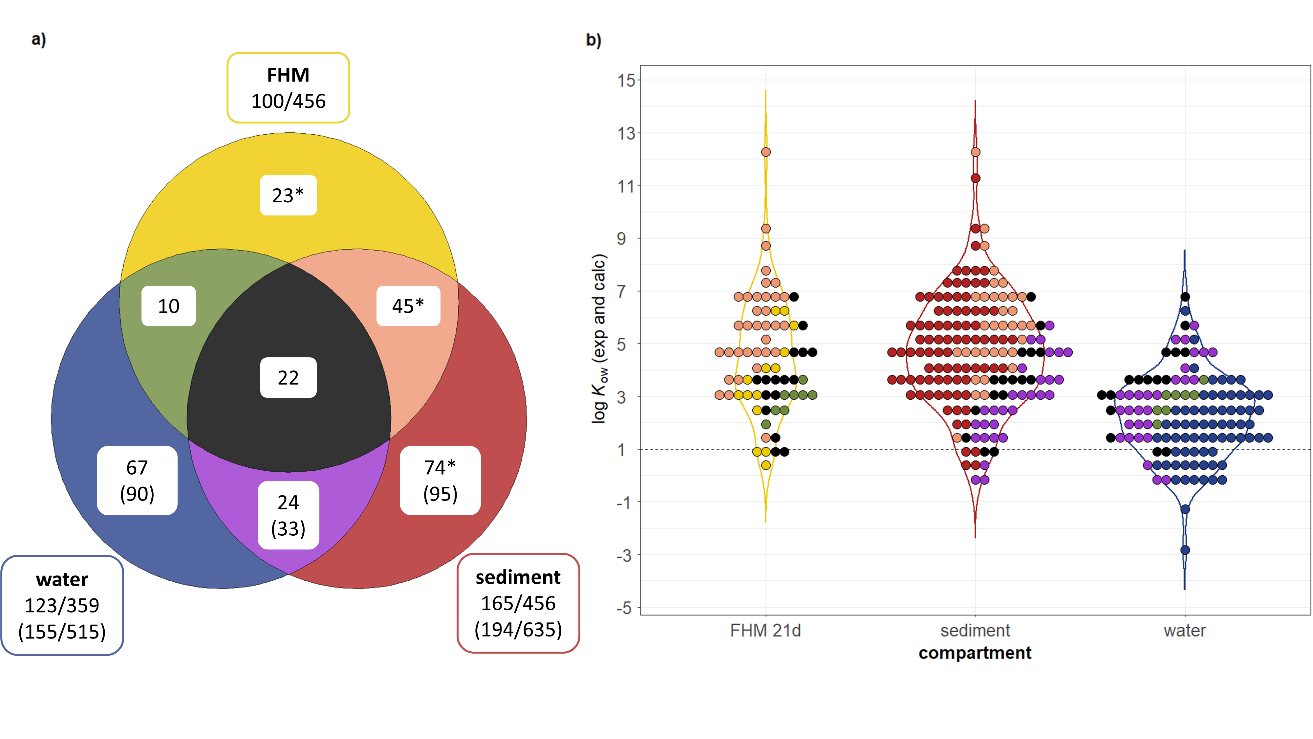 |
| --- |
| 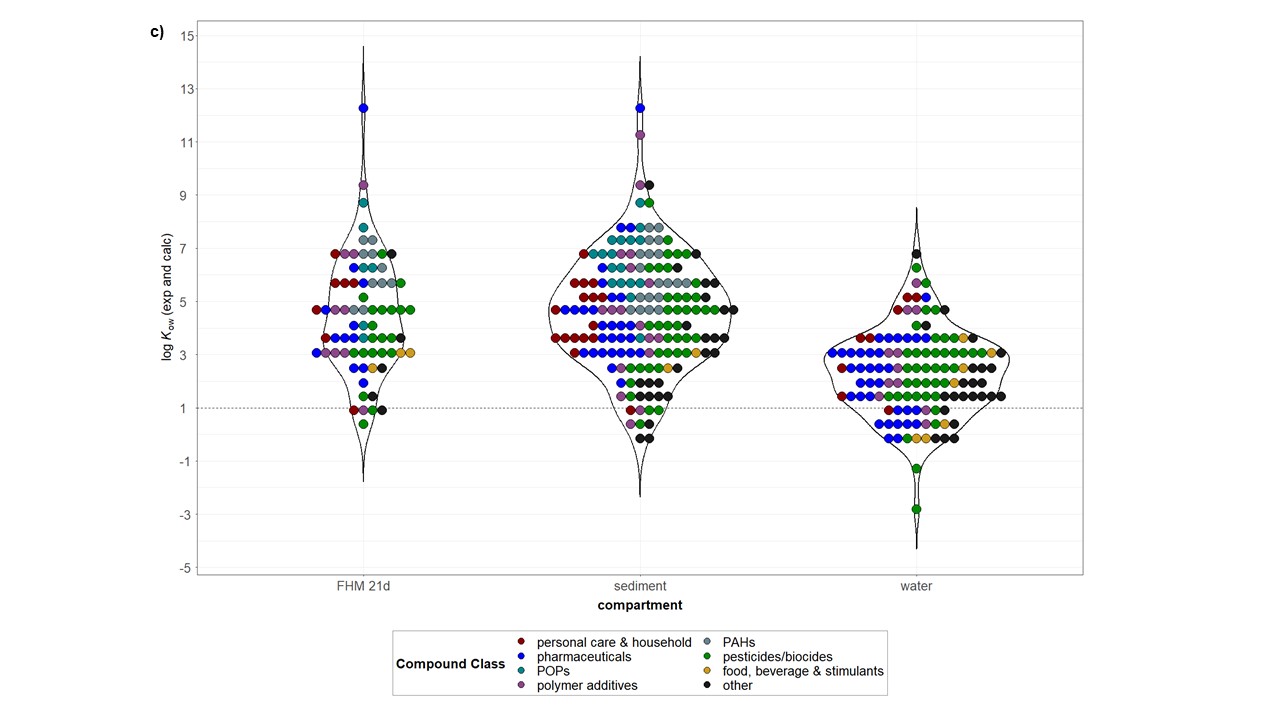 |
| **Figure S5.** a) Venn diagram of the co-occurrence of detected substances in water, sediment, and FHM; blue: chemicals only found in water (N = 67), dark red: only in sediment (N = 74), yellow: only in FHM (N = 23), violet: in water and sediment, but not in FHM (N = 24). light orange: both in sediment and FHM samples (N = 44), green: water and FHM (N = 10), black: in all three sample types (N = 22). b) occurrence relative to the log *K*_ow_ of the neutral species. Bin width setting concerning the grouping of the dots for the log *K*_ow_ is 1/30 of the range of the data. The color code in a) is also used for the violin plot b). In c), the color represents the compound class of each substance. Note that every dot represents one substance and that dots can occur more than once if the substance is present in more than one compartment. Data from SM-B,Table S9. |

# S11. Time- and site-dependent comparison of 2-d FHM, 21‑d FHM, water and sediment concentrations

The following section refers to section 4.3 of the main text. In Figure S4a-c, the concentrations of the substances detected in water, sediment, and FHM were compared dependent on time and site as an example for the applied A-/E-/V-patterns and the agreement of co-occurrence in water and/or sediment. The dataset was divided into three subsets in respect to the different occurrences of substances in the FHM. The substances detected in *FHM at 2-d* *but not at 21‑d* (see S10.1) are displayed in Fig. S4a*, FHM at 21-d but not at 2-d* (S10.2) are displayed in Fig. S4b, and *FHM detected at both 2-d and 21‑d* (S10.3) are displayed in Fig. S4c. Additionally, substances *detected in water and sediment, not in FHM* (S10.4), ones detected in either *only water* (S10.5) or *only sediment* (S10.6) are discussed in the following sections. To complete discussion of the dataset, the abundance of target substances in the FHM controls are discussed in detail in section S10.7.


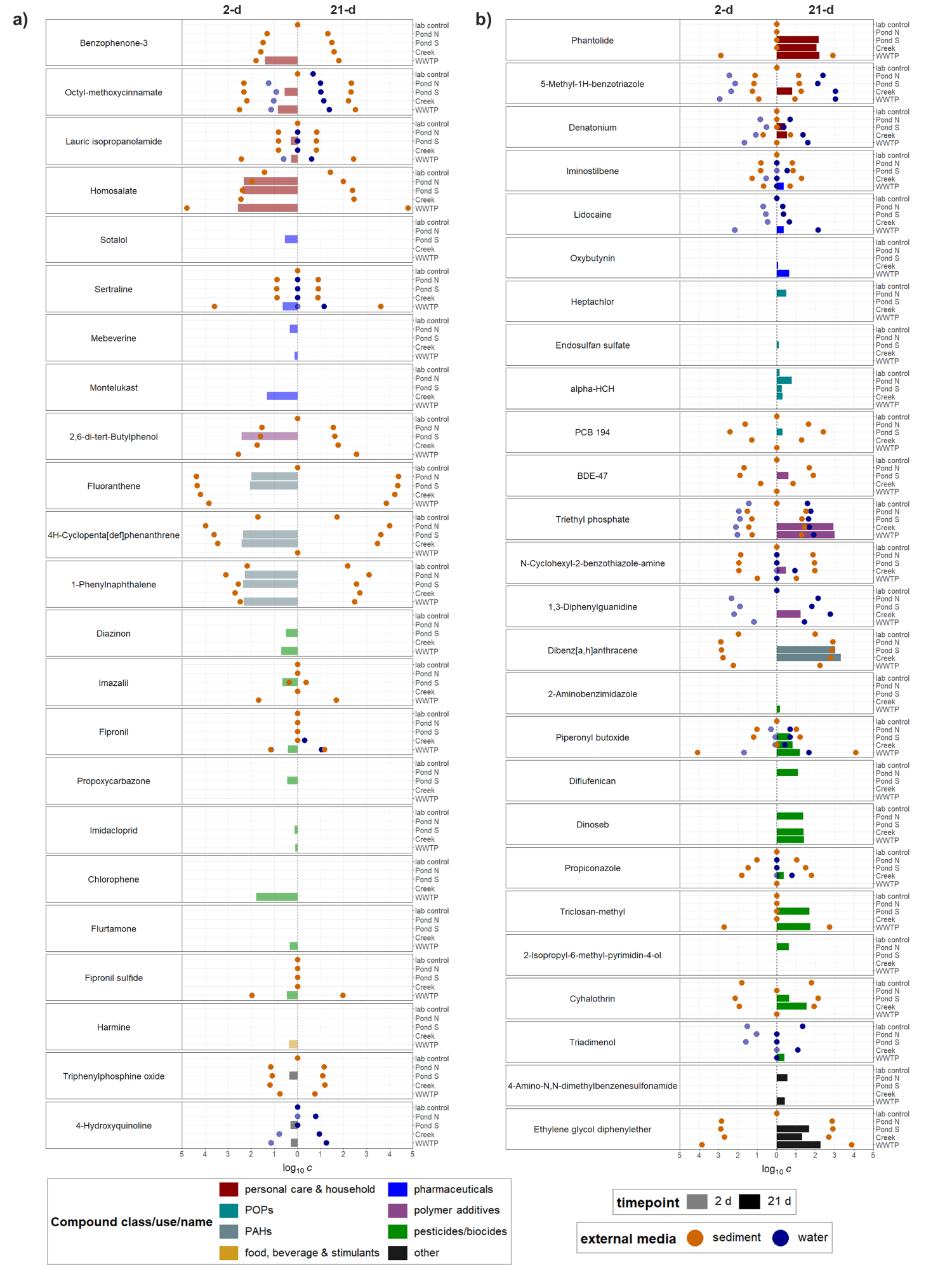


**
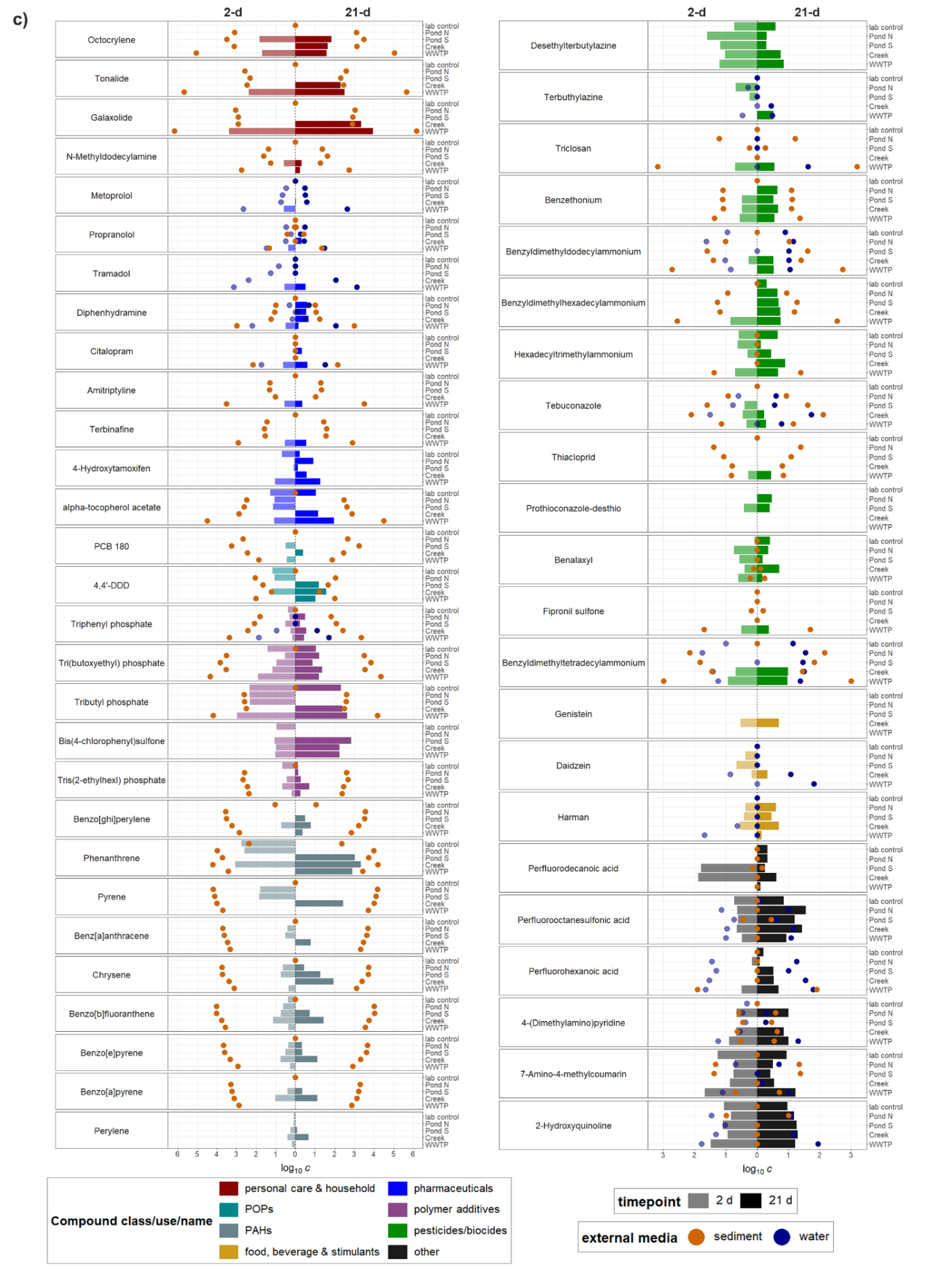
**

**Figure S6. a) site- and time-dependent concentrations of substances only found in 2-d FHM; b) substances only found in 21‑d FHM; c) substances found in both 2-d and 21-d FHM.** The left side of each plot represents the 2-d concentrations, the right side the 21-d concentrations in the different matrices. All concentrations (FHM, water, sediment) were transformed into log_10_ values, except for original values < 1, where log(1+x) was used. Only substances with at least one value above the applied control threshold for FHM are shown, but all values are included independent of this threshold. The bars represent the FHM concentrations, the dots water (blue, both 2-d and 21-d) and sediment (orange, only 21-d) concentrations, the latter also included in the 2-d side of the plot. Note that the original concentration units were ng/g_ww_ for FHM, µg/L for water and mg/kg_OC_ for sediment.

## S11.1 Substances only detected in 2-d FHM

Overall, 23 substances were only detected in 2-d FHM but not in 21-d FHM. A subset of these substances could not be detected in FHM after the 21‑day period even with a measurable concentration in the water or sediment, which might be explained by an induced metabolism. These compounds could be interpreted to be eliminated by the FHM after first uptake, representing by definition the established E-pattern.

Furthermore, nine of these compounds were neither detected in water nor sediment (three *pharmaceuticals*: sotalol, mebeverine, montelukast; five *pesticides/biocides*: diazinon, propoxycarbazone, imidacloprid, chlorophene, flurtamone; one *food, beverage & stimulant*: harmine), even though the applied methods should be feasible to detect them.

In three cases the abundance in the 2-d FHM matches the abundance patterns in the sediment (one *pharmaceutical*: sertraline; two *pesticides/biocides*: fipronil and its transformation product fipronil sulfide).

In additional 10 cases, the substances detected in the 2-d FHM were detected in the sediment from the same site, but not *vice versa* (four *personal care & household*: benzophenone-3, homosalate, octyl-methoxycinnamate, and lauric isopropanolamide; one *polymer additive*: 2,6-di-tert-butylphenol; three *PAHs*: fluoranthene, 4H‑cyclopenta[def]phenanthrene, 1‑phenylnaphthalene; one *pesticide/biocide*: imazalil; one *other*: triphenylphosphine oxide). Two of these compounds were also detected in water at the same sites (octyl-methoxycinnamate) or at the site with the highest concentration in sediment (lauric isopropanolamid), at both time points in similar concentration ranges. Despite the apparently constant concentration over time, it was only found in 2-d FHM.

Sertraline was detected in water at the same site as the FHM (WWTP), but only in the 21‑d sample. As this sample is mistakenly mixed-up of the extract from the beginning and end of the campaign (10th + 26th September 2018) it might be the rare case that this compound was only from the first time point.

## S11.2 Substances only detected in 21-d FHM

26 substances were only detected in 21-d FHM, meaning that these were accumulated over time or were at least persistent to a certain degree, therefore all representing the A‑pattern.

Nine of these compounds were neither detected in water at 2-d, water at 21-d nor sediment (one *pharmaceutical*: oxybutynin; three *POPs*: heptachlor, endosulfan sulfate, alpha-HCH; four *pesticides/biocides*: 2-aminobenzimidazole, diflufenican, dinoseb, 2-isopropyl-6-methyl-pyrimidin-4-ol; one *other*: 4-amino-N,N-dimethylbenzenesulfonamide). Note that dinoseb was measured in water, but only at the other two timepoints which were left out for this comparison.

In one case the (qualitative) abundance in the 21-d FHM aligns with that in sediment (cyhalothrin; not detected in water). In two other cases the abundance in the 21-d FHM aligns with that in water (1,3-diphenylguanidine, lidocaine; not detected in sediment), when an MDL limitation is considered.

Similar to substances only detected in 2-d FHM, there were 9 substances detected in the 21‑d FHM and in the sediment from the same site, but not *vice versa*. In 2 cases, only a detect on pond N is missing, which might be due to the mentioned likely lack of nutrition at that site (dibenz(a,h)anthracene and ethylene glycol diphenylether), implying a link to food uptake and lipid content in the tissue. In five cases the abundance in 21‑d FHM matches the site with highest concentration in sediment, indicating that the MDL might not be sufficient for detects at the other sites, presumed that the sediment is the only source of contamination. However, three of these five were detected in water (5-methyl-1H-benzotriazole, N-cyclohexyl-2-benzothiazole, propiconazole), whereas two of them were not (PCB 194, BDE-47). The water concentrations of 5-methyl-1H-benzotriazole do not coincide with a presence in FHM, whereas the presence of N-cyclohexyl-2-benzothiazole and propiconazole align with the presence in FHM at Creek. Iminostilbene and triethyl phosphate were detected only at part of the sites without any recognizable relation to neither sediment nor water concentrations.

Four compounds (phantolide, denatonium, piperonyl butoxide, and triclosan-methyl) were detected in both FHM and sediment, but also at sites where they were not detected in sediment. However, denatonium and piperonyl butoxide were detected at all sites and both timepoints in water, leaving only the absence of denatonium at WWTP unexplained, if the missing detects at pond N are reasoned with as done above.

Triadimenol was detected in 21-d FHM and water, but not in sediment. It only occurs at the WWTP in FHM, whereas the estimated concentration in water exceeded the control only in the 2-d sample at Pond S.

## S11.3 Substances detected in 2-d and 21-d FHM

40 substances were detected in both 2-d and 21-d FHM at any site. As mentioned for these substances the picture is more complex, the possible patterns are A and V.

6 of these compounds were neither detected in water at 2-d, water at 21-d nor sediment (1 *pharmaceutical*: 4-hydroxytamoxifen; 1 *polymer additive*: bis(4-chlorophenyl) sulfone; 1 *PAH*: perylene; 2 *pesticides/biocides*: desethylterbutylazine, prothioconazole-desthio; 1 *food, beverage & stimulant*: genistein. Of these compounds, prothioconazole-desthio and genistein resembled the A-pattern, all others are discussed below.

The abundance of 3 substances aligns between sediment and FHM in a way which represents the A-pattern: triphenyl phosphate, benzethonium, and benzyldimethylhexadecylammonium. Triphenyl phosphate was also detected in water at both timepoints 2-d and 21-d, but only at two of the four sites.

Again, there were substances which were detected in sediment and in FHM, but at some sites not in the latter, of which 15 substances matching the A-pattern: 4 *personal care & household*: octocrylene, tonalide, galaxolide, N-methyldodecylamine; 3 *pharmaceuticals*: diphenhydramine, amitriptyline, terbinafine, 2 *PAHs*: benzo(ghi)perylene, benzo(a)pyrene; 5 *pesticides/biocides*: triclosan, benzyldimethyldodecylammonium, thiacloprid, fipronil sulfone, benzyldimethyltetradecylammonium; 1 *other*: 4-(dimethylamino)pyridine. In several cases, detects in FHM from pond N at 21-d were missing even with concentrations in sediment detected, which were comparable to the one found at the other sites. 5 of the named substances were also detected in water (diphenhydramine, triclosan, benzyldimethyldodecylammonium, benzyldimethyltetradecylammonium, 4-(dimethylamino)­pyridine). In case of triclosan, contrary to the concentrations found in sediment, the occurrence in water matches the one in FHM regarding sites, but not regarding timepoints as it was not detected at 2-d. Diphenhydramine was detected at all sites in FHM, but at 2-d only at WWTP, matching the highest concentration in water at both timepoints. 4‑(dimethylamino)pyridine was detected at all sites (and timepoints) in water and sediment, but showed a gap in the FHM detects at pond S, where the lowest concentrations were detected in sediment and water, implying an MDL limitation. The patterns of benzyldimethyldodecylammonium and benzyldimethyltetradecylammonium in water were quite similar across matrices: present at all sites and timepoints except 2-d pond S, with abundance in FHM at Creek and WWTP.

Tramadol (*pharmaceutical*) was detected at all sites at 2-d in water, but at 21-d only at Creek and WWTP, the latter being the site with the highest concentration at both time points, which is also the site where it was detected in FHM at both timepoints (A-pattern) – implying an MDL limitation for the other sites. Metoprolol and Propranolol (*pharmaceuticals*), which were detected at all sites and timepoints in water, showed a V-pattern in FHM matching the occurrence in water samples at the timepoint/site-combinations it was detected (2-d WWTP, 21-d Creek), with Propranolol being detected in sediment at pond S and WWTP therefore mismatching the detect at Creek. The occurrence of harman (*food, beverage & stimulants*) resembles an A-pattern in the FHM at all sites, but mismatches the detects in water (only at 2‑d at Creek and WWTP).

Citalopram (*pharmaceutical*) showed an A-pattern in FHM (2-d and 21-d at WWTP, 21-d at pond S) partially matching the occurrence in water, and sediment (only found at WWTP; water at both 2-d and 21-d).

The 6 *PAHs* pyrene, benz(a)anthracene, chrysene, benzo(b)fluoranthene, benzo(e)pyrene, and perylene showed a V-pattern in the FHM samples, all with a maximum concentration at 21-d at Creek. All of these substances except perylene were detected in sediment at all sites. Pyrene and benz(a)anthracene were detected at 2-d in FHM from both ponds, while at 21-d only at 21-d Creek. Regarding chrysene and benzo(e)pyrene only because of the absence of detection in 21-d WWTP it is a V-pattern, otherwise they would be classified as A-pattern, same applies to benzo(b)fluoranthene, but additionally a detect in 21-d pond N is missing. Perylene matches the A-pattern when the FHM control threshold is applied, with detects at both timepoints at pond S and Creek.

Besides the already mentioned, 4 additional substances occurred in a V-pattern in the FHM: PCB 180, terbuthylazine, tebuconazole, and daidzein. PCB 180 (sediment) and tebuconazole (sediment and water) were detected at all sites, matching the detects in FHM regarding sites except for pond N, but the time points of detects are varying in FHM. The detection pattern of terbuthylazine in water partly matches (pond N: only 2-d, WWTP: 21-d) and mismatches (pond S: 2-d in FHM, WWTP: 2-d in water) the pattern in FHM. Similarly, daidzein was detected in water and FHM, with matching (Creek: 2-d and 21-d) and mismatching (2-d at both ponds in FHM, 21-d at WWTP in water) occurrences. For 15 substances, the concentration in at least one the FHM controls was close to or within the same order of magnitude as in the samples from the sites, which complicates the interpretation of the pattern (4‑hydroxytamoxifen, alpha-tocopherol acetate, 4,4’-DDD, tri(butoxyethyl) phosphate, tributyl phosphate, bis(4‑chlorophenyl) sulfone, tris(2-ethylhexyl) phosphate, phenanthrene, desethylterbuthyl­azine, hexadecyltrimethylammonium, benalaxyl, perfluorooctanesulfonic acid, 7-amino-4-methylcoumarin, 2-hydroxyquinone). Of these substances, bis(4‑chlorophenyl) sulfone and perfluorooctanesulfonic acid seem to be the only ones showing a somewhat clear tendency to the A-pattern as the concentrations in the 21-d are significantly higher as in the 2-d, which were close to the one in the FHM controls.

One special case is perfluorodecanoic acid, with detects in FHM controls and samples, but with concentrations in one order of magnitude higher than in controls at pond S and Creek at 2-d, actually resembling an E-pattern. It was also detected in sediment in low concentrations at sites not matching the occurrence in FHM.

## S11.4 Substances detected in water and sediment, not in FHM

33 substances were detected in water and sediment, but not in FHM (above the control threshold), of which 24 would have been detectable by the applied extraction method. Of these 24 compounds, 9 were detected but did not exceed the threshold. The remaining 15 were 4 *polymer* *additives* (triethyl citrate, tris(2-chloroethyl) phosphate, tris(1-chloro-2-propyl) phosphate, 2(4‑morpholinyl) benzothiazole), 4*other* (4-aminobenzamide, 2-isopropylthioxanthone, tetraglyme, acridone, 4‑hydroxy-1-(2-hydroxyethyl)-2,2,6,6-tetramethylpiperidine), 3 *pesticides/biocides* (DEET, triclocarban, icaridin), 2 *personal care & household* (4-methylbenzylidene camphor, lauryl diethanolamide), 1 *pharmaceutical* (crotamiton).

## S11.5 Substances only detected in water

With 90 substances detected only in water and not in FHM (above the applied threshold), 67 of which the extraction method was feasible to detect them, a quite large fraction of the external contamination could not be tracked down in the target organism of this study. Disregarding the 23 compounds which were not measurable in the FHM and therefore its relation to any effect on the FHM remains unknown and 5 more which were discussed in other sections because they were found also in FHM controls, the remaining 62 compounds consisted of 24 *pesticides/biocides* (2,4‑dichlorophenoxyacetic acid, thiabendazole, metribuzin, hexazinone, dichlorprop, spiroxamine, bendiocarb, desethylatrazine, carbendazim, metolachlor, mecoprop, MCPA, chlorothalonil-4-hydroxy, 3,5,6-trichloro-2-pyridinol, propamocarb, 2‑hydroxyatrazine, acetamiprid, mepiquat, terbuthylazine-2-hydroxy, imidacloprid-guanidine, imazapyr, 2‑hydroxydesethylterbuthylazine, methiocarb-sulfoxide phenol, simazine-2-hydroxy), 22 *pharmaceuticals* (phenazone, hydrochlorothiazide, sulfamethoxazole, sulfapyridine, carbamazepine, primidone, pindolol, mycophenolic acid, 4-formyl-antipyrine, 2-hydroxycarbamazepine, 10,11-dihydro-10-hydroxycarbamazepine, amantadine, acetyl-sulfamethoxazole, bisoprolol, bupropion, memantine, losartan, valsartan, clonidine, lamotrigine, labetalol, phenylethylmalonamide), 8 *other* (4-nitrophenol, N‑ethyl-o-toluenesulfonamide, 2,4-dinitrophenol, bisphenol S, perfluorooctanesulfonamide, 6:2 fluorotelomer sulfonic acid, 1-butyl-3-methyl-imidazolium, dimethyl-5-sulfoisophthalate), 4 *food, beverage & stimulants* (caffeine, acesulfame, cotinine, 5-carboline), 2 *personal care & household* (1H‑benzotriazole, dicyclohexyl sulfosuccinate), and 2 *polymer* *additives* (N‑butylbenzenesulfonamide, 2-benzothiazolesulfonic acid).

For a subset of these compounds the non-detects in the FHM might be explainable due to insufficient MDL. Another reason might be a in comparison to the sampling period short metabolization time.

## S11.6 Substances only detected in sediment

A total of 95 substances were only detected in sediment, but not in FHM (above the control threshold), of which 74 would have been detectable by the applied extraction method. 20 were detected in FHM, but were excluded from the analysis because of the control threshold. The 54 detectable compounds consist of 16 pharmaceuticals (bezafibrate, clotrimazole, paroxetine, amiodarone, duloxetine, loperamide, miconazole, oxazepam, fenofibrate, celecoxib, efavirenz, fluvoxamine, clozapine, tamoxifen, nitrendipine, gabapentin-lactam), 9 POPs (hexachlorobenzene, PCB 52, PCB 101, PCB 28/31, PCB 44, PCB 118, PCB 138, 4,4'-DDMU, alpha-endosulfan), 6 pesticides/biocides (chlorpyrifos, azoxystrobin, fenpropimorph, 2-octyl-4-isothiazolin-3-one, hexadecylpyridinium, clothianidin, fenpropidin, imidacloprid-urea, dichlorophen, etofenprox, pyriproxyfen, metolachlor CGA 368208), 7 other (benzyl-2-naphthylether, 2,2-dimethoxy-2-phenylacetophenone, Michler's ketone, diphenylmethane, o-, m-, and p-terphenyl), 5 PAHs (benzo[k]fluoranthene, anthracene, dibenzo[a,e]pyrene, indeno[1,2,3cd]fluoranthene), 3 personal care & household (N,N-dimethyldodecylamine-N-oxide, celestolide, climbazole), 2 polymer additives (N‑phenyl-1-naphthylamine, BDE-99), 1 food, beverage & stimulants (norharmane).

In case of substances only detected in sediment, besides the possibility of explainable due to insufficient MDL, one additional reason might be strong sorption to the organic carbon in the sediment of some compounds with slow desorption kinetics (e.g. PCBs^10^). Because of the distance between the fish cages and the sediment, that kind of substances would not reach the FHM.

## S11.7 FHM controls

5 compounds were found in the FHM controls, of which 67 were detected also in the FHM in the field. Eight compounds (1 *pharmaceutical*: pentoxifylline; 6 *biocides/pesticides*: trifloxystrobin, pethoxamid, simetryn, quinoxyfen, bupirimate, fipronil desulfinyl, 1 *polymer additives*: TDCPP) were solely detected in the controls. 32 of these compounds could be related to their occurrence in food of the FHM (brine shrimp: 4; trout chow: 17; both: 11 compounds, see SM-B, S9), while others might be false-positives not measurable with the applied method. The likely contamination through the food applies to the controls over the whole period of this study to some extent (feeding of trout chow was discontinued at the start of this study, while feeding with brine shrimp continued), and for the FHM in the field up to deployment.

Besides the 8 compounds which were not detected in any FHM from the different sampling sites, a relatively high number of 42 compounds were excluded from any analysis in this study due to the applied threshold. Of these 42 compounds, 22 detects might be explainable due to the occurrence in the food (3 in brine shrimp, 10 in trout chow, 9 in both), leaving 20 others of unknown origin. Despite some of them also occurred in water (5), sediment (20) or both (9) at the study sites, it was not possible to state anything about the fate of these subset in the FHM, hence for simplification they were counted as “only in water”, “only in sediment”, or “in water and sediment, but not in FHM”, respectively.

# Besides the obvious need for meaningful control groups in the context of field studies like this, it becomes increasingly challenging, especially with increasing sensitivity of measurements and a growing number of target compounds, to obtain any fish (and food) which are “not contaminated by any of the targeted micropollutants. It is likely impossible to obtain contamination-free fish, as they have to be kept in larger volumes of water in tanks with aeration and circulation, and must be fed, both posing potential sources of contamination.

# S12. Comparison of 2-d FHM, 21-d FHM, water and sediment concentrations

Based on the data shown in 4.3.3 and Fig. 3, six compounds seem to be better represented by water than sediment concentration (✔ and (✔) in water, but (🗙), 🗙 in sediment): denatonium (*personal care & household*), piperonyl butoxide (*pesticides/biocides*), perfluorooctanesulfonic acid, perfluorohexanoic acid, 2-hydroxyquinoline (*other*), and propanolol (pharmaceuticals; V-pattern). Five compounds seem to be better represented by sediment than water concentration (✔ and (✔) in sediment, but (🗙), 🗙 in water): iminostilbene, sertraline (*pharmaceuticals*), triclosan, tebuconazole (*pesticides/biocides*), and lauric isopropanolamide (*personal care & household*). In addition to the 23 substances only detected in FHM (cf. S11.1-S11.3), 13 compounds seem to be present in FHM before reaching stable or detectable concentrations in water and/or sediment: phantolide (*personal care & household*); terbuthylazine (V-pattern), triadimenol, benalaxyl, triclosan-methyl, hexadecyltrimethylammonium (*pesticides/biocides*); daidzein (V-pattern), harman (*food, beverage & stimulants*); citalopram (*pharmaceuticals*); dibenz(a,h)anthracene (*PAHs*), 4‑hydroxyquinoline, perfluorodecanoic acid, ethylene glycol diphenylether (other). One compound was not detected at the compared time points (dinoseb (pesticides/biocides)). This means that a contamination of the FHM with these compounds might be overlooked or underestimated, if only analysis of water and/or sediment is applied. While it might not be surprising for daidzein and harman as natural occurring compounds, it seems relevant for the other substances.

# S13. BCF and BSAF calculation

The experimental bioconcentration factor BCF and the apparent biota-sediment accumulation ratio BSAF were calculated from measured water (*c*_w_), sediment (*c*_sed_) and FHM (*c*_FHM_) concentrations as follows:

$K_{\mathrm{OC}}= \frac{c_{\mathrm{oc}}}{c_{w}}$ (3),

$\mathrm{BCF}= \frac{c_{\mathrm{biota}}}{c_{w}}; here\mathrm{BCF}= \frac{c_{\mathrm{FHM}}}{c_{w}}$ (4),

$\mathrm{BSAF}= \frac{c_{\mathrm{biota}}}{c_{\mathrm{sed}}}; here\mathrm{BSAF}= \frac{c_{\mathrm{FHM}}}{c_{\mathrm{sed}}}$ (5)

For this study, these experimental ratios were calculated and compared to predicted steady-state BCF_eq_ and BSAF_eq_ in more detail to evaluate how well the measured concentration ratios agreed with thermodynamic equilibrium conditions.

The values for BCF_eq_ and BSAF_eq_ used in this study were derived from predicted thermodynamics, excluding metabolism, calculated with a simple mass balance model using the lipid-water distribution ratio *D*_lip/w_, and the protein-water distribution ratio *D*_prot/w_, Eq. 6 with f_lip_, f_prot_ and f_w_ being the fractions of lipid, protein, and water in the fish, respectively:

$\mathrm{BCF}_{\mathrm{eq}}= \frac{c_{\mathrm{biota}}}{c_{w}}{=f}_{\mathrm{lip}}D_{lip/w}+f_{\mathrm{prot}}D_{prot/w}+f_{w,w}$ (6)

For *D*_lip/w_ the membrane-lipid water distribution ratio was used and for *D*_prot/w_ the bovine serum albumin (BSA)-water distribution ratio *D*_BSA/w_ was used as a surrogate. The values for *D*_lip/w_ and *D*_BSA/w_ were taken from the dataset collated by Niu et al.^11^, which consists of both experimental and calculated values. Experimental data were favorably selected over calculated values, all of which are listed in the SM-B, Table S7. Predictions were based on the neutral species, where ionization-correction was performed as previously described^12^, assuming that the charged species partitions tenfold lower into membrane lipids than the neutral species and that protein binding is the same for neutral and charged species. Please note that such mass-balance models in the literature mostly do not have a term for protein binding because they are used mainly for very hydrophobic chemicals, where lipid-partitioning is the dominant process for bioconcentration. It is for more hydrophilic and ionizable organic chemicals that binding to proteins becomes more relevant and the term *f*_prot_*D*_prot/w_ must be included in the mass-balance model.^13^

The lipid fraction equals the individual lipid content of the FHM estimated gravimetrically using the extraction method by Smedes^14^ (SM-B, Table S5f); the protein fraction was taken from literature^15^ (average value for all FHMs: f_prot_ ≈ 0.09 ± 0.01), and from both of these values, the fraction of water (including also nonbinding materials such as carbohydrates and bones) in FHM was derived (eq. 7):

$f_{w,w}=1-f_{\mathrm{lip}}-f_{\mathrm{prot}}$ (7)

BCF_eq_ and BSAF_eq_ values were derived from predicted thermodynamics, excluding metabolism, calculated with a simple mass balance model using the lipid-water distribution ratio *D*_lip/w_, and the protein-water distribution ratio *D*_prot/w_. The values for *D*_lip/w_ and *D*_prot/w_ were taken from the dataset collated by Niu et al.^11^, which consists of both experimental and calculated values. The BSAF_eq_ was derived from the BCF_eq_ using the *K*_OC_ (eq. 8), also taken from Niu et al.^11^ complemented by values from EPISuite^54^ (SM-B, Table S7):

$\mathrm{BSAF}_{\mathrm{eq}}=\frac{c_{\mathrm{biota}}}{c_{\mathrm{sed}}}=\frac{\mathrm{BCF}_{\mathrm{eq}}}{K_{\mathrm{OC}}}$ (8)

Additionally, to obtain a better understanding of each compound’s concentration expected in the fish, an equilibrium log *c*_FHM_ was derived from the measured *c*_w_ and *c*_sed_ as well as the calculated BCF and BSAF values for every site and compared to the logarithmic measured maximal concentration in FHM for both deployment durations, i.e., 2-d and 21-d exposed FHM, complemented by the evaluation of MDLs of the QuEChERS method (SM-B, Table S11-S12).

$\mathrm{BSAF}_{\mathrm{eq}}=\frac{c_{\mathrm{biota}}}{c_{\mathrm{sed}}}=\frac{\mathrm{BCF}_{\mathrm{eq}}}{K_{\mathrm{OC}}}$ (8)

For substances not included in the dataset of Niu et al.,^11^ the dataset in this study was complemented by predicted values for BCF and BSAF from EPISuite^16^ (Table S7).

Additionally, to obtain a better understanding of each compound’s concentration expected in the fish, an equilibrium log *c*_FHM_ was derived from the measured *c*_w_ and *c*_sed_ as well as the calculated BCF and BSAF values for every site and compared to the logarithmic actual measured maximal concentration in 2 d and 21-d *c*_FHM_, complemented by the MDLs of the QuEChERS method. The resulting figures can be found in Section S12 (using data from SM-B, Table S11-S12).

# S14. Comparing predicted and experimental *c*_FHM_

As shown in Fig. 1, many substances were detected in water, sometimes at relatively high levels, but not in FHM at concentrations exceeding the control fish threshold. To get a better understanding of each compound’s concentration expected in the fish, an equilibrium *c*_FHM_ was derived from the measured *c*_w_ and *c*_sed_ as well as the calculated BCF/BSAF values (complemented from EPISuite in case of missing values) for every site and compared to the measured maximum concentration in 2-d and 21-d *c*_FHM_, complemented by the controls, the fish food (brine shrimp, crumbled starter feed = “trout chow”) and the MDL of the used QuEChERS method. All compounds are grouped by their compound class (Figure S7.1-S7.8: *personal care and household, pharmaceuticals, POPs, polymer additives, PAHs, pesticides/biocides, food beverage & stimulants, other*).

Figure S7.1 focuses on the substances detected at high concentrations in water, sediment and/or FHM, while a comprehensive overview broken down per use class are displayed in Fig. S7.27.8. Note that in these figures all the *c*_FHM_ from the different sites are displayed together with the *c*_FHM_ of the controls – meaning the control threshold is considered in the interpretation, but not in any way included in the figure itself.

At least one value for the 2-d or 21-d fish, an expected *c*_FHM_ could be derived for 27 substances at Creek, 24 at Pond S, 22 at Pond N, and 45 substances at the WWTP. In 61 cases (Creek: 11 / Pond S: 13 / Pond N: 15 / WWTP: 22), a *c*_FHM_ is was expected but not detected at either time point despite an MDL below the expected concentration. However, in 57 cases (16 / 11 / 7 / 23) with 30 substances a *c*_FHM_ at least at one time-point could be detected. The BCF_eq_ calculations are based on a simple mass balance/partitioning model and assumes no metabolism in fish, so non-detects might also be explained by metabolism/depuration of less persistent compounds.

If a substance accumulates, *c*_FHM_ should be similar or higher in the 21-d FHM in comparison to the 2-d FHM. This behavior was observed for 15 substances at Creek, five at Pond S, four at Pond N, and twelve substances at the WWTP. 17 substances showed increasing concentrations: denatonium, 1,3-diphenylguanidine, perfluorohexanoic acid, perfluorooctanesulfonic acid, N-cyclohexyl-2-benzothiazole-amine, piperonyl butoxide, dinoseb, 5-methyl-1H-benzotriazole, propranolol, diphenhydramine, metoprolol, 4‑(dimethylamino) pyridine, citalopram, lidocaine, terbuthylazine, triadimenol, and iminostilbene. Four compounds showed similar concentrations in 2-d and 21-d FHM at one or more sites, implying they reached a rather stable equilibrium/steady-state concentration in less than 2-d. These chemicals were tributyl phosphate, diphenhydramine, citalopram, and 4‑(dimethylamino) pyridine. Tebuconazole, tramadol, benzyldimethyldodecylammonium, benzyldimethyltetradecylammonium showed both similar or higher concentrations in the FHM between the two time points, depending on the site. Of the chemicals accumulated in fish, twelve (Creek: 6 / Pond S: 2 / Pond N: 1 / WWTP: 3) values for *c*_FHM_ (21-d) were below and 18 (10 / 1 / 3 / 4) above the expected *c*_FHM_. The largest discrepancy between expected and measured 21-d FHM concentrations, exceeding ≥ +1 log-unit were found for the charged chemicals dinoseb, diphenhydramine, metoprolol and 4-(dimethylamino) pyridine, perfluorooctanesulfonic acid, for which the mass balance model applies numerous assumptions and simplifications. Detected concentrations exceeding ≥ +1 log-unit imply an underestimation of accumulation potential, *c*_FHM_ ≤ -1 log-unit imply overestimation.

Eight compounds were only detected in 2-d FHM at one or more sites: fipronil, 7‑amino-4‑methylcoumarin, 2‑ and 4‑hydroxyquinoline, octyl-methoxycinnamate, triphenyl phosphate, propranolol, lauric isopropanolamide, and thus implying metabolism in the fish over longer exposure times: in fact, two transformation products of fipronil (fipronil sulfide and fipronil sulfone) were also detected in FHM, but not in water; 7-amino-4-methylcoumarin is a transformation product of the dye 7-diethylamino-4-methylcoumarin^17^, which was detected in water at the same site (WWTP). 2‑ and 4‑Hydroxyquinoline are likely to be metabolized by microbiota^18^, and therefore they are not expected to be very stable. Triphenyl phosphate biodegrades relatively quickly^19^ as does the UV filter octyl-methoxycinnamate. In case of the drug propranolol, rapid depuration/metabolisation was detected in daphnia^20^. For the surfactant lauric isopropanolamide, no evidence of metabolism could be found in literature. In 18 cases (seven at Creek, two at Pond S, three at Pond N, six at WWTP), *c*_FHM_ was >MDL despite the fact that the expected concentrations in FHM were below the MDL. In 19 cases (3, 5, 1, 10) an expected log *c*_FHM_ could not be derived for every site.

Four compounds were detected in fish at several sites, but at different sites/times: metoprolol (only 2-d at WWTP, only 21-d at Creek), daidzein (at both Ponds only at 2-d, additionally at Creek at 21-d, but lower), tebuconazole (only 2-d at Pond S; both 2-d and 21-d at Creek and WWTP), and terbuthylazine (2-d at both Ponds, 21-d at WWTP). This inconsistency might be explained for three substances by fluctuatingn/different concentrations (metoprolol) in the water and/or concentrations close to the MDL (daidzein, tebuconazole), but not for terbuthylazine, which is present at Pond N and WWTP at rather similar concentrations. One reason for the discrepancy regarding this compound as well as for the high number of expected but not detected compounds at Pond N might be the low lipid content measured in the FHM from this site.


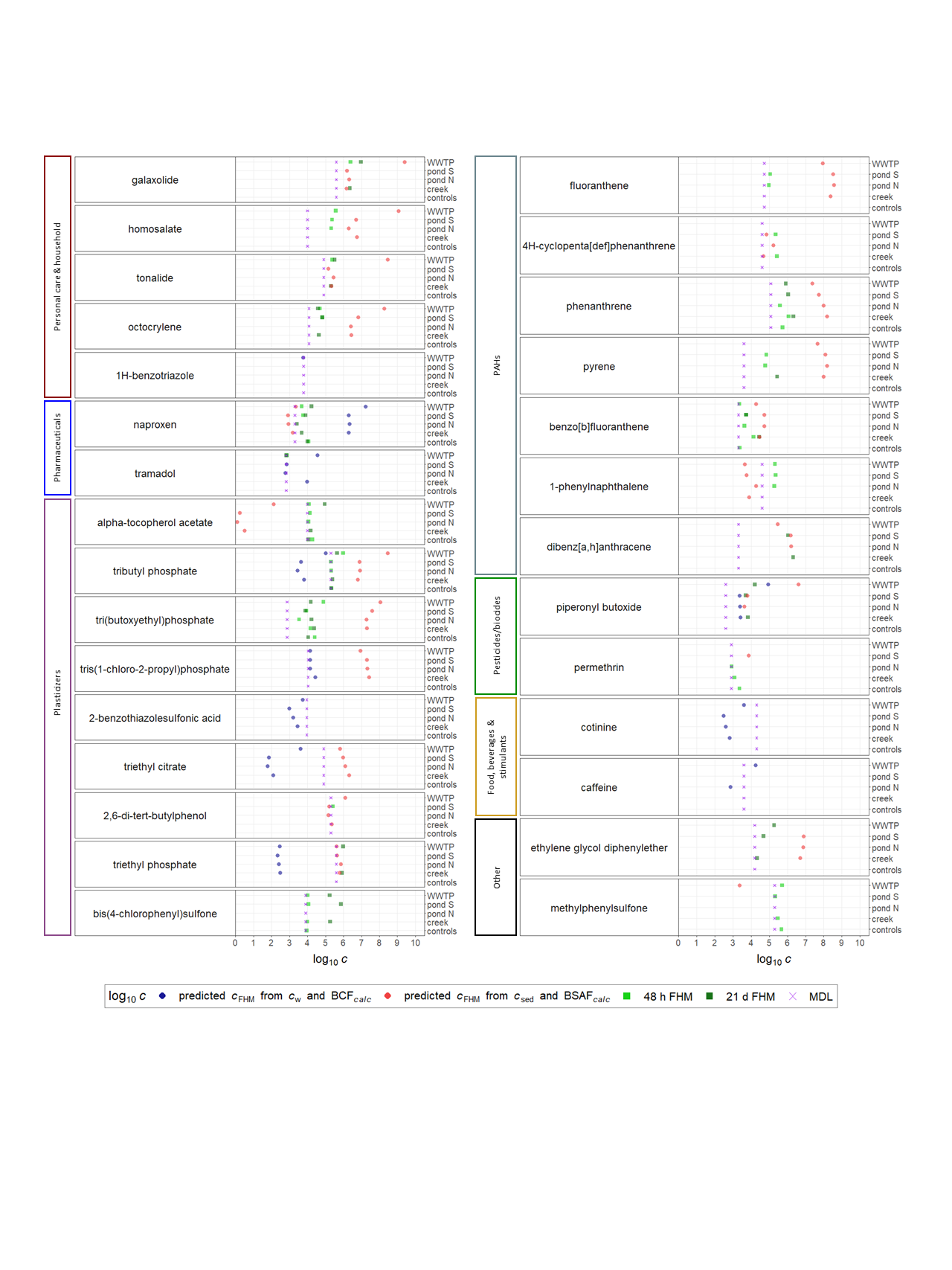


**Figure S7.1:** Expected log c_FHM_, at Creek, Pond S, Pond N, and WWTP derived from BCF and c_w_ / BSAF and c_sed_; as well as the controls and the MDL of the used QuEChERS method. Data from Table S11-S12.

For some substances, namely, 1H-benzotriazole, 2-benzothiazolesulfonic acid, 2,6-di-tert-butylphenol, and cotinine, the MDL of the extraction method used for FHM was above/close to one or both predicted *c*_FHM_ (Figure S5a, SM-B Table S11-S12). In several other cases, namely naproxen, α‑tocopherol acetate, tributyl phosphate, tri(butoxyethyl)phosphate, permethrin and methylphenylsulfone, the predicted *c*_FHM_ were close to the experimental *c*_FHM_, but they also showed similar *c*_FHM_ in the controls.

Homosalate and three PAHs (fluoranthene, 4H-cyclopenta(def)phenanthrene, 1-phenylnaphthalene), were found only in 2 d FHM; the absence of 21-d detects may indicate induction of metabolism and effective depuration by metabolism of these compounds, which is also supported by (unpublished) data showing CYP induction in the FHM from the sites.

In most instances, the measured *c*_FHM_ values were below the *c*_FHM_ predicted from sediment or water, which might indicate that the compounds had not reached equilibrium between sediment and water and, consequently, the fish tissue. A possible contributor to the lack of equilibrium in the system could be that the fish cages were placed at some distance above the sediment surface. In cases where *c*_FHM_ was above the *c*_FHM_ predicted by sediment the substance might be more recently emitted to the water phase and therefore could not accumulate in the sediment as much as in the fish. Exceptions to this are piperonyl butoxide and naproxen, where the *c*_FHM_ predicted by sediment was below the experimental *c*_FHM_ and below the *c*_FHM_ predicted by equilibrium with water.


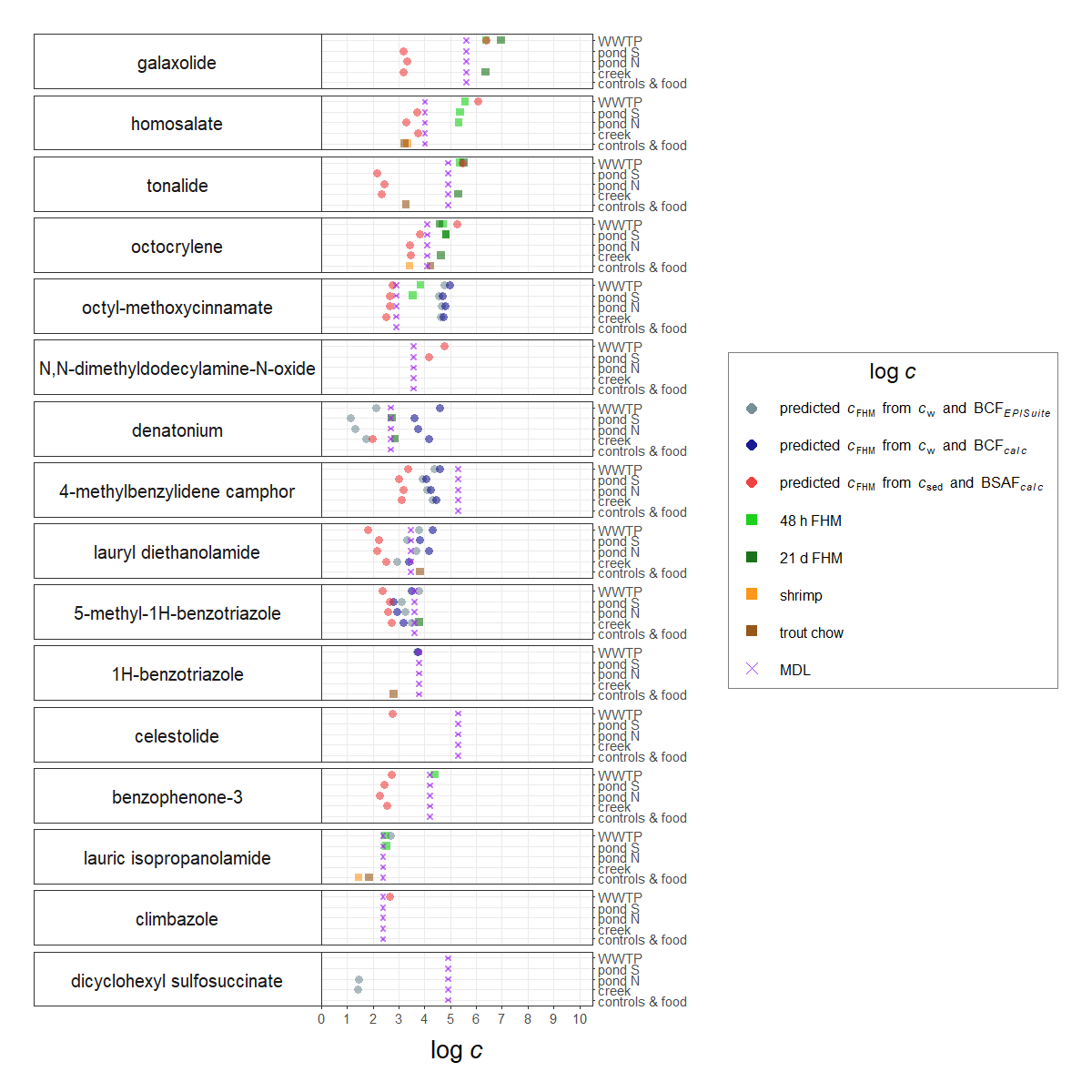


**Figure S7.2:** Expected log c_FHM_, at Creek, Pond S, Pond N, and WWTP derived from BCF and c_w_ / BSAF and c_sed_; personal care and household. Data from Table S10-S11.


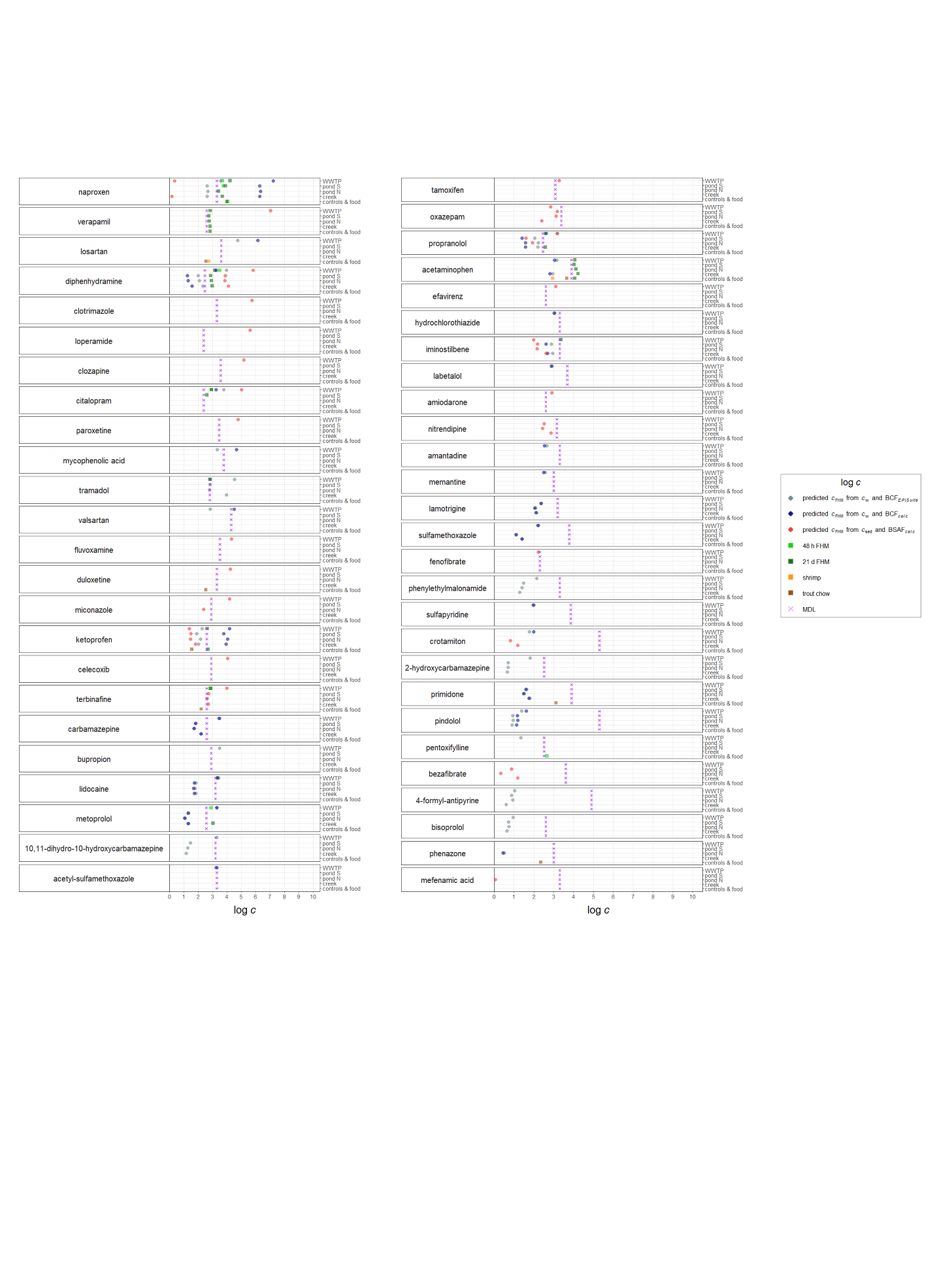


**Figure S7.3:** Expected log c_FHM_, at Creek, Pond S, Pond N, and WWTP derived from BCF and c_w_ / BSAF and c_sed_; pharmaceuticals. Data from Table S10-S11.


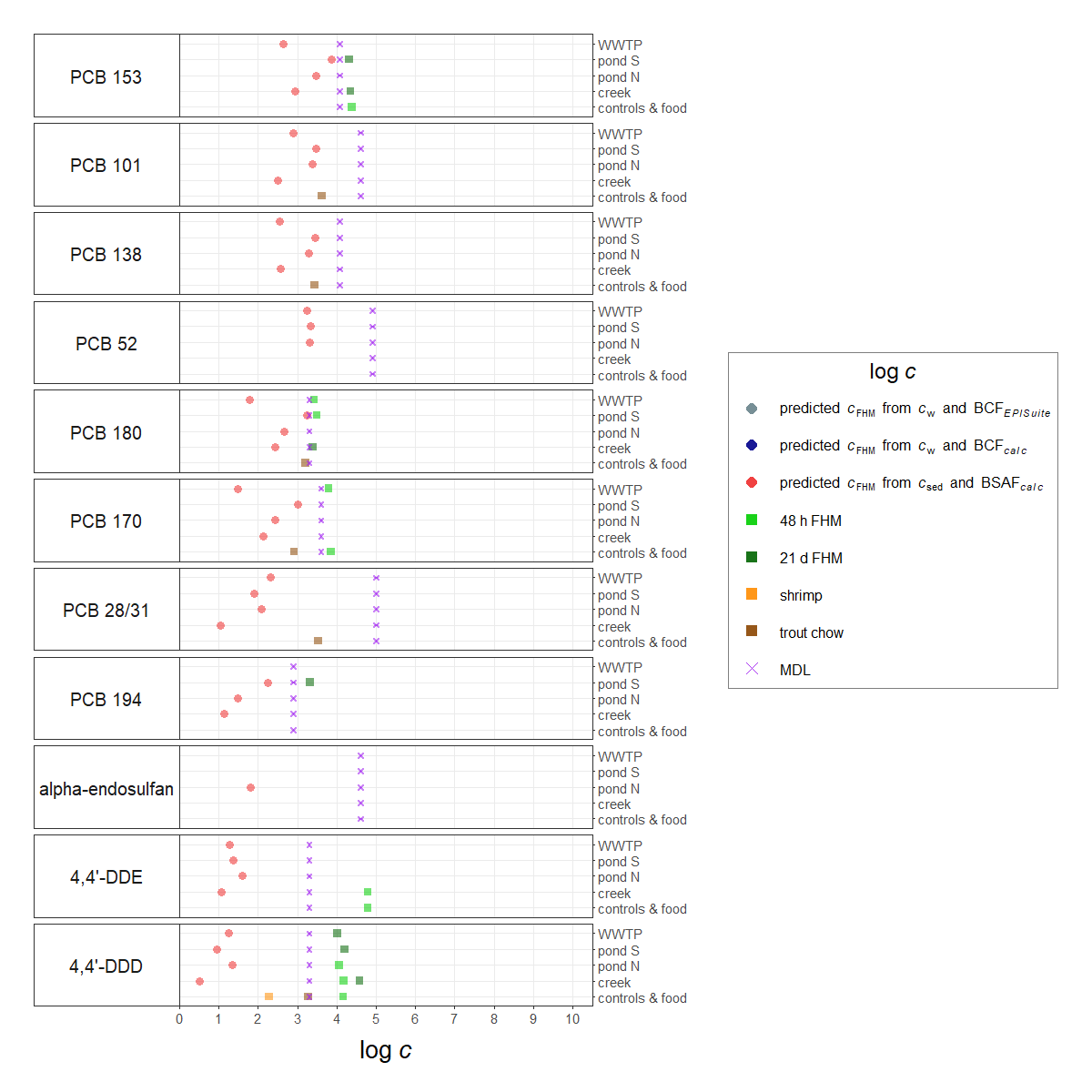


**Figure S7.4:** Expected log c_FHM_, at Creek, Pond S, Pond N, and WWTP derived from BCF and c_w_ / BSAF and c_sed_; POPs. Data from Table S10-S11.


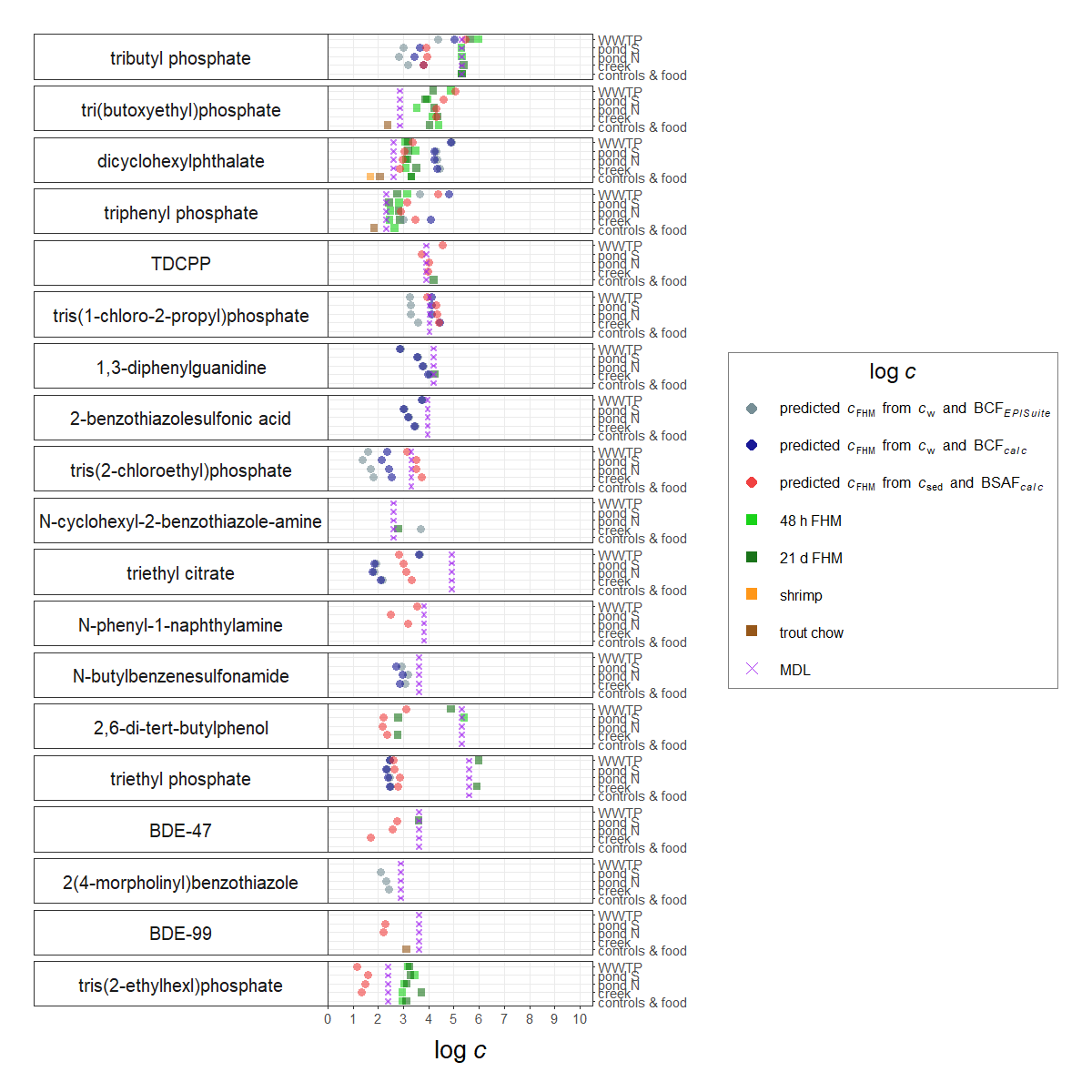


**Figure S7.5:** Expected log c_FHM_, at Creek, Pond S, Pond N, and WWTP derived from BCF and c_w_ / BSAF and c_sed_; polymer additives. Data from Table S10-S11.


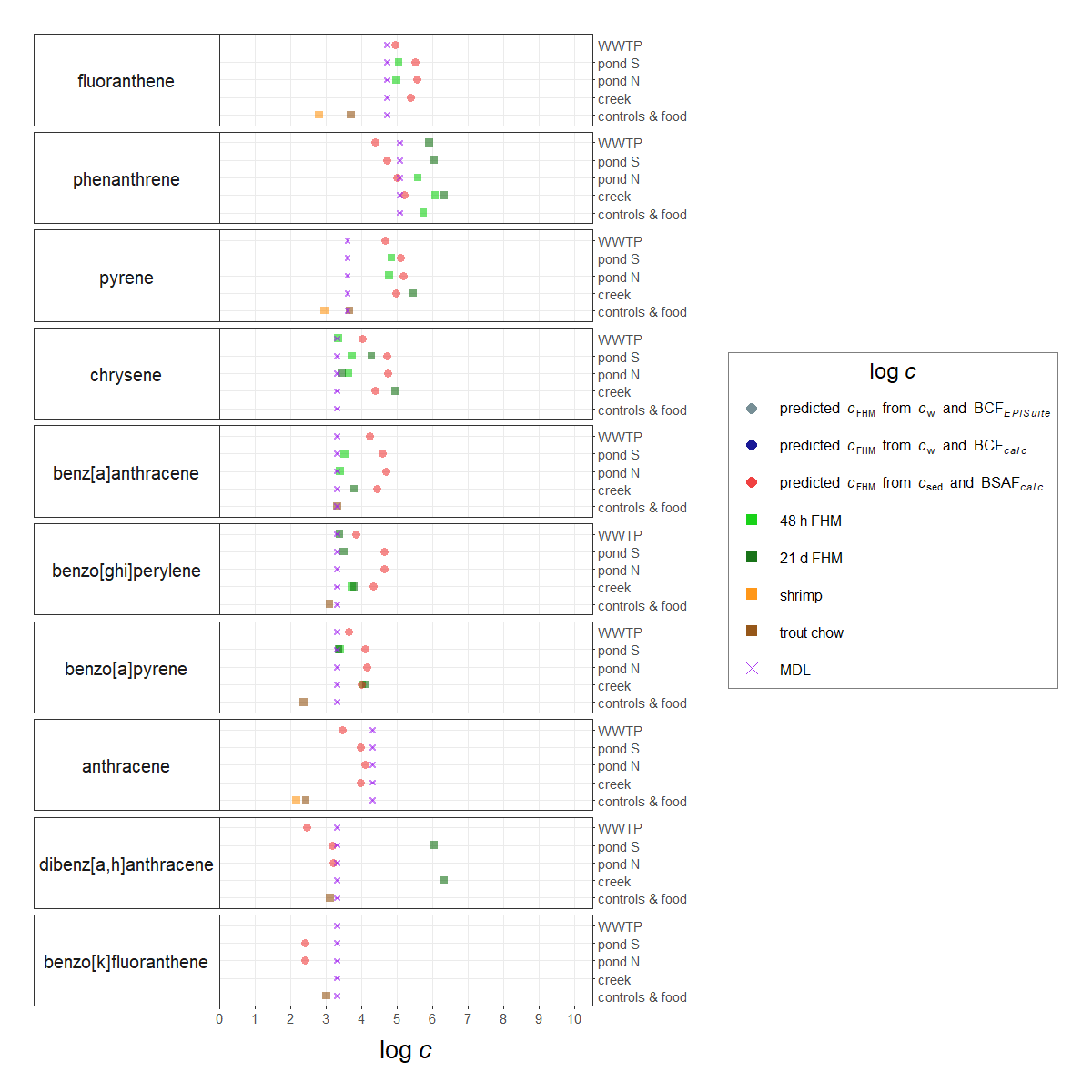


**Figure S7.6:** Expected log c_FHM_, at Creek, Pond S, Pond N, and WWTP derived from BCF and c_w_ / BSAF and c_sed_; PAHs. Data from Table S10-S11.


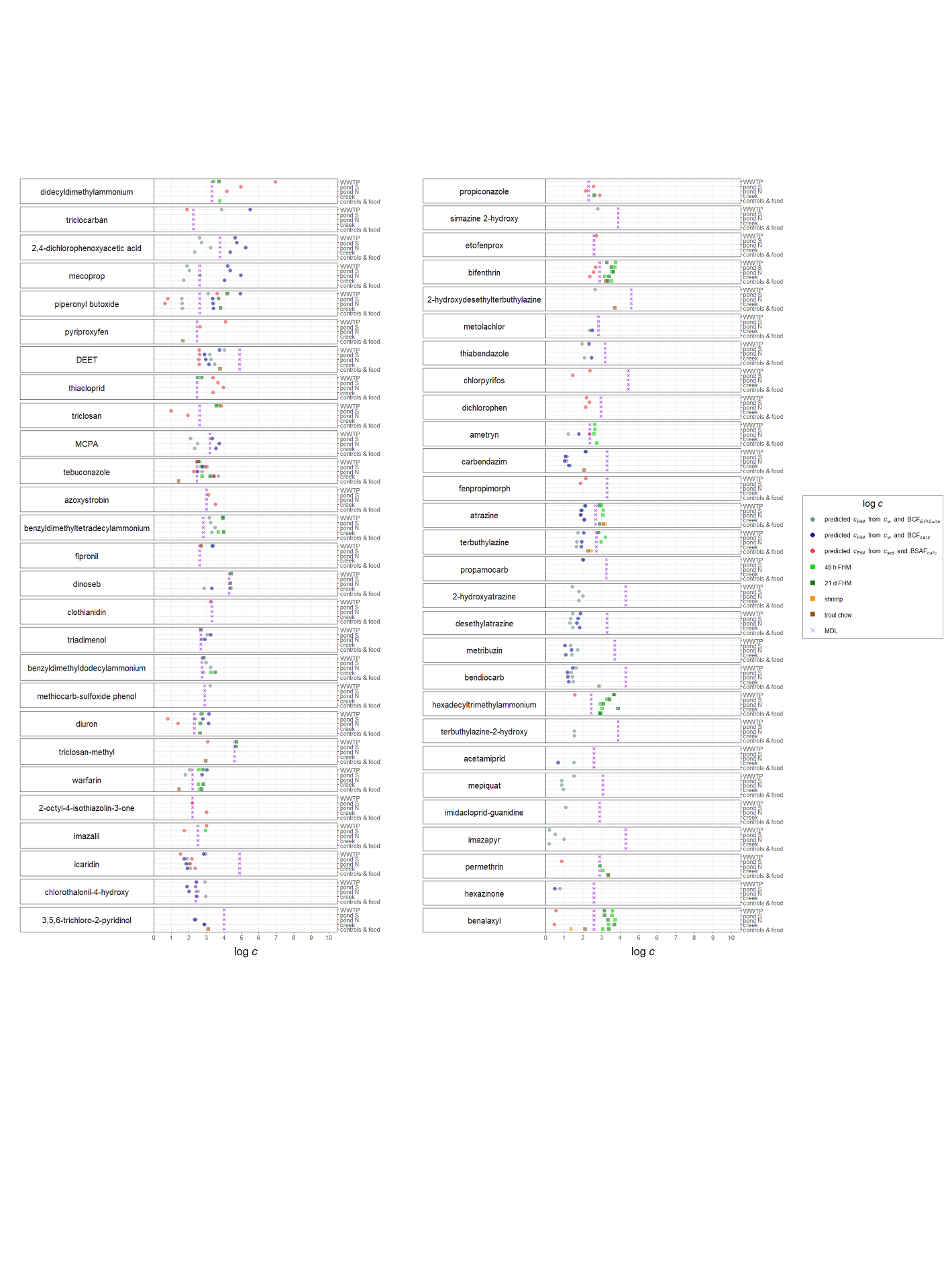


**Figure S7.7:** Expected log c_FHM_, at Creek, Pond S, Pond N, and WWTP derived from BCF and c_w_ / BSAF and c_sed_; pesticides/biocides. Data from Table S10-S11.


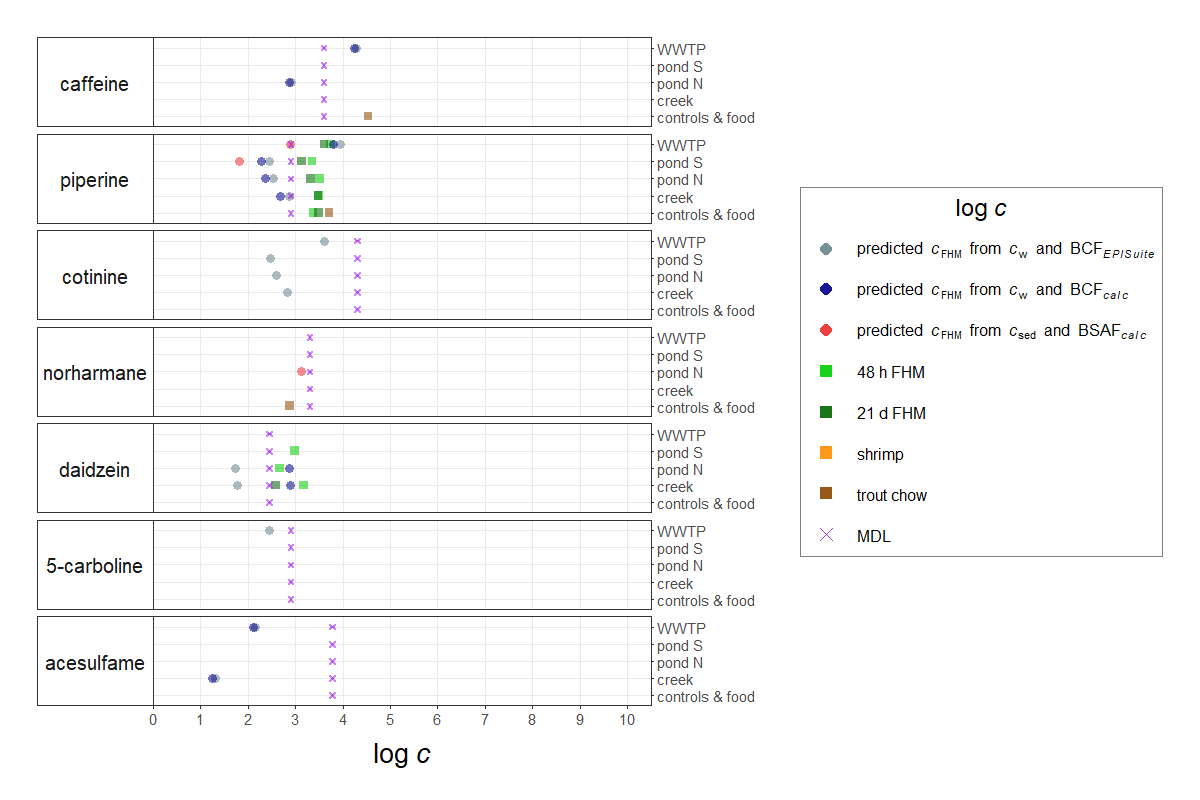


**Figure S7.8:** Expected log c_FHM_, at Creek, Pond S, Pond N, and WWTP derived from BCF and c_w_ / BSAF and c_sed_; food, beverage & stimulants. Data from Table S10-S11.


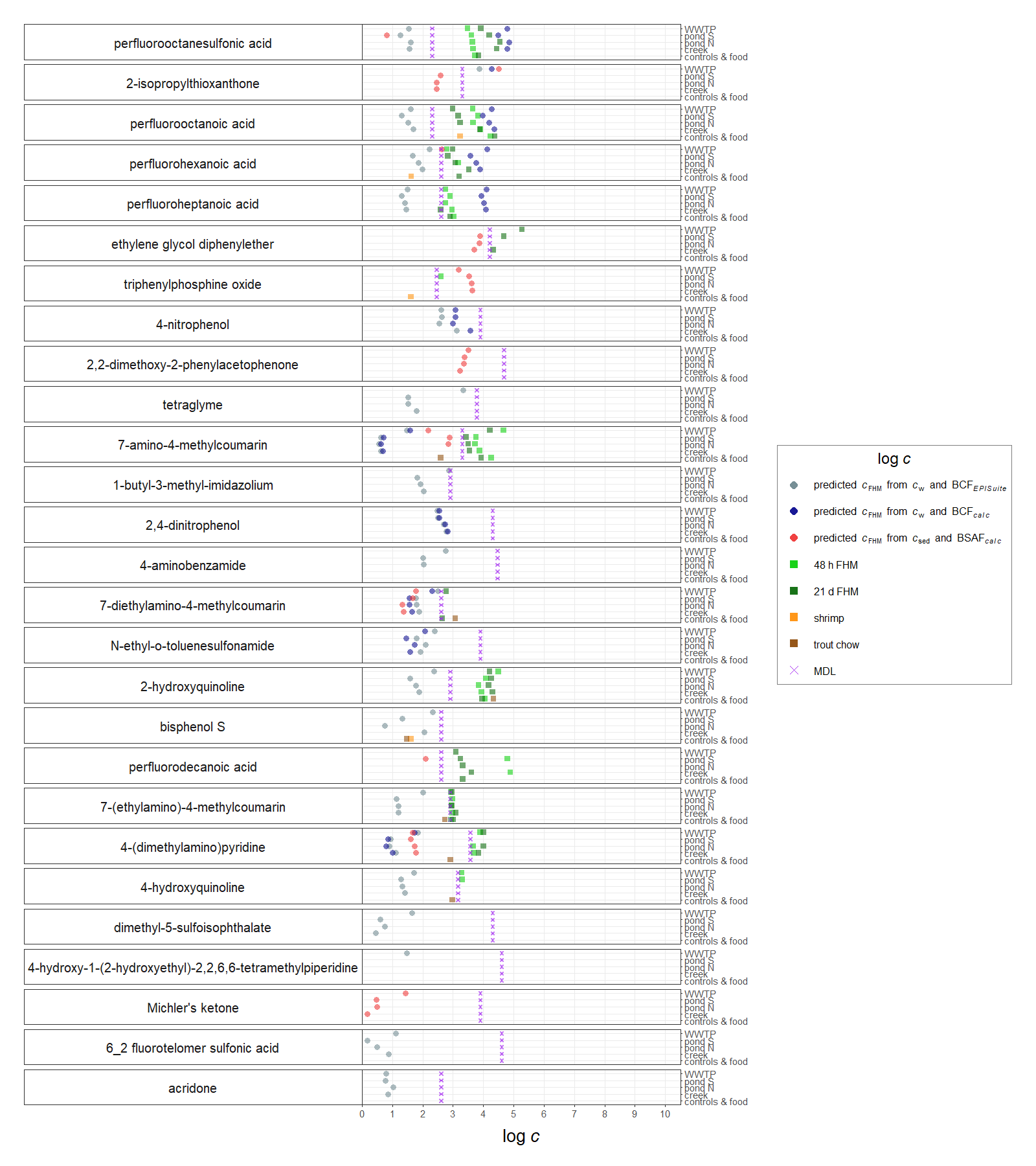


**Figure S7.9:** Expected log c_FHM_, at Creek, Pond S, Pond N, and WWTP derived from BCF and c_w_ / BSAF and c_sed_; other. Data from Table S10-S11.

# S15. Comparing predicted and experimental BCF and BSAF with *c*_FHM_ / c_w_^-1^ and *c*_FHM_ / c_sed_^-1^

## S15.1 Distribution between water and sediment

For 45 substances determined in water and sediment, an experimental concentration ratio c_sed_ × c_w_^‑1^ was calculated and plotted against the consensus log *K*_OC_ values (Fig. S6a, S7a; *K*_OC_ in SM‑B, Table S8, concentration ratios in Tables S11-12). The distance to the 1-to-1 line (solid line in Figure S6 and S7) indicates how far the ratio of the concentrations deviated from thermodynamic equilibrium. Overall, 68 of 111 datapoints (61%) and at least one datapoint per compound in 28 of the 45 pairs were within ± 1 log-unit of the 1-to-1 line (62% of compounds, broken lines in Figure S6 and S7). Here, 41 of 43 of the datapoints outside this range and the majority of those within the ± 1 log-interval (52/68) were above the 1-to-1 line, implying that the sediment was acting as a source of these contaminants to the water phase. This is consistent with findings of a previous study in a river under baseflow conditions where sediment and suspended particulate matter acted as sources of chemicals to water.^21^


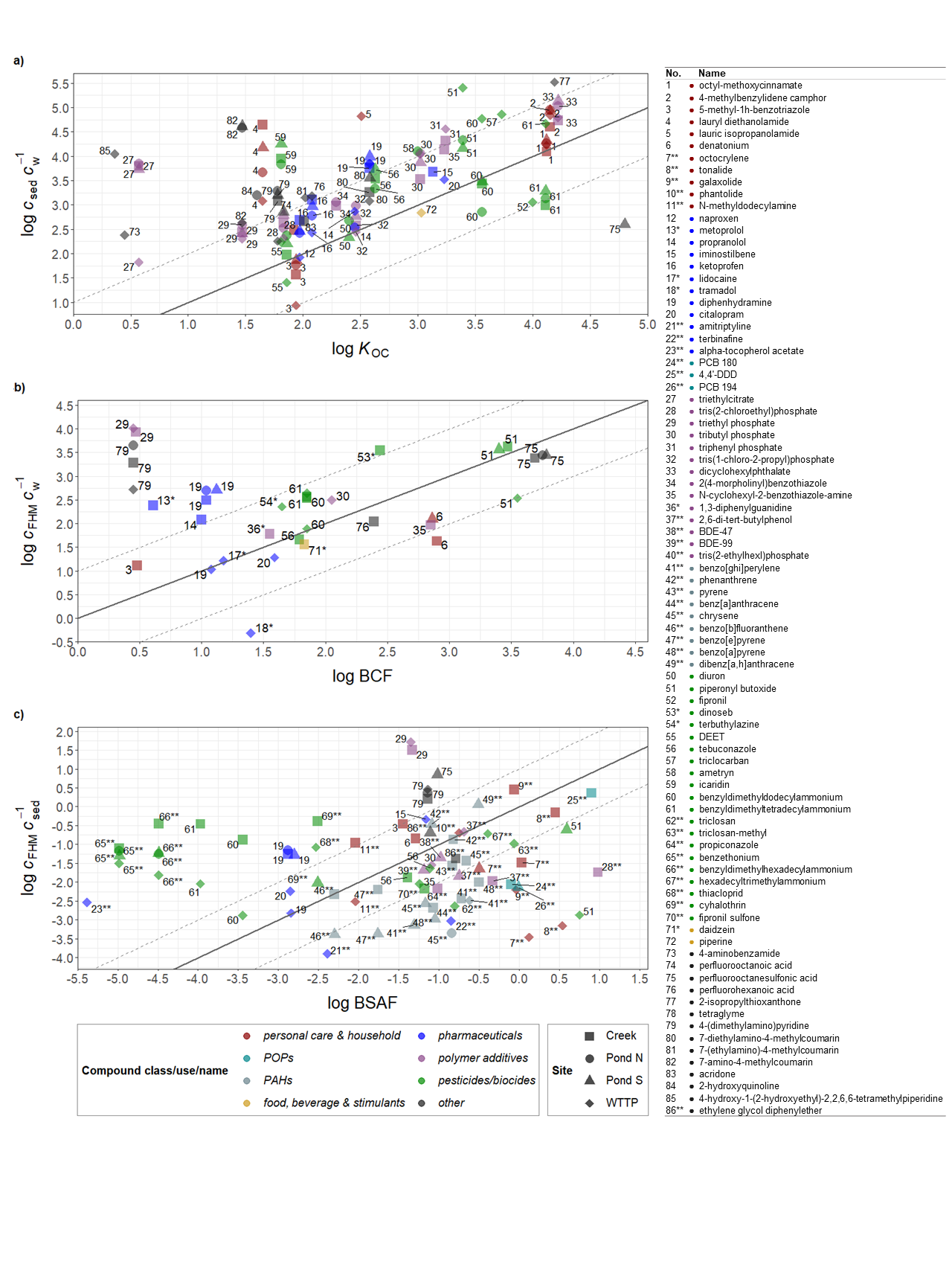


**Figure S8a-c**. a) log c_sed_ × c_w_^-1^ plotted against log K_OC_; b) log c_FHM_ × c_w_^-1^ plotted against log BCF; c) log c_FHM_ × c_sed_^-1^ plotted against log BSAF. The sites are displayed with different symbols, the compound classes with different colors, representing the categories used above. The plots show a 1:1-correlation within ± 1 log-unit for 63% of the datapoints: three datapoints log c_FHM_ × c_w_^-1^ < log BCF+1 and 11 datapoints log c_FHM_ × c_w_^-1^ > log BCF-1. For substances marked with a single asterisk, no c_sed_, and for substances marked with two asterisks, no c_w_ was measured. Data in SM-B, Tables S8, S11 and S12.


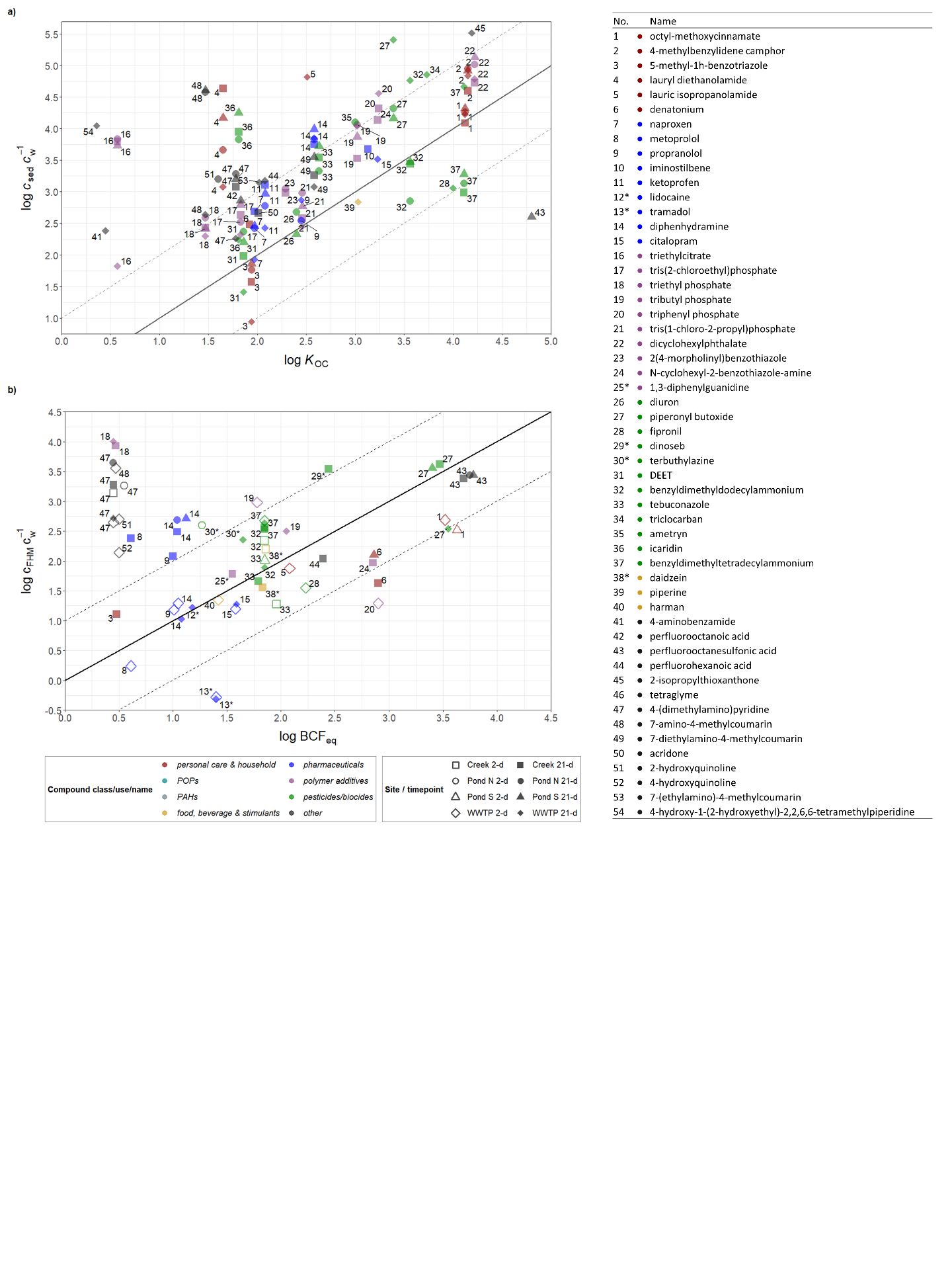


**Figure S9a-b.** a) log c_sed_ × c_w_^-1^ plotted against log K_OC_; b) log c_FHM_ × c_w_^-1^ plotted against log BCF_eq_. The sites and timepoints are displayed with different symbols, the compound classes with different colors, representing the categories used above. Data in SM-B, Tables S8, S11 and S12.

## S15.2 Concentration ratios between fish and water as compared to predicted BCF_eq_ values

23 substances were detected in 21-d FHM and in water, for which an experimental value for log_10_ (*c*_FHM_ × *c*_w_^-1^) could be calculated. The results were plotted (Fig. 5b and 6b) against the predicted log BCF_eq_ (see “BCF and BSAF calculation” in section S7). Overall, 60% of the datapoints and at least one datapoint for 71% of compounds were situated within the confidence interval, which indicates that the 21-d caging period for FHM was sufficient for these compounds to accumulate to the extent predicted by the simple equilibrium partitioning model. There are notable outliers in the analysis. The high measured ratios of *c*_FHM_ × *c*_w_^-1^ of triethyl phosphate and 4-(dimethylamino)pyridine as compared to the predicted log BCF might indicate that these are transformation products of unknown origin, substances with very unusual accumulation behavior, or artifacts caused by erroneous predicted BCF values.

Additionally, in Fig. 6a and 6b the substances detected at 2-d in FHM were also included. It is visible that substances already are accumulated to a similar degree as many of the substances found in the 21‑d FHM.

## S15.3 Concentration ratios between fish and sediment as compared to predicted BSAF values

As the cages were deployed at some distance (approx. 1 m below the water surface) above the underlying sediment of the various waterbodies, the FHM were not expected to achieve equilibrium with sediment-associated contaminants as quickly as if they had been in direct contact with the sediment. Due to uncertainties as to the true equilibrium between contaminants in the FHM relative to sediment, the BSAF values serve more as a basis upon which to compare accumulation potential from sediment versus water, which was addressed before.^22^ This is reflected by Fig. 5c, which shows the different enrichment tendencies of the substances rather than the expected equilibrium distribution between the two compartments.

Overall, 48 substances were detected both in the 21-d FHM and in sediment, resulting in 81 values for *c*_FHM_ × *c*_sed_^-1^ at the different sites. For 28 of the 48 substances (58%), at least one datapoint was within one log-unit from the 1-to-1 line, compared to the other 20 substances where all datapoints lay outside of this interval. Regarding the entire dataset only 38% of the data points agreed to that extent. Reasons for some of the outliers might be the high uncertainty in calculated BSAF for permanently charged compounds (e.g., benzyldimethyldodecylammonium, benzyldimethyltetradecylammonium, benzethonium, benzyldimethylhexadecylammonium) or very high concentrations in sediment at the WWTP (in particular, octocrylene, tonalide, piperonyl butoxide) leading to smaller than predicted values of log *c*_FHM_ × *c*_sed_^-1^. While sediment may act as a source of these compounds, the overlying water to which the fish were exposed may not have been in equilibrium with the sediment. Another possible explanation might be that the fish did not reach predicted concentrations in the time period of 21 days, as these are reflecting an overestimated bioaccumulation potential of a substance.

Generally, the comparison between predicted and measured values in FHM indicates that for compounds for which the MDL of the QuEChERS extraction was low enough, many of the substances found in water could also be detected in FHM. The uptake of several compounds was confirmed and characterized, showing expected accumulation behavior (based on hydrophobicity) for 21/30 substances (36/57 detects including substances with more than one finding), but they were often found at lower concentrations than the predicted log c_FHM_ suggested.

# S16 Comparison contamination patterns in FHM with findings from the literature

In the following section a selection of substances detected at higher concentrations (cf. 3.1), is discussed more in detail in context of previously published work.

*Legacy compounds:*

*POPs* and *PAHs* showed only A-patterns and both A- and E-patterns, respectively, slightly extenuated by non-detects at pond N (mentioned reasons cf. 4.1) and V‑patterns at single sites. That *POPs* were detected in A-patterns is not surprising, as those compounds are known to accumulate in biota ^17-19^. Some *PAHs* readily metabolized by fish would be expected to have E-patterns, whereas the ones not metabolized by fish would show an A-pattern ^46, 47^. This fits the findings quite well as fluoranthene, which has been shown to be metabolized by fish *in vitro*, showed an E-pattern consistent with expectations from literature ^46^. Pyrene, benzo(b)fluoranthene, phenanthrene and dibenz(a,h)anthracene, are known to bioaccumulate in fish ^48, 49^. While benzo(b)fluoranthene, phenanthrene and dibenz(a,h)anthracene accumulated (A‑pattern), supporting the findings in this study. The detects of pyrene represented a V-pattern and were inconclusive.

*Recent/continuously discharged micropollutants:*

The *personal care & household* substances octocrylene, galaxolide, and tonalide appeared to show an A-pattern. The observation that octocrylene has bioaccumulation potential agrees with findings in other studies ^50, 51^. Galaxolide is considered to be moderately persistent ^52^ and tonalide is easily degraded. An apparent elimination of the UV filter homosalate in FHM (E-pattern) also agrees with reports that homosalate is metabolized ^53^

The abundance of the *pharmaceuticals* tramadol and α-tocopherol acetate resembled an A-pattern, the latter only if the concentration above the FHM control threshold is considered. Tramadol has been found to be metabolized by fish ^54^, but uptake might be fast enough to maintain high concentrations in FHM, made possible due to constant presence in the water (c.f. 3.2 and 4.3.3). α‑Tocopherol acetate, a synthetic supplement for vitamin E used for fish nutrition ^55, 56^ and in cosmetics, is hydrolyzed to vitamin E in fish ^56^. Therefore, appearing in an A-pattern might be false-positive – it occurred at all sites and in controls at varying timepoints, the sole detect above the threshold at WWTP might be an outlier or resulting from a short-term uptake (not verifiable because of missing detects in water).

*Polymer additives:* 2,6-Di-tert-butylphenol showed an E-pattern, which reflects its reported biotransformation in fish ^57^, but disagrees with an accumulation reported in liver of a marine fish species ^58^. Tri(butoxyethyl) phosphate was detected in an E-pattern, but with relevant concentrations present in the FHM controls. Tributyl phosphate, triethyl phosphate, and bis(4‑chlorophenyl) sulfone represented an A-pattern. For triethyl phosphate it was previously reported not to bioaccumulate in aquatic organisms ^59^. Tris(1‑chloro-2-propyl) phosphate, which was detected only in water was also metabolized ^60^, which could explain the lack of detection in FHM.

*Pesticides/biocides*: Piperonyl butoxide, which is a synergist used in formulations to inhibit metabolism of the active pesticide, thereby enhancing its persistence and toxicity ^61, 62^ is not expected to bioaccumulate in fish due to fast elimination ^63^. Despite the latter, it occurred only at 21‑d, thus representing an A-pattern in FHM.

*Food, beverage & stimulants*: Caffeine and cotinine (a nicotine metabolite) were only found in water, but at all sites with the maximum concentration at WWTP. Caffeine is widely detected in waterbodies in USA and Europe ^64^, and may negatively affect aquatic life ^65, 66^. Cotinine was not found in rainbow trout (*Oncorhynchus mykiss*) after exposure to leachates of cigarette butts ^67^, but was found in FHM after exposure to nicotine ^67^. Even though that study used FHM and a similar analytical method as our study, the time frame of 24 h for their experiments was rather short compared to our exposure period of 2‑d/21‑d. Despite high concentrations in water neither chemical was detected in FHMs.

*Other*: Ethylene glycol diphenyl ether showed an A-pattern in the FHM. To our knowledge, it has previously neither been investigated nor reported to be bioaccumulative.

# References

(1) Ankley, G. T.; Berninger, J. P.; Blackwell, B. R.; Cavallin, J. E.; Collette, T. W.; Ekman, D. R.; Fay, K. A.; Feifarek, D. J.; Jensen, K. M.; Kahl, M. D.; et al. Pathway-Based Approaches for Assessing Biological Hazards of Complex Mixtures of Contaminants: A Case Study in the Maumee River. *Environ Toxicol Chem* **2021**, *40* (4), 1098-1122. DOI: 10.1002/etc.4949

(2) Kahl, M. D.; Villeneuve, D. L.; Stevens, K.; Schroeder, A.; Makynen, E. A.; LaLone, C. A.; Jensen, K. M.; Hughes, M.; Holmen, B. A.; Eid, E.; et al. An inexpensive, temporally integrated system for monitoring occurrence and biological effects of aquatic contaminants in the field. *Environ Toxicol Chem* **2014**, *33* (7), 1584-1595. DOI: 10.1002/etc.2591

(3) Jahnke, A.; Sobek, A.; Bergmann, M.; Braunig, J.; Landmann, M.; Schafer, S.; Escher, B. I. Emerging investigator series: effect-based characterization of mixtures of environmental pollutants in diverse sediments. *Environ Sci Process Impacts* **2018**, *20* (12), 1667-1679. DOI: 10.1039/c8em00401c

(4) Jahnke, A.; Mayer, P.; McLachlan, M. S. Sensitive equilibrium sampling to study polychlorinated biphenyl disposition in Baltic Sea sediment. *Environ Sci Technol* **2012**, *46* (18), 10114-10122. DOI: 10.1021/es302330v

(5) Team, U. E. C. M. Definition and Procedure for the Determination of the Method Detection Limit. Water, U. E. O. o., Ed.; US EPA: 2016.

(6) Baumard, P. B., H.; Garrigues, P.; Sorbe, J. C.; Burgeot, T.; Bellocq, J. Concentrations of pahs 876 (polycyclic aromatic hydrocarbons) in various marine organisms in relation to those in sediments and to 877 trophic level. *Marine Pollution Bulletin* **1998**, *36*, 951-960.

(7) Houde, M.; De Silva, A. O.; Muir, D. C.; Letcher, R. J. Monitoring of perfluorinated compounds in aquatic biota: an updated review. *Environ Sci Technol* **2011**, *45* (19), 7962-7973. DOI: 10.1021/es104326w

(8) Jamieson, A. J. M., T.; Piertney, S. B.; Fujii, T.; Zhang, Z. L. Bioaccumulation of persistent organic 882 pollutants in the deepest ocean fauna. *Nature Ecology & Evolution* **2017**, *1*.

(9) Hawker, D. W. C., Des W. Bioconcentration of lipophilic compounds by some aquatic organisms. *Ecotoxicology and environmental safety* **1985**, *11*, 184-197.

(10) Choi, H.; Al-Abed, S. R. PCB congener sorption to carbonaceous sediment components: Macroscopic comparison and characterization of sorption kinetics and mechanism. *J Hazard Mater* **2009**, *165* (1-3), 860-866. DOI: 10.1016/j.jhazmat.2008.10.100

(11) Niu, L.; Carmona, E.; Konig, M.; Krauss, M.; Muz, M.; Xu, C.; Zou, D.; Escher, B. I. Mixture Risk Drivers in Freshwater Sediments and Their Bioavailability Determined Using Passive Equilibrium Sampling. *Environ Sci Technol* **2020**, *54* (20), 13197-13206. DOI: 10.1021/acs.est.0c05124

(12) Escher, B. I.; Abagyan, R.; Embry, M.; Kluver, N.; Redman, A. D.; Zarfl, C.; Parkerton, T. F. Recommendations for Improving Methods and Models for Aquatic Hazard Assessment of Ionizable Organic Chemicals. *Environ Toxicol Chem* **2020**, *39* (2), 269-286. DOI: 10.1002/etc.4602

(13) Baumer, A.; Jasch, S.; Ulrich, N.; Bechmann, I.; Landmann, J.; Escher, B. I. Kinetics of Equilibrium Passive Sampling of Organic Chemicals with Polymers in Diverse Mammalian Tissues. *Environ Sci Technol* **2021**, *55* (13), 9097-9108. DOI: 10.1021/acs.est.1c01836

(14) Smedes, F. Determination of total lipid using non-chlorinated solvents. *Analyst* **1999**, *124* (11), 1711-1718, 10.1039/A905904K. DOI: 10.1039/A905904K

(15) Lazaro-Cote, A.; Sadoul, B.; Jackson, L. J.; Vijayan, M. M. Acute stress response of fathead minnows caged downstream of municipal wastewater treatment plants in the Bow River, Calgary. *PLoS One* **2018**, *13* (6), e0198177. DOI: 10.1371/journal.pone.0198177

(16) Estimation Programs Interface Suite™ for Microsoft® Windows, v 4.11. (accessed.

(17) Muschket, M.; Brack, W.; Inostroza, P. A.; Beckers, L. M.; Schulze, T.; Krauss, M. Sources and Fate of the Antiandrogenic Fluorescent Dye 4-Methyl-7-Diethylaminocoumarin in Small River Systems. *Environ Toxicol Chem* **2021**, *40* (11), 3078-3091. DOI: 10.1002/etc.5181

(18) BLOCK, D. W.; LINGENS, F. Microbial Metabolism of Quinoline and Related Compounds. XIII. Purification and Properties of 1H-4-Oxoquinoline Monooxygenase from Pseudomonas putida Strain 33/1. **1992**, *373* (1), 249-254. DOI: doi:10.1515/bchm3.1992.373.1.249

(19) van der Veen, I.; de Boer, J. Phosphorus flame retardants: properties, production, environmental occurrence, toxicity and analysis. *Chemosphere* **2012**, *88* (10), 1119-1153. DOI: 10.1016/j.chemosphere.2012.03.067

(20) Jeong, T. Y.; Kim, T. H.; Kim, S. D. Bioaccumulation and biotransformation of the beta-blocker propranolol in multigenerational exposure to Daphnia magna. *Environ Pollut* **2016**, *216*, 811-818. DOI: 10.1016/j.envpol.2016.06.051

(21) Niu, L.; Ahlheim, J.; Glaser, C.; Gunold, R.; Henneberger, L.; Konig, M.; Krauss, M.; Schwientek, M.; Zarfl, C.; Escher, B. I. Suspended Particulate Matter-A Source or Sink for Chemical Mixtures of Organic Micropollutants in a Small River under Baseflow Conditions? *Environ Sci Technol* **2021**, *55* (8), 5106-5116. DOI: 10.1021/acs.est.0c07772

(22) Jahnke, A.; MacLeod, M.; Wickstrom, H.; Mayer, P. Equilibrium sampling to determine the thermodynamic potential for bioaccumulation of persistent organic pollutants from sediment. *Environ Sci Technol* **2014**, *48* (19), 11352-11359. DOI: 10.1021/es503336w
